# The impact of alternative delivery strategies for novel tuberculosis vaccines in low-income and middle-income countries: a modelling study

**DOI:** 10.1016/S2214-109X(23)00045-1

**Published:** 2023-03-14

**Authors:** Rebecca A Clark, Christinah Mukandavire, Allison Portnoy, Chathika K Weerasuriya, Arminder Deol, Danny Scarponi, Andrew Iskauskas, Roel Bakker, Matthew Quaife, Shelly Malhotra, Nebiat Gebreselassie, Matteo Zignol, Raymond C W Hutubessy, Birgitte Giersing, Mark Jit, Rebecca C Harris, Nicolas A Menzies, Richard G White

**Affiliations:** a TB Modelling Group and TB Centre, London School of Hygiene & Tropical Medicine, London, UK; b Centre for the Mathematical Modelling of Infectious Diseases, London School of Hygiene & Tropical Medicine, London, UK; c Department of Infectious Disease Epidemiology, London School of Hygiene & Tropical Medicine, London, UK; d Vaccine Centre, London School of Hygiene & Tropical Medicine, London, UK; e Center for Health Decision Science, Harvard TH Chan School of Public Health, Boston, MA, USA; f Department of Global Health and Population, Harvard TH Chan School of Public Health, Boston, MA, USA; g Department of Mathematical Sciences, Durham University, Durham, UK; h KNCV Tuberculosis Foundation, The Hague, Netherlands; i Global Access, IAVI, New York, NY, USA; j Global TB Programme, World Health Organization, Geneva, Switzerland; k Department of Immunization, Vaccines, and Biologicals, World Health Organization, Geneva, Switzerland; l The Initiative for Vaccine Research, World Health Organization, Geneva, Switzerland; m Global Medical Evidence Generation for Influenza Vaccines, Sanofi Pasteur, Singapore

## Abstract

**Background:**

Tuberculosis is a leading infectious cause of death worldwide. Novel vaccines will be required to reach global targets and reverse setbacks resulting from the COVID-19 pandemic. We estimated the impact of novel tuberculosis vaccines in low-income and middle-income countries (LMICs) in several delivery scenarios.

**Methods:**

We calibrated a tuberculosis model to 105 LMICs (accounting for 93% of global incidence). Vaccine scenarios were implemented as the base-case (routine vaccination of those aged 9 years and one-off vaccination for those aged 10 years and older, with country-specific introduction between 2028 and 2047, and 5-year scale-up to target coverage); accelerated scale-up similar to the base-case, but with all countries introducing vaccines in 2025, with instant scale-up; and routine-only (similar to the base-case, but including routine vaccination only). Vaccines were assumed to protect against disease for 10 years, with 50% efficacy.

**Findings:**

The base-case scenario would prevent 44·0 million (95% uncertainty range 37·2–51·6) tuberculosis cases and 5·0 million (4·6–5·4) tuberculosis deaths before 2050, compared with equivalent estimates of cases and deaths that would be predicted to occur before 2050 with no new vaccine introduction (the baseline scenario). The accelerated scale-up scenario would prevent 65·5 million (55·6–76·0) cases and 7·9 million (7·3–8·5) deaths before 2050, relative to baseline. The routine-only scenario would prevent 8·8 million (95% uncertainty range 7·6–10·1) cases and 1·1 million (0·9–1·2) deaths before 2050, relative to baseline.

**Interpretation:**

Our results suggest novel tuberculosis vaccines could have substantial impact, which will vary depending on delivery strategy. Including a one-off vaccination campaign will be crucial for rapid impact. Accelerated introduction—at a pace similar to that seen for COVID-19 vaccines—would increase the number of lives saved before 2050 by around 60%. Investment is required to support vaccine development, manufacturing, prompt introduction, and scale-up.

**Funding:**

WHO (2020/985800-0).

**Translations:**

For the French, Spanish, Italian and Dutch translations of the abstract see Supplementary Materials section.

## Introduction

Tuberculosis is one of the leading causes of infectious disease death worldwide, second only to COVID-19.^1^ The negative impact of COVID-19 on tuberculosis-related health services, such as delays in diagnosis, treatment, and neonatal vaccination has paused and reversed slowly declining trends in mortality.^1^, ^2^

WHO established the End TB Strategy in 2015, with the goal of reducing disease incidence, deaths, and costs worldwide from tuberculosis.^3^ Targets for 2025 include reductions in the absolute number of deaths from tuberculosis by 75% and in incidence by 50%, and targets for 2035 include reductions in the absolute number of deaths by 95% and in incidence by 90%, both compared with 2015 rates.^3^ However, most countries are not on track to achieve these targets.^1^, ^4^

The 2035 End TB targets explicitly assumed the introduction of new tools, including a novel tuberculosis vaccine, by 2025.^3^ WHO has proposed preferred product characteristics for new tuberculosis vaccines,^5^ which were developed through a highly consultative process, including regulators and policy makers from high-burden countries. Although progress has been made, the 2025 target for novel tuberculosis vaccine introduction is unlikely to be achieved.

A phase 2b trial of the M72/AS01_E_ candidate vaccine showed an efficacy of 49·7% (95% CI 2·1–74·2) for preventing disease in adults positive by interferon-gamma release assay after 3 years of follow-up,^6^ and a trial of BCG-revaccination appeared efficacious at preventing sustained infection in a cohort of adolescents negative for interferon-gamma release assay, with an efficacy of 45·4% (6·4–68·1).^7^ Unfortunately, the phase 3 trial of M72/AS01_E_ has not started, and therefore the realistic licensure date, should a positive result be found, might not be for many years. Policy changes on BCG-revaccination in adolescents could happen sooner in settings such as South Africa, but BCG-revaccination has not been tested in individuals positive for tuberculosis infection—a population shown previously to be epidemiologically important for rapid population-level impact.^8^

**Research in context**

**Evidence before this study**

Two systematic reviews in the previous 7 years have highlighted the benefits that novel tuberculosis vaccines could have on reducing the tuberculosis burden globally, and that vaccines are likely to be crucial to achieve elimination. These studies indicate that the impact of novel tuberculosis vaccines will depend on the characteristics of the setting, the vaccine, and the delivery strategy. We searched PubMed on Nov 2, 2022, with no date or language restrictions, to find all studies modelling the impact of vaccines aligned with the WHO preferred product characteristics for new tuberculosis vaccines, using the search terms ((tuberculosis) OR (Mtb)) AND ((vaccine) OR (immunisation)) AND ((WHO) OR (World Health Organization)) AND (preferred product characteristics). We found no studies estimating the potential health impacts of introducing a vaccine with characteristics aligned with the WHO preferred product characteristics in low-income and middle-income countries, and existing literature remains limited in terms of how realistic the modelled vaccine introduction and scale-up scenarios were.

**Added value of this study**

We estimated the potential impact on tuberculosis cases and deaths of vaccines for infants and for adolescents and adults meeting WHO preferred product characteristics in 105 low-income and middle-income countries that accounted for 93% of the global tuberculosis incidence and mortality in 2019. We evaluated more complex and realistic base-case vaccine delivery scenarios than previously modelled by including country-specific introduction years between 2028 and 2047, and scaling up to target vaccine coverage across 5 years upon initial country introduction. The vaccine for infants was assumed to be delivered routinely to neonates, and the vaccine for adolescents and adults was assumed to be introduced routinely to those aged 9 years and as a one-off campaign for those aged 10 years and older. We compared the base-case scenarios to accelerated introduction and scale-up in all countries in 2025, at a speed similar to the pace of COVID-19 vaccine introduction, to estimate the implications of not meeting the End TB strategy target to develop and license a new tuberculosis vaccine by 2025, and scale up quickly. We also compared the base-case scenario for the adolescent and adult vaccine with a less ambitious routine-only introduction (no one-off vaccination for those aged 10 years and older). We grouped countries by WHO region, income group, and tuberculosis burden to identify where the largest impacts of a novel vaccine could be realised and identified the key implications of these findings.

We found novel tuberculosis vaccines meeting the WHO preferred product characteristics could have a substantial impact, which would vary depending on delivery and vaccine characteristics. Inclusion of a vaccination campaign would be crucial for rapid impact. Most lives could be saved by novel vaccine introduction in the WHO South-East Asian region and African region, and higher rate reductions could be seen in low-income countries. Failing to meet the End TB target to develop and license a vaccine for adolescents and adults by 2025, and to quickly scale up roll-out in all countries, could lead to around 3 million more deaths in low-income and middle-income countries, whereas introduction at a pace similar to that achieved with COVID-19 vaccines could increase the number of lives saved before 2050 by around 60%.

**Implications of all the available evidence**

Our new evidence supports investment decisions in vaccine development, manufacturing, and delivery. Millions of additional deaths could be averted with rapid development and licensing of novel tuberculosis vaccines, and preparations should be made for their prompt introduction, including in campaigns, ideally at the pace that COVID-19 vaccines have been introduced.

This situation raises crucial questions for global and country decision makers, including the following: how many lives will be lost if we fail to roll out a novel tuberculosis vaccine by 2025? What is the potential impact if, instead, vaccines are introduced and rolled out following more traditional timelines? And how would these impacts vary by WHO region, income level, and tuberculosis burden?

We aimed to estimate the potential impact of vaccines meeting the WHO specifications^5^ in low-income and middle-income countries (LMICs) across a range of introduction and scale-up scenarios.

## Methods

### Model development and calibration

To estimate the impact of novel tuberculosis vaccines, we developed a compartmental age-stratified dynamic *Mycobacterium tuberculosis* transmission model by adapting features of previous models.^8^, ^9^ We represented tuberculosis natural history with eight compartments, allowing for *M tuberculosis* infection along a spectrum from uninfected to active clinical disease.^10^, ^11^ A detailed description is provided in appendix 5 (pp 3–14).

We incorporated an access-to-care structure to represent systematic differences in tuberculosis burden, social protection, and health-care access by socioeconomic status.^12^ The access-to-care structure contains a high-access-to-care category, representing the top three income quintiles (ie, 60% of the population per country), and a low-access-to-care category, representing the bottom two income quintiles (ie, 40% of the population per country). We assumed no transition between strata, and random mixing (appendix 5 pp 9, 10).

To account for the influences of HIV and antiretroviral therapy (ART) on the risk of infection and progression to disease,^13^, ^14^ we classified countries as having a higher tuberculosis burden due to HIV if more than 15% of tuberculosis cases were among people living with HIV and HIV prevalence was greater than 1% (appendix 5 pp 21, 22). We modelled an HIV structure including categories in which people were classified as HIV-uninfected, HIV-infected and not on ART, and HIV-infected and on ART. The tuberculosis mortality rate and progression risk were increased in both HIV-infected compartments, with greater increases in those not on ART.

For each country, we calibrated a model to epidemiological data using history matching with emulation through the hmer R package,^15^ generating at least 1000 fitted parameter sets per country. Each country model was independently fitted to nine calibration targets in 2019: the country-specific tuberculosis incidence rate (for all ages, those aged 0–14 years, and those 15 years and older, separately), country-specific tuberculosis case notification rate (for all ages, those aged 0–14 years, and those 15 years and older, separately), country-specific tuberculosis mortality rate (for all ages), the global fraction of subclinical tuberculosis among active tuberculosis, and the global risk ratio of active tuberculosis for high-access-to-care relative to low-access-to-care. Models for countries classified as having a high tuberculosis burden due to HIV were fit to four additional country-specific all-age targets in 2019: HIV prevalence, ART coverage, tuberculosis incidence rate in people living with HIV, and tuberculosis mortality rate in people living with HIV. We used the distribution of results produced by these parameter sets to quantify estimation uncertainty.^16^

### Policy scenarios

For each country, a primary baseline scenario with no novel vaccine introduction was simulated, assuming non-vaccine tuberculosis interventions continue at current trends (ie, the status quo, no-new-vaccine baseline scenario). Because reported country-level data include the high coverage of neonatal BCG vaccination and we anticipate no discontinuation across the model time horizon,^17^ neonatal BCG vaccination was not explicitly modelled.

Aligning with the product characteristics described in the WHO preferred product characteristics, we evaluated a novel tuberculosis vaccine for adolescents and adults, and a novel vaccine for neonates and infants.^5^ Vaccines were assumed to prevent disease by reducing progression to subclinical disease and confer a mean protection of 10 years. We assumed the vaccine for adolescents and adults would be efficacious in individuals in any tuberculosis infection state at the time of vaccination (ie, pre-infection and post-infection), with 50% vaccine efficacy. We assumed the vaccine for infants would be efficacious in individuals who were not infected with *M tuberculosis* at the time of vaccination (ie, pre-infection), with 80% efficacy (appendix 5 p 26).

Roll-out of the vaccine for infants was simulated in two scenarios, and, separately, roll-out of the vaccine for adolescents and adults was simulated in three scenarios, with assumptions confirmed through consultation with a range of global tuberculosis vaccine experts involved in research, government, academia, and policy making. The base-case and accelerated scale-up scenarios for the infant vaccine involved routine neonatal vaccination with 85% coverage. The base-case and accelerated scale-up scenarios for the adolescent and adult vaccine involved routine vaccination of those aged 9 years (80% coverage), with a one-time vaccination campaign for all individuals aged 10 years and older (70% coverage). The routine-only scenario (ie, the vaccine for adolescents and adults only) assumed routine vaccination of those aged 9 years (80% coverage). We assumed no differential vaccination by HIV infection or access-to-care status.

We evaluated vaccine delivery scenarios by varying the introduction year and scale-up trends between scenarios and countries (table 1; appendix 5 pp 26–30). In the base-case and routine-only scenarios, based on data from historical vaccine introduction, vaccines were assumed to be introduced in country-specific years and linearly scaled up to coverage targets across 5 years. To estimate introduction years, countries were divided into those that would be procuring with support from Gavi, the Vaccine Alliance and those that would be self-procuring. Factors influencing the timing of vaccine introduction were identified through expert consultation, and included disease burden, previous early adopter status, timelines for Gavi processes, capacity for immunisation, country-specific registration timelines, and commercial prioritisation. A scoring system was applied to each factor, and countries were assigned an aggregate score ranking their introduction position. The number of countries introducing the vaccine per year was informed by pneumococcal vaccine scale-up.^18^ In the accelerated scale-up scenarios, to more resemble the pace of COVID-19 vaccine introduction, all countries introduced vaccines in 2025 with coverage targets reached instantly.

**Table 1:** Characteristics of modelled vaccine delivery scenarios

	**Scenarios for the infant vaccine**		**Scenarios for the adolescent and adult vaccine**		
	Base-case	Accelerated scale-up	Base-case	Accelerated scale-up	Routine-only
Ages targeted	Routine for infants	Routine for infants	Routine for those aged 9 years and a one-time vaccination campaign scaled up across 5 years for those aged 10 years or older	Routine for those aged 9 years and a one-time vaccination campaign in 2025 for those aged 10 years or older	Routine for those aged 9 years
Introduction year	Country-specific	2025	Country-specific	2025	Country-specific
Vaccine roll-out trend	5-year linear scale-up to coverage	Instant scale-up to coverage	5-year linear scale-up to coverage	Instant scale-up to coverage	5-year linear scale-up to coverage
Coverage target (low, medium, and high)	75%, 85%, and 95%	75%, 85%, and 95%	70%, 80%, and 90% for those aged 9 years; 50%, 70%, and 90% for those aged 10 years and older	70%, 80%, and 90% for those aged 9 years; 50%, 70%, and 90% for those aged 10 years and older	70%, 80%, and 90% for those aged 9 years; 50%, 70%, and 90% for those aged 10 years and older

### Health impact indicators

We calculated the cumulative number of tuberculosis cases, treatments, and deaths averted between vaccine introduction and 2050, compared with the number estimated by the baseline scenario between the corresponding years, and we calculated tuberculosis incidence and mortality rate reductions in 2050 for each vaccine scenario compared with the rates estimated by the baseline in 2050. Incidence rates in 2035 for each vaccine scenario were estimated to investigate the feasibility of meeting the 2035 End TB target. Results are presented as the median and 95% uncertainty range for all countries modelled, WHO region, World Bank income group,^19^ and WHO tuberculosis burden level.^1^

### Additional scenario analyses

We conducted scenario analyses to evaluate alternative assumptions regarding vaccine characteristics, delivery, and the baseline scenario. We simulated vaccine scenarios with lifelong protection for both vaccines, as well as scenarios with efficacy of the vaccine for adolescents and adults increased to 75%. For each scenario, low-coverage and high-coverage targets were compared with the medium-coverage targets used for the main analyses. We explored an alternative baseline: the 2025 End TB no-new-vaccine baseline, which assumed strengthening of non-vaccine tuberculosis interventions to meet the 2025 End TB incidence target,^3^ providing an alternative estimate of impact assuming more effective deployment of existing measures (appendix 5 p 25).

### Role of the funding source

The funder was involved in the development of the research question, study design, and provided comments on the manuscript draft, but had no role in the collection, analysis, and interpretation of the data, or writing of the report.

## Results

**Figure 1:**
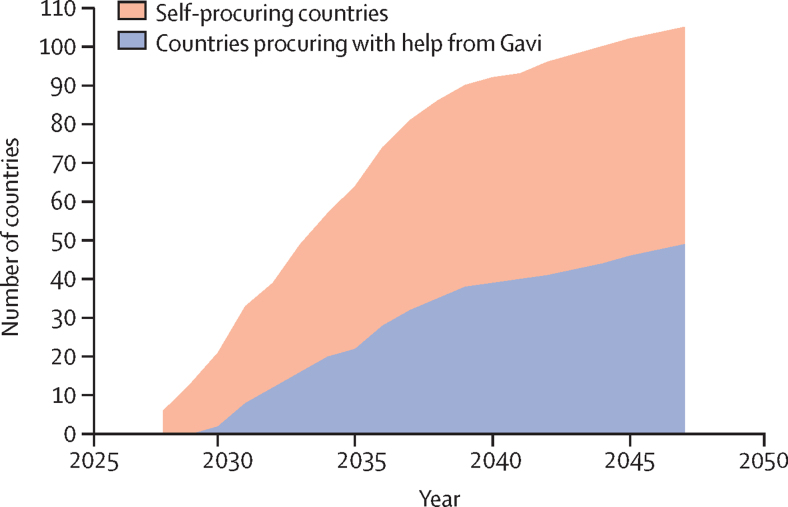
Assumed cumulative number of countries introducing the novel vaccine by year for the base-case and routine-only scenarios Base-case assumes introduction of routine vaccination for those aged 9 years and one-off vaccination for those aged 10 years and older. Routine-only assumes introduction of routine vaccination among those aged 9 years only. The earliest vaccine introduction occurs in 2028 and the latest in 2047. See appendix 5 (pp 26–33) for full details.

Epidemiological and demographic data were available to model 115 of 135 LMICs. We successfully calibrated 105 of 115 countries, accounting for 93% of global tuberculosis cases and deaths in 2019. Calibrated model incidence and mortality rate trends for WHO regions, WHO tuberculosis burden levels, and World Bank income groups are given in appendix 5 (p 41). Country-specific vaccine introduction years (used in base-case and routine-only scenarios) ranged between 2028 and 2047 (appendix 5 pp 35–38). Figure 1 shows the cumulative number of countries introducing the vaccine per year, with 50% of countries introducing the vaccine by 2034.

**Figure 2:**
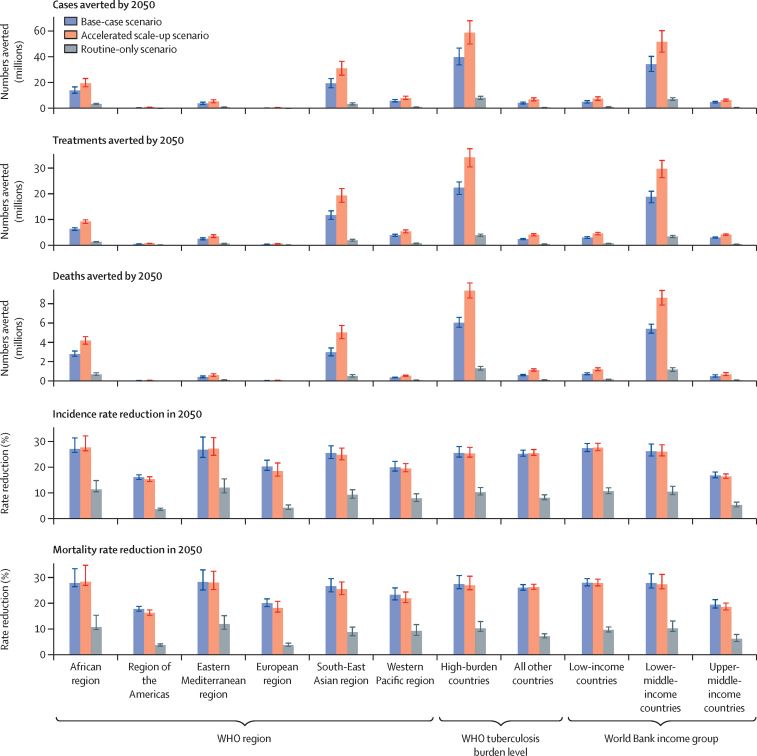
Cumulative cases, treatments, and deaths averted between vaccine introduction and 2050, and incidence and mortality rate reductions in 2050 for the vaccine for adolescents and adults with varying delivery scenarios (50% efficacy vaccine, medium coverage, 10-year duration of protection), by WHO region, WHO tuberculosis burden level, and World Bank income group, expressed relative to a baseline scenario with no new vaccine Cumulative cases, treatments, and deaths averted are calculated for each vaccine scenario compared with the estimated number predicted by 2050 with the status quo no-new-vaccine baseline. Incidence and mortality rate reductions are calculated relative to the incidence and mortality rate predicted in 2050 by the status quo no-new-vaccine baseline. Base-case scenario: routine vaccination of those aged 9 years and a one-off campaign for those aged 10 years and older, introduced in country-specific years between 2028 and 2047 and scaled up over 5 years. Accelerated scale-up scenario: routine vaccination of those aged 9 years and a one-off campaign for those aged 10 years and older, introduced in 2025 and scaled up instantly in all countries. Routine-only scenario: routine vaccination of those aged 9 years, introduced in country-specific years between 2028 and 2047 and scaled up over 5 years.

Our findings suggest that a vaccine for adolescents and adults with 50% efficacy and 10-years of protection in the base-case scenario could avert approximately 44·0 million (95% uncertainty range 37·2–51·6) cases for all countries compared with the status quo no-new-vaccine baseline, including 1·4 million (1·2–1·6) cases of drug-resistant tuberculosis (table 2; appendix p 69). High numbers of cases overall could be averted in the WHO African region and South-East Asian region, which contribute the highest number to the global total, and 34·3 million (28·6–40·3) cases could be averted in lower-middle-income countries (table 2, figure 2). By 2050, 5·0 million (95% uncertainty range 4·6–5·4) deaths could be averted across all countries, including 2·2 million in the South-East Asian region, 2·1 million in the African region, and 4·1 million in lower-middle-income countries (table 2, figure 2). By 2050, 24·9 million (95% uncertainty range 21·9–27·3) treatments could be averted, with 11·7 million (10·1–13·4) averted treatments in the South-East Asian region alone. In the 27 countries categorised by WHO as having a high tuberculosis burden of the 105 countries modelled, 39·8 million (95% uncertainty range 33·7–46·7) cases, 22·6 million (19·9–24·8) treatments, and 4·5 million (4·2–4·9) deaths could be averted by 2050; around ten times higher than those averted in all other countries combined (table 2, figure 2).

**Table 2:** Cumulative cases, treatments, and deaths averted between vaccine introduction and 2050, and incidence and mortality rate reductions in 2050 by WHO region, WHO tuberculosis burden level, and World Bank income group for select vaccine scenarios (all 10-year duration of protection and medium coverage targets)

		**All modelled countries**	**WHO region**						**WHO tuberculosis burden level**		**World Bank income group**		
			African region	Region of the Americas	Eastern Mediterranean region	European region	South-East Asian region	Western Pacific region	High-burden countries	All other countries	Low-income countries	Lower-middle-income countries	Upper-middle-income countries
**Adolescent and adult vaccine**													
Base-case													
	Averted cases before 2050, millions	44·0 (37·2–51·6)	13·9 (11·7–16·7)	0·5 (0·5–0·6)	3·9 (3·1–4·8)	0·3 (0·3–0·4)	19·5 (15·9–23·1)	5·9 (5·0–6·9)	39·8 (33·7–46·7)	4·1 (3·4–4·9)	5·0 (4·1–6·0)	34·3 (28·6–40·3)	4·7 (4·1–5·4)
	Averted deaths before 2050, millions	5·0 (4·6–5·4)	2·1 (1·9–2·3)	0·04 (0·03–0·04)	0·3 (0·2–0·4)	0·03 (0·03–0·03)	2·2 (2·0–2·6)	0·3 (0·2–0·3)	4·5 (4·2–4·9)	0·5 (0·4–0·5)	0·6 (0·5–0·6)	4·1 (3·7–4·4)	0·4 (0·3–0·5)
	Averted treatment before 2050, millions	24·9 (21·9–27·3)	6·3 (5·7–6·8)	0·4 (0·3–0·4)	2·4 (2·0–2·8)	0·2 (0·2–0·3)	11·7 (10·1–13·4)	3·8 (3·3–4·2)	22·6 (19·9–24·8)	2·3 (2·0–2·6)	2·9 (2·5–3·2)	19·0 (16·6–21·2)	2·9 (2·7–3·2)
	Incidence rate reduction in 2050, %	25·4% (23·9–27·7)	27·0% (25·7–31·3)	15·9% (15·2–16·9)	26·7% (23·7–31·6)	20·2% (18·6–22·6)	25·4% (23·3–28·2)	19·8% (18·3–22·2)	25·4% (23·8–27·9)	25·1% (24·1–26·6)	27·3% (26·0–29·1)	26·1% (24·3–28·9)	16·7% (15·8–18·0)
	Mortality rate reduction in 2050, %	27·1% (25·6–30·1)	27·7% (26·3–33·3)	17·7% (16·8–18·7)	28·1% (25·0–32·8)	19·9% (18·6–21·6)	26·5% (24·3–29·4)	23·1% (21·2–25·8)	27·3% (25·5–30·6)	25·9% (25·0–27·1)	27·8% (26·6–29·4)	27·6% (25·8–31·3)	19·4% (18·1–21·3)
Accelerated scale-up													
	Averted cases before 2050, millions	65·5 (55·6–76·0)	19·5 (16·7–23·1)	0·8 (0·7–1·0)	5·4 (4·3–6·7)	0·6 (0·5–0·7)	31·0 (25·8–36·4)	8·1 (6·9–9·5)	58·6 (49·9–67·9)	7·0 (5·8–8·2)	7·5 (6·2–9·0)	51·7 (43·6–60·2)	6·4 (5·6–7·2)
	Averted deaths before 2050, millions	7·9 (7·3–8·5)	3·1 (2·9–3·4)	0·1 (0·1–0·1)	0·5 (0·4–0·6)	0·1 (0·1–0·1)	3·8 (3·3–4·3)	0·4 (0·4–0·4)	7·0 (6·4–7·6)	0·8 (0·8–0·9)	0·9 (0·8–1·0)	6·5 (5·9–7·0)	0·5 (0·4–0·6)
	Averted treatment before 2050, millions	38·6 (34·4–42·3)	9·2 (8·5–9·9)	0·6 (0·5–0·7)	3·4 (2·9–4·0)	0·4 (0·4–0·5)	19·5 (16·8–22·2)	5·3 (4·8–5·9)	34·6 (30·7–37·9)	4·0 (3·5–4·4)	4·5 (4·0–5·0)	30·0 (26·5–33·3)	4·1 (3·7–4·4)
	Incidence rate reduction in 2050, %	25·2% (23·9–27·5)	27·6% (26·3–32·1)	15·2% (14·4–16·2)	27·1% (24·5–31·4)	18·4% (16·4–21·6)	24·7% (22·8–27·3)	19·4% (18·1–21·3)	25·2% (23·8–27·6)	25·3% (24·5–26·8)	27·5% (26·3–29·2)	25·9% (24·3–28·6)	16·3% (15·5–17·3)
	Mortality rate reduction in 2050, %	26·7% (25·2–29·9)	28·2% (26·8–34·6)	16·2% (15·3–17·3)	27·9% (25·2–32·3)	18·1% (16·5–20·7)	25·3% (23·2–28·2)	21·8% (20·2–24·3)	26·8% (25·1–30·4)	26·1% (25·3–27·2)	27·7% (26·6–29·2)	27·2% (25·5–31·0)	18·4% (17·3–20·0)
Routine-only													
	Averted cases before 2050, millions	8·8 (7·6–10·1)	3·5 (3·0–3·9)	0·04 (0·03–0·05)	0·9 (0·7–1·2)	0·02 (0·02–0·03)	3·4 (2·6–4·4)	1·0 (0·8–1·2)	8·1 (7·0–9·3)	0·7 (0·6–0·8)	1·1 (0·9–1·3)	7·2 (6·2–8·3)	0·5 (0·4–0·7)
	Averted deaths before 2050, millions	1·1 (0·9–1·2)	0·5 (0·4–0·6)	0·003 (0·003–0·004)	0·1 (0·1–0·1)	0·002 (0·002–0·003)	0·4 (0·3–0·5)	0·1 (0·0–0·1)	1·0 (0·8–1·1)	0·1 (0·1–0·1)	0·1 (0·1–0·1)	0·9 (0·7–1·0)	0·1 (0·0–0·1)
	Averted treatment before 2050, millions	4·1 (3·7–4·6)	1·2 (1·1–1·4)	0·03 (0·02–0·03)	0·5 (0·4–0·6)	0·01 (0·01–0·02)	1·8 (1·4–2·2)	0·6 (0·5–0·7)	3·8 (3·4–4·2)	0·3 (0·3–0·4)	0·6 (0·5–0·6)	3·3 (2·9–3·8)	0·3 (0·2–0·3)
	Incidence rate reduction in 2050, %	9·9% (9·0–11·6)	11·2% (10·3–14·7)	3·4% (3·1–3·9)	11·9% (9·9–15·3)	4·1% (3·4–5·2)	9·1% (7·8–11·1)	7·7% (6·5–9·5)	10·2% (9·1–12·0)	8·0% (7·3–9·2)	10·5% (9·6–11·9)	10·4% (9·2–12·5)	5·2% (4·4–6·3)
	Mortality rate reduction in 2050, %	9·9% (8·9–12·3)	10·7% (9·7–15·2)	3·7% (3·3–4·2)	11·9% (9·9–15·1)	3·8% (3·3–4·5)	8·7% (7·3–10·7)	9·2% (7·5–11·7)	10·2% (9·1–12·9)	7·2% (6·5–8·1)	9·6% (8·8–10·7)	10·2% (9·0–13·1)	6·2% (5·2–7·8)
**Infant vaccine**													
Base-case													
	Averted cases before 2050, millions	6·7 (5·8–7·7)	2·9 (2·5–3·4)	0·03 (0·02–0·03)	0·8 (0·6–1·1)	0·02 (0·01–0·02)	2·2 (1·6–2·8)	0·8 (0·6–1·0)	6·2 (5·3–7·1)	0·5 (0·4–0·6)	0·9 (0·7–1·1)	5·4 (4·7–6·2)	0·4 (0·3–0·5)
	Averted deaths before 2050, millions	0·9 (0·8–1·0)	0·5 (0·4–0·6)	0·003 (0·002–0·003)	0·1 (0·1–0·1)	0·002 (0·002–0·002)	0·3 (0·2–0·4)	0·1 (0·0–0·1)	0·8 (0·7–1·0)	0·1 (0·1–0·1)	0·1 (0·1–0·1)	0·7 (0·6–0·9)	0·1 (0·0–0·1)
	Averted treatment before 2050, millions	2·7 (2·4–2·9)	0·9 (0·8–0·9)	0·02 (0·01–0·02)	0·4 (0·3–0·5)	0·009 (0·008–0·01)	1·0 (0·8–1·2)	0·4 (0·3–0·5)	2·4 (2·2–2·7)	0·2 (0·2–0·3)	0·4 (0·4–0·5)	2·1 (1·9–2·3)	0·2 (0·1–0·2)
	Incidence rate reduction in 2050, %	8·8% (7·9–10·4)	11·0% (10·0–14·5)	2·7% (2·4–3·1)	12·0% (9·7–15·6)	2·9% (2·5–3·4)	6·9% (5·8–8·6)	7·2% (5·9–9·2)	9·0% (8·1–10·7)	7·1% (6·4–8·2)	9·8% (8·9–11·1)	9·1% (8·1–11·1)	4·7% (3·9–5·9)
	Mortality rate reduction in 2050, %	9·8% (8·7–12·0)	11·3% (10·1–15·7)	3·7% (3·2–4·3)	13·4% (10·5–18·1)	3·3% (2·9–3·9)	7·2% (5·9–9·6)	11·2% (8·5–15·4)	10·1% (8·9–12·5)	7·1% (6·4–8·0)	9·9% (9·0–11·2)	10·0% (8·7–12·5)	6·6% (5·4–8·5)
Accelerated scale-up													
	Averted cases before 2050, millions	16·3 (14·0–18·8)	6·3 (5·4–7·2)	0·1 (0·1–0·1)	1·7 (1·3–2·2)	0·1 (0·1–0·1)	6·6 (5·1–8·6)	1·5 (1·2–1·9)	14·7 (12·6–17·1)	1·6 (1·3–1·9)	2·2 (1·8–2·8)	13·3 (11·4–15·5)	0·8 (0·6–0·9)
	Averted deaths before 2050, millions	2·3 (2·0–2·6)	1·1 (0·9–1·2)	0·007 (0·006–0·008)	0·2 (0·1–0·2)	0·007 (0·006–0·009)	0·9 (0·7–1·2)	0·1 (0·1–0·2)	2·0 (1·8–2·3)	0·2 (0·2–0·3)	0·3 (0·2–0·3)	1·9 (1·6–2·2)	0·1 (0·1–0·1)
	Averted treatment before 2050, millions	7·7 (6·9–8·6)	2·2 (2·0–2·4)	0·04 (0·04–0·05)	0·9 (0·8–1·2)	0·04 (0·04–0·05)	3·6 (2·9–4·3)	0·9 (0·7–1·0)	6·9 (6·2–7·8)	0·7 (0·7–0·8)	1·1 (1·0–1·3)	6·2 (5·5–7·0)	0·4 (0·3–0·4)
	Incidence rate reduction in 2050, %	14·3% (13·0–16·7)	16·7% (15·4–21·6)	4·6% (4·2–5·2)	17·6% (14·5–22·3)	7·5% (6·2–9·8)	12·9% (11·0–15·8)	10·3% (8·7–12·6)	14·4% (13·0–17·0)	13·4% (12·4–14·9)	16·3% (15·1–18·1)	14·9% (13·3–17·9)	6·5% (5·6–7·8)
	Mortality rate reduction in 2050, %	15·9% (14·2–19·3)	17·5% (15·9–24·1)	5·8% (5·2–6·6)	19·2% (15·5–24·7)	7·7% (6·5–9·5)	13·4% (11·2–17·0)	15·0% (12·0–19·3)	16·1% (14·3–19·9)	14·1% (13·1–15·3)	16·8% (15·6–18·5)	16·3% (14·4–20·3)	8·9% (7·5–11·1)

Data are median estimates (95% uncertainty range). Cumulative cases, treatments, and deaths averted are calculated for each vaccine scenario compared with the estimated number predicted by 2050 with the status quo no-new-vaccine baseline. Incidence and mortality rate reductions are calculated relative to the incidence and mortality rate predicted in 2050 relative to the status quo no-new-vaccine baseline. See appendix 5 for all scenarios (pp 55–68).

Introducing the vaccine for adolescents and adults in the base-case scenario was predicted to reduce tuberculosis incidence by 25·4% (23·9–27·7) and deaths by 27·1% (25·6–30·1) in 2050, compared with the status quo no-new-vaccine baseline scenario (table 2). The incidence reduction ranged from 15·9% in the WHO region of the Americas to 27·0% in the African region (table 2, figure 2). Deaths from tuberculosis were estimated to reduce by 17·7% in the region of the Americas to 28·1% in the Eastern Mediterranean region by introducing the adolescent and adult vaccine in the base-case scenario. By income group, the relative impact of the adolescent and adult vaccine was higher in low-income and lower-middle-income countries than in upper-middle-income countries (table 2, figure 2).

In both the base-case and accelerated scale-up scenarios, a lower impact of the infant vaccine (compared with the vaccine for adolescents and adults) was estimated before 2050, including 0·4–0·6 times incidence and mortality rate reductions by 2050 and 0·1–0·3 times the number of cases, treatments, and deaths averted (table 2).

Under the accelerated scale-up scenario, a 50% efficacy of the vaccine for adolescents and adults could prevent 7·9 million (7·3–8·5) deaths—2·9 million more than the base-case—and avert 65·5 million (55·6–76·0) cases and 38·6 million (34·4–42·3) treatments (table 2, figure 2). By contrast, by only routinely vaccinating those aged 9 years (ie, the routine-only scenario), 8·8 million (7·6–10·1) cases, 4·1 million (3·7–4·6) treatments, and 1·1 million (0·9–1·2) deaths would be averted compared with the status quo no-new-vaccine baseline scenario (table 2, figure 2).

Assuming non-vaccine interventions do not improve in the future (ie, the status quo no-new-vaccine baseline), the outcomes of the base-case scenario of introducing the vaccine for adolescents and adults suggest we would reach 34% of the 2035 global target to reduce tuberculosis cases by 90% compared with 2015 levels, and under the accelerated scale-up scenario, we would reach 41% of the target. Assuming the 2025 End TB target of reducing the incidence by 50% compared with 2015 levels is met (ie, the 2025 End TB no-new-vaccine baseline), progress would be increased further, with the base-case and accelerated scale-up scenarios reaching 82% of the target.

Impact results from scenarios with lifelong protection, 75% efficacy, and low-coverage and high-coverage targets are provided in appendix 5 (pp 55–68). Assuming lower coverage targets or the 2025 End TB no-new-vaccine baseline led to reduced vaccine impact compared with vaccines with medium coverage or the status quo no-new-vaccine baseline, and vaccines with higher coverage, 75% efficacy, or lifelong protection led to increased vaccine impact compared with vaccines with medium coverage, 50% efficacy, or 10 years protection.

## Discussion

Our results suggest that novel tuberculosis vaccines could substantially reduce the tuberculosis burden in the coming decades. Relative to the status quo no-new-vaccine baseline, the base-case scenario—in which a tuberculosis vaccine for adolescents and adults with 50% efficacy was introduced during 2028–47—could prevent 44·0 million cases and 5·0 million deaths before 2050, including 2·2 million deaths in the WHO South-East Asian region and 2·1 million deaths in the African region. The more ambitious accelerated scale-up scenario could prevent 65·5 million cases and 7·9 million deaths relative to baseline (which is around 60% more deaths than the base-case scenario). The less ambitious routine-only scenario could prevent 8·8 million cases and 1·1 million deaths relative to baseline (which is around a fifth of the base-case scenario).

Impact estimates for vaccine introduction varied by region in our results. Although incidence and mortality rate reductions achievable by 2050 were similar between high-tuberculosis-burden countries and all other countries, the number of cases, treatments, and deaths averted were around ten times higher than those averted in all other countries, emphasising the need to focus on high-burden countries to maximise health impact. Large numbers of averted cases, treatments, and deaths were predicted in the African region and South-East Asian region, and in lower-middle-income countries, which are arguably populations in the greatest need.

Our modelling suggests that campaigns will be important to expedite health gains from vaccination. The base-case and routine-only scenarios offer a direct comparison of implementing vaccination with and without a campaign for those 10 years and older. The base-case scenario averted up to six times as many cases, deaths, and treatments as the routine-only scenario, supporting the need to include a campaign in any future delivery strategy to maximise health impact.

A new vaccine will be an important tool to accelerate progress towards the 2035 End TB targets. Conservatively assuming non-vaccine interventions do not improve in the future (status quo no-new-vaccine baseline) and roll out from 2028 in line with the pace of historical vaccine uptake, the base-case scenario suggests we could reach around a third of the 2035 global target. More optimistic assumptions, in which the 2025 End TB targets are met before vaccine roll-out (2025 End TB no-new-vaccine baseline), combined with the accelerated scale-up scenario, suggest more than 80% of the global 2035 target could be met.

Two systematic reviews have highlighted potential health impacts of novel tuberculosis vaccines.^20^, ^21^ Our study expands on their findings, and it addresses some identified gaps. We showed that a vaccine for adolescents and adults would have greater and more rapid health impacts than a vaccine for infants before 2050. The largest burden of pulmonary tuberculosis disease is often found in adults;^1^ and in our modelling the vaccine for adolescents and adults was delivered to ages with a higher burden of tuberculosis compared with the vaccine for infants. Because health outcomes are estimated for 2050, the maximum follow-up time between vaccine delivery and impact calculation is 25 years. Therefore, even with the duration of protection increased, the infant vaccine is unlikely to protect those at highest risk of progressing to active disease in most countries during our simulation.

Meeting the End TB target to develop and license a vaccine for adolescents and adults by 2025, and introducing this vaccine at a pace similar to that of COVID-19 vaccines (accelerated scale-up) could avert around 60% more deaths compared with introduction at a historical pace (base-case). The pace of COVID-19 vaccine introduction in LMICs, which was albeit slower than in high-income countries, was much faster than our base-case introduction assumption. As of February, 2023, more than 10% of the population in almost 95% of LMICs (ie, 122 of 129 countries reporting data) have been fully vaccinated since COVID-19 vaccines have been available, showing that faster vaccine introduction in LMICs is possible with high political will and financial resources.^22^ This situation is more similar to our accelerated scale-up scenario, which averted up to 2·9 million more deaths, than our base-case scenario. Although the benefits of rolling out a vaccine from 2028 at a pre-COVID-19 pace are predicted to be large, the increase in deaths shows the consequences of failing to rapidly introduce a vaccine. Unlike COVID-19, tuberculosis is a disease of those on low-incomes, which does not have the associated novelty, nor the same effect on high-income countries. Therefore, tuberculosis vaccines need concerted, sustained policy attention to overcome these barriers.

We successfully calibrated 105 of 135 LMICs, representing 93% of global tuberculosis incidence. Excluding 30 countries will underestimate the number of cases, deaths, and treatments averted, and could bias the generalisability of the relative impact results if the epidemic in the excluded countries is substantially different than those included. Model misspecification and structural uncertainty is possible if country-specific epidemiology does not align with our structure. We used the best available estimates from literature, combined with previous knowledge and expert opinion, to substantiate the prior distributions. Therefore, our results reflect the inherent uncertainty in our knowledge of tuberculosis natural history. For newer discoveries in the field (eg, subclinical disease and self-clearance) data are sparse, and uncertainty is wide, which could bias our vaccine impact estimates. We made assumptions on parameters (eg, assuming the same amount of protection against reinfection in the infection and resolved compartments), which might slightly underestimate vaccine impact. We predicted tuberculosis declining across time, but the projected declines are unlikely to match actual declines, primarily affecting estimates of reaching End TB strategy goals, and numbers averted.

Because there are no new vaccines for tuberculosis, we assumed the characteristics of the modelled vaccines aligned with the recommendations in the WHO preferred product characteristics. Our impact results could be overestimated or underestimated if values for efficacy and duration of protection are lower or higher than the actual characteristics of a new vaccine. We assumed the vaccine for adolescents and adults would be efficacious in all individuals, because testing for tuberculosis infection before vaccination would be costly and logistically difficult. However, most trials have only enrolled individuals who are either positive for interferon-gamma release assay or negative for interferon-gamma release assay.^6^, ^7^ If the vaccine will only be efficacious in those who are positive for interferon-gamma release assay or those who are negative, our results will be overestimates, as shown previously.^8^ We assumed equivalent vaccine efficacy in people living with HIV and those who are HIV-naive, but vaccines are not always as efficacious in individuals who are immunocompromised,^23^, ^24^ which would reduce impact in countries classified as having a high tuberculosis burden associated with HIV.

For vaccine delivery, we attempted to represent a reasonable breadth of possibilities by speaking to experts and evaluating low and high coverage, efficacy, and introduction scenarios. Should there be rapid developments in tuberculosis diagnostics and treatments, or if funding were substantially increased, the impacts could be overestimates or underestimates. Our more ambitious scenario, accelerated scale-up, is less realistic than the base-case scenario, particularly in some LMICs. The scenario assumes a vaccine candidate would be ready for licensure, the supply exists, and that countries are positioned to make an introduction decision resulting in immediate uptake, all within the next 2–3 years, which is unlikely to be attainable by all countries. No specific risk groups were vaccinated in our model; however, initial delivery within countries could be through a targeted approach, which was previously shown to have a large population impact per vaccinated individual.^25^, ^26^, ^27^, ^28^, ^29^ Some countries could initially vaccinate groups at the highest risk of developing disease or who contribute the most to transmission, whereas others could focus on vulnerable ages or those who have had contact with an individual with confirmed tuberculosis disease. Understanding how a new tuberculosis vaccine could be introduced in different settings is an important area for future research.

There are remaining gaps that modelling can address to provide evidence for investing in tuberculosis vaccine development and delivery to inform the Full Value of Vaccine Assessment.^30^ Estimates of the cost-effectiveness, budget effect, and wider benefits of specific tuberculosis vaccine candidates would support research investment decision making. Future modelling research can help to better understand potential vaccine effectiveness considering a variety of factors, such as age, sex, and specific risk groups. We included an access-to-care structure to account for differences in tuberculosis burden and health-care access, which could be used to investigate differential vaccine targeting. To maximise the potential evidence available to countries, creating detailed individual country models to inform vaccine introduction decision making would be beneficial.

Our results suggest that novel tuberculosis vaccines could have a substantial impact on cases of and deaths from tuberculosis, which would vary depending on vaccine and delivery characteristics. Vaccination campaigns will be crucial for rapid impact, and an accelerated introduction that is done at a similar pace to that of COVID-19 vaccine introduction could save around 60% more lives before 2050 than the same vaccine introduced and scaled up across 20 years. The COVID-19 pandemic has shown the advantages that billions of dollars of investment can have on vaccine research and development, and it provides an illustration of what is possible to achieve with novel tuberculosis vaccines. Continued investment in tuberculosis vaccine research is required to strengthen vaccine development, trials, and manufacturing, and to support prompt introduction and scale-up.

## Data sharing

No individual level participant data were used for this modelling study. Epidemiological data used are available from the WHO Global Tuberculosis Report and are summarised in appendix 5 (pp 41–49). Population estimates and projections are available from the UN Department of Economic and Social Affairs World Population Prospects 2019. The analytic code will be available immediately following publication, indefinitely, for anyone who wishes to access the data for any purpose.

## Declaration of interests

SM reports employment by the International AIDS Vaccine Initiative, a non-profit product development partnership supporting the access-oriented development of vaccines for several disease areas, including tuberculosis, and grant funding from WHO. MJ is funded by the Bill & Melinda Gates Foundation, Gavi the Vaccine Alliance, the UK Research Institute, the National Institute for Health Research, the European Commission, and the Wellcome Trust, and reports leadership or fiduciary roles in the board, society, committee, or advocacy groups for WHO and Gavi. RCH reports employment by Sanofi Pasteur, unrelated to tuberculosis and outside the submitted work. NAM received consulting fees from The Global Fund to Fight AIDS, Tuberculosis and Malaria and WHO, and reports funding to their institution from the US Centers for Disease Control and Prevention, the Gates Foundation, the National Institute of Health, and the US Council of State and Territorial Epidemiologists. RGW is funded for other work by the Wellcome Trust (218261/Z/19/Z), the National Institute of Health (1R01AI147321–01), EDCTP (RIA208D-2505B), the UK's Medical Research Council (CCF17–7779 via SET Bloomsbury), the Economic and Social Research Council (ES/P008011/1), the Gates Foundation (OPP1084276, OPP1135288, and INV-001754), and WHO. All other authors declare no competing interests.

## Supplementary Materials

French translation of the abstract

Spanish translation of the abstract

Italian translation of the abstract

Dutch translation of the abstract

## References

[1] WHO (2021). Global tuberculosis report. https://apps.who.int/iris/handle/10665/346387.

[2] McQuaid CF, Vassall A, Cohen T, Fiekert K, White RG (2021). The impact of COVID-19 on TB: a review of the data. Int J Tuberc Lung Dis.

[3] WHO (Aug 16, 2015). The End TB Strategy. https://www.who.int/publications/i/item/WHO-HTM-TB-2015.19.

[4] Stop TB Partnership (2021). New data shows COVID-19 combined with funding shortfalls are devastating efforts to end TB by 2030. https://www.stoptb.org/news/new-data-shows-covid-19-combined-with-funding-shortfalls-are-devastating-efforts-to-end-tb-2030.

[5] WHO (2018). WHO Preferred product characteristics for new tuberculosis vaccines. https://apps.who.int/iris/bitstream/handle/10665/273089/WHO-IVB-18.06-eng.pdf.

[6] Tait DR, Hatherill M, Van Der Meeren O (2019). Final analysis of a trial of M72/AS01_E_ vaccine to prevent tuberculosis. N Engl J Med.

[7] Nemes E, Geldenhuys H, Rozot V (2018). Prevention of *M tuberculosis* infection with H4:IC31 vaccine or BCG revaccination. N Engl J Med.

[8] Harris RC, Sumner T, Knight GM, Zhang H, White RG (2020). Potential impact of tuberculosis vaccines in China, South Africa, and India. Sci Transl Med.

[9] Knight GM, Griffiths UK, Sumner T (2014). Impact and cost-effectiveness of new tuberculosis vaccines in low- and middle-income countries. Proc Natl Acad Sci USA.

[10] Richards AS, Sossen B, Emery JC, et al. The natural history of TB disease—a synthesis of data to quantify progression and regression across the spectrum. *Lancet Glob Health* (in press). 10.1016/S2214-109X(23)00082-7 PMC10126316 36966785

[11] Emery JC, Dodd PJ, Banu S (2022). Estimating the contribution of subclinical tuberculosis disease to transmission—an individual patient data analysis from prevalence surveys. medRxiv.

[12] Siroka A, Law I, Macinko J (2016). The effect of household poverty on tuberculosis. Int J Tuberc Lung Dis.

[13] Kwan CK, Ernst JD (2011). HIV and tuberculosis: a deadly human syndemic. Clin Microbiol Rev.

[14] Suthar AB, Lawn SD, del Amo J (2012). Antiretroviral therapy for prevention of tuberculosis in adults with HIV: a systematic review and meta-analysis. PLoS Med.

[15] Iskauskas A (2022). hmer: history matching and emulation package. https://CRAN.R-project.org/package=hmer.

[16] Briggs AH, Weinstein MC, Fenwick EAL, Karnon J, Sculpher MJ, Paltiel AD (2012). Model parameter estimation and uncertainty analysis: a report of the ISPOR-SMDM Modeling Good Research Practices Task Force Working Group-6. Med Decis Making.

[17] WHO (2022). BCG: immunization coverage estimates by WHO region. https://apps.who.int/gho/data/view.main.81500?lang=en.

[18] UNICEF Data (2022). Immunization. https://data.unicef.org/topic/child-health/immunization/.

[19] The World Bank (2022). World Bank country and lending groups: historical classification by income in XLSX format. https://datahelpdesk.worldbank.org/knowledgebase/articles/906519.

[20] Harris RC, Sumner T, Knight GM, White RG (2016). Systematic review of mathematical models exploring the epidemiological impact of future TB vaccines. Hum Vaccin Immunother.

[21] Weerasuriya CK, Clark RA, White RG, Harris RC (2020). New tuberculosis vaccines: advances in clinical development and modelling. J Intern Med.

[22] Our World in Data (2022). Coronavirus (COVID-19) vaccinations. https://ourworldindata.org/covid-vaccinations.

[23] Nicolini LA, Giacobbe DR, Di Biagio A, Viscoli C (2015). Insights on common vaccinations in HIV-infection: efficacy and safety. J Prev Med Hyg.

[24] Kumarasamy N, Poongulali S, Beulah FE (2018). Long-term safety and immunogenicity of the M72/AS01E candidate tuberculosis vaccine in HIV-positive and -negative Indian adults: results from a phase II randomized controlled trial. Medicine (Baltimore).

[25] Shrestha S, Chihota V, White RG, Grant AD, Churchyard GJ, Dowdy DW (2017). Impact of targeted tuberculosis vaccination among a mining population in South Africa: a model-based study. Am J Epidemiol.

[26] Shrestha S, Chatterjee S, Rao KD, Dowdy DW (2016). Potential impact of spatially targeted adult tuberculosis vaccine in Gujarat, India. J R Soc Interface.

[27] Awad SF, Critchley JA, Abu-Raddad LJ (2019). Epidemiological impact of targeted interventions for people with diabetes mellitus on tuberculosis transmission in India: modelling based predictions. Epidemics.

[28] Harris RC, Sumner T, Knight GM (2019). Age-targeted tuberculosis vaccination in China and implications for vaccine development: a modelling study. Lancet Glob Health.

[29] Weerasuriya CK, Harris RC, McQuaid CF (2021). The epidemiologic impact and cost-effectiveness of new tuberculosis vaccines on multidrug-resistant tuberculosis in India and China. BMC Med.

[30] Hutubessy RCW, Lauer JA, Giersing B (2021). The Full Value of Vaccine Assessments (FVVA): a framework to assess and communicate the value of vaccines for investment and introduction decision making. SSRN.

