# Supplementary material for: The impact of alternative delivery strategies for novel tuberculosis vaccines in low-income and middle-income countries: a modelling study
Source: Lancet Glob Health. 2023 Mar 14;11(4):e546–55. doi: 10.1016/S2214-109X(23)00045-1 (PMC10030455; doi:10.1016/S2214-109X(23)00045-1)
Supplement: French translation of the abstract [file mmc1.pdf]

THE LANCET
Global Health

Supplementary appendix 1

This translation in French was submitted by the authors and we reproduce it as supplied. It has not been peer reviewed. *The Lancet*'s editorial processes have only been applied to the original in English, which should serve as reference for this manuscript.

Cette traduction en français a été proposée par les auteurs et nous l'avons reproduite telle quelle. Elle n'a pas été examinée par des pairs. Les processus éditoriaux du *Lancet* n'ont été appliqués qu'à l'original en anglais et c'est cette version qui doit servir de référence pour ce manuscrit.

Supplement to: Clark RA, Mukandavire C, Portnoy A, et al. The impact of alternative delivery strategies for novel tuberculosis vaccines in low-income and middle-income countries: a modelling study. *Lancet Glob Health* 2023; **11:** e546–55.

Abstract

Contexte

La tuberculose est l'une des principales causes infectieuses de décès dans le monde. De nouveaux vaccins seront nécessaires pour atteindre les objectifs mondiaux et inverser les revers de la pandémie de COVID-19. Nous avons estimé l'impact de nouveaux vaccins contre la tuberculose dans les pays à revenu faible ou moyen (PRFM) selon différents scénarios de distribution.

Méthodes

Nous avons calibré un modèle de tuberculose pour 105 PRFM (93% de l'incidence mondiale). Les scénarios de vaccination ont été mis en œuvre comme suit. *Scénario de Base:* vaccination systématique des enfants de 9 ans et campagne de vaccination unique pour les personnes âgées de plus de 10 ans, avec une introduction spécifique par pays entre 2028 et 2047 et une mise à l'échelle sur 5 ans pour cibler la couverture; *Mise à l'Échelle Accélérée*: comme le *Scénario de Base*, mais tous les pays introduisant le vaccin en 2025 avec une mise à l'échelle instantanée; et *Vaccination Systématique Uniquement*: comme le *Scénario de Base*, mais avec une vaccination systématique uniquement. Les vaccins protégeaient contre la maladie pendant 10 ans, avec une efficacité de 50%.

Résultats

Le *Scénario de Base* a permis d'éviter 44.0 (intervalle d'incertitude à 95 % = 37.2–51.6) millions de cas de tuberculose et 5.0 (4.6–5.4) millions de décès par tuberculose avant 2050, par rapport au nombre estimé de décès prévus en 2050 sans introduction de nouveaux vaccins, dont 2.2 millions dans la région OMS de l'Asie du Sud-Est. Le scénario de *Mise à l'Échelle Accélérée* a permis d'éviter 65.5 (55.6–76.0) millions de cas et 7.9 (7.3–8.5) millions de décès avant 2050. Le scénario *Vaccination Systématique Uniquement* a permis d'éviter 8.8 (7.6–10.1) millions de cas et 1.1 (0.9–1.2) millions de décès avant 2050.

Interprétations

Nos résultats suggèrent que les nouveaux vaccins antituberculeux pourraient avoir un impact substantiel, qui variera en fonction de la stratégie de distribution. L'inclusion d'une campagne sera cruciale pour un impact rapide. Une introduction accélérée plus similaire au rythme des vaccins COVID-19 augmente les vies sauvées avant 2050 d'environ 60%. Des investissements sont nécessaires pour soutenir le développement et la fabrication des vaccins, leur introduction rapide et leur mise à l'échelle.

Financement

OMS (2020/985800-0)
