# Supplementary material for: The impact of alternative delivery strategies for novel tuberculosis vaccines in low-income and middle-income countries: a modelling study
Source: Lancet Glob Health. 2023 Mar 14;11(4):e546–55. doi: 10.1016/S2214-109X(23)00045-1 (PMC10030455; doi:10.1016/S2214-109X(23)00045-1)
Supplement: Spanish translation of the abstract [file mmc2.pdf]

THE LANCET
Global Health

Supplementary appendix 2

This translation in Spanish was submitted by the authors and we reproduce it as supplied. It has not been peer reviewed. *The Lancet*'s editorial processes have only been applied to the original in English, which should serve as reference for this manuscript.

Los autores nos proporcionaron esta traducción al español y la reproducimos tal como nos fue entregada. No la hemos revisado. Los procesos editoriales de *The Lancet* se han aplicado únicamente al original en inglés, que debe servir de referencia para este manuscrito.

Supplement to: Clark RA, Mukandavire C, Portnoy A, et al. The impact of alternative delivery strategies for novel tuberculosis vaccines in low-income and middle-income countries: a modelling study. *Lancet Glob Health* 2023; **11:** e546–55.

Extracto

Antecedentes
La tuberculosis es una de las principales causas infecciosas de muerte en el mundo. Vacunas nuevas son necesarias para alcanzar los objetivos globales y retroceder los daños causados por la pandemia de COVID-19. Nosotros estimamos el impacto de las nuevas vacunas contra la tuberculosis en países de bajos y medios ingresos (PBMI), en escenarios de entrega alternativos.

Métodos
Calibramos un modelo de tuberculosis a 105 PBMI (93% de la incidencia global). Los escenarios de vacunas fueron implementados como *caso base*: vacunación de rutina en los niños de 9 años y una campaña de una sola vacuna para edades de ≥10 años con introducción especifica del país entre el 2028–2047 y aumento de escala durante 5 años hasta lograr la cobertura objetiva; *Aumento acelerado*: como *caso base* pero todos los países introduciendo en el 2025 con aumento instantáneo; y *solo rutin*a: como *caso base* pero solo vacunación de rutina. Las vacunas protegieron contra la enfermedad durante 10 años, con una eficacia del 50%.

Resultados
El escenario de *caso base* evitó 44.0 (rango de incertidumbre del 95 %=37·2–51·6) millones de muertes por tuberculosis antes del 2050, a comparación del número estimado de muertes previsto en el 2050 sin introducción de vacunas nuevas, incluyendo 2.2 millones en la region de Asia sureste de la OMS. El escenario de *aumento acelerado* evitó 65.5 (55.6–76.0) millones de casos y 7.9 (7.3–8.5) millones de muertes antes del 2050. El escenario de *solo rutina* evitó 8.8 (7.6–10.1) millones de casos y 1.1 (0.9–1.2) millones de muertes antes del 2050.

Interpretaciones
Nuestros resultados sugieren qué las novedosas vacunas contra la tuberculosis pueden tener un impacto sustancial, el cual variará según la estrategia de entrega. Incluir una campaña será crucial para el impacto rápido. La introducción acelerada más similar al ritmo de las vacunas contra el COVID-19 aumenta las vidas salvadas antes del 2050 aproximadamente un 60%. La inversión es necesaria para apoyar el desarrollo, fabricación, introducción rápida y la ampliación de vacunas.

Fondos
WHO (2020/985800-0)
