# Supplementary material for: The impact of alternative delivery strategies for novel tuberculosis vaccines in low-income and middle-income countries: a modelling study
Source: Lancet Glob Health. 2023 Mar 14;11(4):e546–55. doi: 10.1016/S2214-109X(23)00045-1 (PMC10030455; doi:10.1016/S2214-109X(23)00045-1)
Supplement: Italian translation of the abstract [file mmc3.pdf]

THE LANCET
Global Health

Supplementary appendix 3

This translation in Italian was submitted by the authors and we reproduce it as supplied. It has not been peer reviewed. *The Lancet*'s editorial processes have only been applied to the original in English, which should serve as reference for this manuscript.

Questa traduzione in italiano è stata proposta dagli autori ed è riportata senza modifiche. Il testo tradotto in italiano non è stato sottoposto al processo di revisione paritaria. Il processo editoriale del *Lancet* è stato esclusivamente adottato per l'originale in inglese, che dovrebbe servire da riferimento per questo articolo.

Supplement to: Clark RA, Mukandavire C, Portnoy A, et al. The impact of alternative delivery strategies for novel tuberculosis vaccines in low-income and middle-income countries: a modelling study. *Lancet Glob Health* 2023; **11:** e546–55.

Abstract

Contesto
La tubercolosi è una delle principali cause di morte da malattia infettiva a livello mondiale. Nuovi vaccini sono necessari per raggiungere gli obiettivi posti dall'Organizzazione Mondiale della Sanità (OMS) e contrastare la battuta d'arresto causata dalla pandemia di COVID-19. Abbiamo stimato l'impatto di nuovi vaccini per la tubercolosi in paesi a basso e medio reddito, esplorando vari scenari per la somministrazione dei vaccini.

Metodi
Abbiamo calibrato un modello di tubercolosi in 105 paesi a basso e medio reddito (93% dell'incidenza globale). Gli scenari vaccinali sono stati implementati come *Caso Base*: vaccinazione di routine dei bambini di 9 anni e una campagna di vaccinazione una tantum per le età 10 con introduzione specifica per paese tra il 2028-2047 e scale-up di 5 anni fino alla copertura target; *Scale-up Accelerato*: come Caso Base, ma tutti i paesi introducono il vaccino nel 2025 con scale-up istantaneo; e *Solo Routine*: come Caso Base, ma solo vaccinazione di routine. Abbiamo assunto che i vaccini proteggessero contro la malattia per 10 anni, con efficacia del 50%.

Risultati
Lo scenario *Caso Base* ha prevenuto 44·0 (intervallo di incertezza del 95% = 37·2–51·6) milioni di casi di tubercolosi e 5·0 (4·6–5·4) milioni di decessi per tubercolosi prima del 2050, rispetto al numero stimato di decessi previsti senza introduzione di nuovi vaccini, di cui 2,2 milioni nella regione del sud-est asiatico dell'OMS. Lo scenario *Scale-up Accelerato* ha prevenuto 65·5 (55·6–76·0) milioni di casi e 7·9 (7·3–8·5) milioni di decessi prima del 2050. Lo scenario *Solo Routine* ha prevenuto 8·8 (7·6–10·1) milioni di casi e 1·1 (0·9–1·2) milioni di morti prima del 2050.

Interpretazione
I nostri risultati suggeriscono che i nuovi vaccini contro la tubercolosi potrebbero avere un impatto sostanziale, che varierà a seconda della strategia di somministrazione. L'inclusione di una campagna di vaccinazione una tantum sarà cruciale per un impatto rapido. Un'introduzione accelerata più simile al ritmo dei vaccini COVID-19 aumenta le vite salvate prima del 2050 di circa il 60%. Sono necessari investimenti per sostenere lo sviluppo, la produzione, la rapida introduzione e lo scale-up del vaccino.

Finanziamenti
WHO (2020/985800-0)
