# Supplementary material for: The impact of alternative delivery strategies for novel tuberculosis vaccines in low-income and middle-income countries: a modelling study
Source: Lancet Glob Health. 2023 Mar 14;11(4):e546–55. doi: 10.1016/S2214-109X(23)00045-1 (PMC10030455; doi:10.1016/S2214-109X(23)00045-1)
Supplement: Dutch translation of the abstract [file mmc4.pdf]

THE LANCET
Global Health

Supplementary appendix 4

This translation in Dutch was submitted by the authors and we reproduce it as supplied. It has not been peer reviewed. *The Lancet*'s editorial processes have only been applied to the original in English, which should serve as reference for this manuscript.

Deze vertaling in het Nederlands werd door de auteurs toegevoegd en we reproduceren de tekst zoals deze werd aangeleverd. Dit Nederlandstalig abstract onderging geen peer review. Het redactioneel proces van The Lancet werd enkel uitgevoerd op de originele Engelse tekst, welke geldt als de referentie voor dit artikel.

Supplement to: Clark RA, Mukandavire C, Portnoy A, et al. The impact of alternative delivery strategies for novel tuberculosis vaccines in low-income and middle-income countries: a modelling study. *Lancet Glob Health* 2023; **11:** e546–55.

Abstract

Achtergrond
Tuberculose is wereldwijd één van de meest dodelijke infectieziekten. Om doelen op het gebied van tuberculosebestrijding te bereiken en om tegenslagen ten gevolge van de COVID-19-pandemie ongedaan te maken zijn nieuwe tuberculose vaccins nodig. We hebben het effect van nieuwe tuberculose vaccins in landen met lage en middeninkomens (LMIC's) doorgerekend bij verschillende scenario's voor het uitrollen van deze vaccins.

Methoden
We hebben een tuberculose model gecalibreerd voor 105 LMICs (met in totaal 93% van de werelwijde incidentie). Vaccin scenarios werden geïmplementeerd als *basis*: routinematige vaccinatie van 9-jarigen en een eenmalige vaccinatiecampagne voor leeftijden vanaf 10 jaar met landspecifieke introductie tussen 2028 en 2047 en opschalen naar de uiteindelijke dekkingsgraad in 5 jaar; *versneld opschalen*: als *basis* maar introductie in 2025 in alle landen met onmiddellijk de uiteindelijke dekkingsgraad; en *routinematig*: als *basis*, maar alleen routinematige vaccinatie. De vaccins worden geacht 10 jaar te beschermen tegen ziekte met een werkzaamheid van 50%.

Bevindingen
Het *basis* scenario voorkomt 44,0 (met een 95% betrouwbaarheidsinterval van 37,2 – 51,6) miljoen tuberculose gevallen en 5,0 (4,6–5,4) miljoen tuberculose doden vóór 2050, vergeleken met het geschatte aantal van de voorspelde sterfgevallen in 2050 zonder introductie van een nieuw vaccin, waaronder 2,2 miljoen in de WHO Zuidoost-Aziatische regio. Het *versneld opschalen* scenario voorkomt 65,5 (55,6–76,0) miljoen tuberculose gevallen en 7,9 (7,3–8,5) miljoen sterfgevallen vóór 2050. Het *routinematig* scenario voorkomt 8,8 (7,6–10,1) miljoen gevallen en 1,1 (0,9–1,2) miljoen doden vóór 2050.

Interpretatie
Onze resultaten suggereren dat nieuwe tuberculose vaccins een belangrijk effect kunnen hebben dat afhankelijk is van de strategie van uitrollen van de vaccins waarbij een vaccinatiecampagne cruciaal is voor een snel effect. Een snelle introductie die in tempo vergelijkbaar is met die van de coronavirus vaccins vergroot het aantal levens dat voor 2050 gered kan worden met ongeveer 60%. Er zijn investeringen nodig voor de ontwikkeling, de productie, het onmiddellijk introduceren en het snel opschalen van het uitrollen van tuberculose vaccins.

Financiering
WHO (2020/985800-0)
