# Supplementary material for: The impact of alternative delivery strategies for novel tuberculosis vaccines in low-income and middle-income countries: a modelling study
Source: Lancet Glob Health. 2023 Mar 14;11(4):e546–55. doi: 10.1016/S2214-109X(23)00045-1 (PMC10030455; doi:10.1016/S2214-109X(23)00045-1)
Supplement: Supplementary appendix 5 [file mmc5.pdf]

THE LANCET
Global Health

Supplementary appendix 5

This appendix formed part of the original submission and has been peer reviewed. We post it as supplied by the authors.

Supplement to: Clark RA, Mukandavire C, Portnoy A, et al. The impact of alternative delivery strategies for novel tuberculosis vaccines in low-income and middle-income countries: a modelling study. *Lancet Glob Health* 2023; **11:** e546–55.

Supplementary Material for *The impact of alternative delivery strategies for novel tuberculosis vaccines in low- and middle-income countries: a modelling study*

Rebecca A. Clark et al.

Table of Contents

SUPPLEMENTAL METHODS: 3

1. Model structure and equations 3

  1.1 Tuberculosis natural history dimension 3

    1.1.1 Tuberculosis natural history structure 3

    1.1.2 Tuberculosis natural history equations 5

    1.1.3 Force of infection equation 6

  1.2 HIV and ART structure 7

    1.2.1 HIV and ART description 7

    1.2.2 HIV and ART diagram 7

    1.2.3 HIV and ART equations 8

    1.2.4 Natural history equations incorporating HIV and ART 8

  1.3 Access to Care Dimension 9

    1.3.1 Access to care description 9

2. Model Parameters and Data Sources 11

  2.1 Model Parameters and Data Sources 11

  2.2 Operationalising Age Varying Parameters 14

3. Tuberculosis treatment 15

  3.1 Tuberculosis treatment initiation 15

  3.2 Tuberculosis treatment outcomes 15

4. Model simulation and calibration methodology 17

  4.1 Model simulation 17

  4.2 Model calibration 17

5. Low- and middle-income countries 19

  5.1 Eligible countries 19

  5.2 Countries excluded from attempting calibration 20

  5.3 Countries classified as having a higher tuberculosis burden due to HIV 21

6. No-New-Vaccine baselines 23

  6.1 *Status Quo No-New-Vaccine* baseline 23

  6.2 *2025 End TB No-New-Vaccine* baseline 25

7. Vaccination 26

  7.1 Vaccine profile 26

  7.2 Vaccine delivery scenarios 26

    7.2.1 Country-specific introduction years 26

    7.2.2 Vaccine coverage targets 29

7.3 Vaccine implementation 31
7.3.1 Vaccine structure 31
7.3.1 Vaccine implementation in the tuberculosis natural history model 33
8. Model outcomes 34
8.1 Epidemiological impact measures 34
8.2 Groupings for reporting model outcomes 34
8.2 Calculating uncertainty 39
SUPPLEMENTAL RESULTS: 40
9. Model Calibration 40
9.1 LMIC calibration 40
9.2 WHO Region and 2021 World Bank Income Group for Calibrated LMICs 40
9.3 *Status Quo No-New-Vaccine* baseline calibration target values 41
9.4 Calibrated *Status-Quo No-New-Vaccine* baseline trends 50
10. Vaccine Health Impact Results 55
10.1 Incidence and mortality rate reductions and cumulative cases, treatments, and deaths averted 55
10.2 Absolute differences in numbers averted between scenarios 65
10.3 Comparing to the 2035 End TB incidence target 67
10.4 Averted cases of drug-resistant tuberculosis 69
References 70

SUPPLEMENTAL METHODS:

1. Model structure and equations

We created a compartmental tuberculosis vaccine model, which includes separate structures to account for key modelling components required. The structures, or "dimensions" we incorporated into the low- and middle-income country (LMIC) modelling are age, tuberculosis natural history, HIV and ART, and access to care.

1.1 Tuberculosis natural history dimension

1.1.1 Tuberculosis natural history structure

The core natural history model is specified in Figure S1.1. Model parameters used in the tuberculosis natural history dimension and their definitions are provided in Table S3.1.

Those with no previous exposure or infection with *Mtb* [Uninfected-Naive (U_N)] could become infected at rate λ_j and progress to an Infection-Fast (I_F) class following initial infection. From Infection-Fast, three possible pathways were possible: (i) Fast progression to Subclinical Disease (D_S), where individuals are infectious with a reduced infectiousness compared to clinical tuberculosis, but display no symptoms of tuberculosis disease;[1] (ii) self-clearance to Uninfected-Cleared (U_C), where individuals are no longer infected with *Mtb* and therefore are not at risk of progression to tuberculosis disease without reinfection;[2] or (iii) continue to remain latently infected with a risk of reactivation and progression to disease, albeit at a lower rate than Infection-Fast, by transitioning to the Infection-Slow (I_S) class. Those in the Infection-Slow class could self-clear to the Uninfected-Cleared class, be reinfected and return to the Infection-Fast class, or reactivate their infection and progress to Subclinical Disease.

Once in the Subclinical Disease class, individuals could naturally cure (*without* treatment) to the Resolved (R) class, or progress to Clinical Disease (D_C), where individuals are infectious and display symptoms of tuberculosis disease. Treatment initiation from Clinical Disease to On-Treatment (T) began in 1960 and increased following a sigmoid curve to 2019, with average treatment duration assumed to be 6 months.[3,4] Treatment completions transitioned to the Resolved class and treatment non-completions returned to Clinical Disease. Deaths occurring on-treatment and in clinical disease counted toward the total number of tuberculosis deaths during the year. Those with clinical disease could also naturally cure to the resolved class. Individuals in the Resolved class could be reinfected or relapse to Subclinical Disease but could *not* enter Infection-Fast or Infection-Slow directly. We assumed that the infection and resolved classes are partially protected against reinfection.[5,6] In those who have self-cleared, we assumed the level of protection against reinfection is half of the protection against reinfection for the infection and resolved classes.

Age was modelled in single years from ages 0 to 79 and aggregated into two categories for ages 80 to 89, and ages 90 to 99. Births and ageing occurred at the beginning of each year.

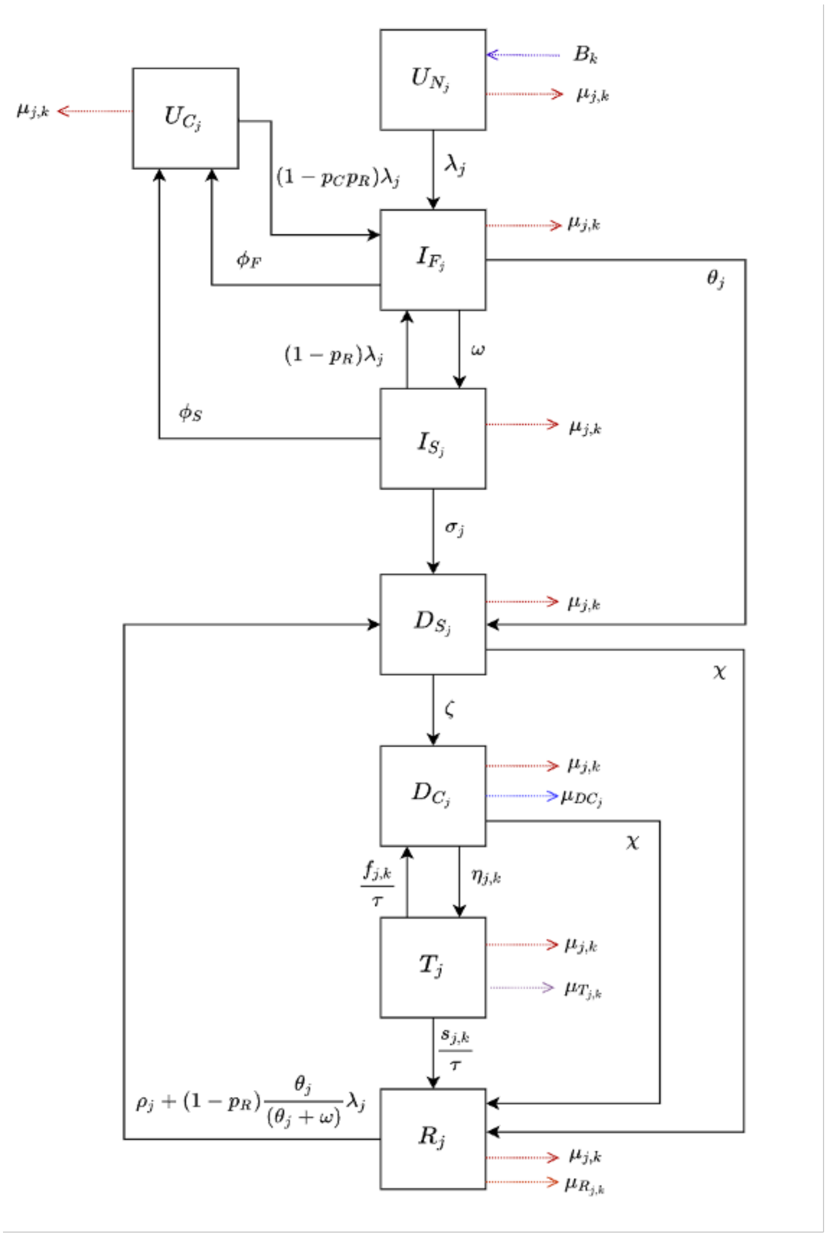

Figure S1.1 Tuberculosis natural history model

Abbreviations: D_C = Clinical Disease; D_S = Subclinical Disease; I_F = Infection-Fast; I_S = Infection-Slow; R = Resolved; T = On-Treatment; U_C = Uninfected-Cleared; U_N = Uninfected-Naive.

Subscript *j* represents parameters that vary by age, and subscript *k* represents parameters that vary over time.

1.1.2 Tuberculosis natural history equations

$$Age\ j = 0 \qquad\qquad Age\ j \neq 0$$

$$\frac{dU_{N_j}}{dt} = B_k - (\lambda_j + \mu_{j,k})U_{N_j} \qquad\qquad \frac{dU_{N_j}}{dt} = -(\lambda_j + \mu_{j,k})U_{N_j}$$

$$\frac{dU_{C_j}}{dt} = \phi_F I_{F_j} + \phi_S I_{S_j} - [(1 - p_C p_R)\lambda_j + \mu_{j,k}]U_{C_j}$$

$$\frac{dI_{F_j}}{dt} = \lambda_j U_{N_j} + (1 - p_C p_R)\lambda_j U_{C_j} + [(1 - p_R)\lambda_j]I_{S_j} - (\phi_F + \omega + \theta_j + \mu_{j,k})I_{F_j}$$

$$\frac{dI_{S_j}}{dt} = \omega I_{F_j} - (\phi_S + \sigma_j + (1 - p_R)\lambda_j + \mu_{j,k})I_{S_j}$$

$$\frac{dD_{S_j}}{dt} = \theta_j I_{F_j} + \sigma_j I_{S_j} + [\rho_j + (1 - p_R)\frac{\theta_j}{\theta_j + \omega}\lambda_j]R_j - (\chi + \zeta + \mu_{j,k})D_{S_j}$$

$$\frac{dD_{C_j}}{dt} = \zeta D_{S_j} + \frac{f_{j,k}}{\tau}T_j - (\chi + \eta_{j,k} + \mu_{DC_j} + \mu_{j,k})D_{C_j}$$

$$\frac{dT_j}{dt} = \eta_{j,k}D_{C_j} - \left(\frac{s_{j,k} + f_{j,k}}{\tau} + \mu_{T_{j,k}} + \mu_{j,k}\right)T_j$$

$$\frac{dR_j}{dt} = \frac{s_{j,k}}{\tau}T_j + (D_{S_j} + D_{C_j})\chi - [\rho_j + (1 - p_R)\frac{\theta_j}{\theta_j + \omega}\lambda_j + \mu_{R_j} + \mu_{j,k}]R_j$$

1.1.3 Force of infection equation

The equation for the age-specific force of infection (λ_j), or the rate at which Uninfected-Naïve individuals acquire *Mtb* infection in the population, is given below. Clinically, infection with *Mtb* can present as pulmonary tuberculosis which impacts the lungs, and extrapulmonary tuberculosis (EPTB) which occurs in sites other than the lungs.[7,8] EPTB is not infectious, and as we are modelling *Mtb* transmission we would want to exclude it. However, the WHO tuberculosis estimates which we calibrated to include both EPTB and pulmonary tuberculosis. Therefore, instead of excluding EPTB from the model, we discounted the force of infection by the proportion of incident cases that are EPTB to account for the fact that they are not infectious and calibrated to the targets that include both EPTB and pulmonary tuberculosis. We also discounted the force of infection to account for the relative reduced infectiousness of subclinical disease compared to clinical disease.

$$\lambda_j = p_T \times \sum_{y=1}^{n_{ygroups}} C[m,y] \times \left(\frac{(1-ep)(TD_{C_y} + rTD_{S_y})}{N_y} \right)$$

where:

$$N_y = \sum_{j=j_{min}}^{j_{max}} U_{N_j} + U_{C_j} + I_{F_j} + I_{S_j} + D_{S_j} + D_{C_j} + T_j + R_j$$

$$TD_{C_y} = \sum_{j=j_{min}}^{j_{max}} D_{C_j} \qquad TD_{S_y} = \sum_{j=j_{min}}^{j_{max}} D_{S_j}$$

Parameter	Definition
j	Age of individual (in years)
y	Age group of contact
$n_{ygroups}$	Number of contact age groups
p_T	Accounting for the probability of transmission per infectious contact
m	Age group of individual
$C[m,y]$	Contact rate between individual of age group m and contact of age group y from Prem et al.[9]
ep	Average proportion of tuberculosis cases that are extrapulmonary
r	Relative infectiousness of subclinical disease compared to clinical disease
TD_{C_y}	Total population in a clinical disease class in age group y
TD_{S_y}	Total population in a subclinical disease class in age group y
N_y	Total population alive in age group y
j_{min}	Minimum age j within age group y
j_{max}	Maximum age j within age group y

1.2 HIV and ART structure

1.2.1 HIV and ART description

In order to account for the influences of human immunodeficiency virus (HIV) and antiretroviral therapy (ART) on the risk of infection with *Mtb* and progression to tuberculosis disease,[10,11] we have implemented an HIV structure composed of 3 compartments: HIV uninfected [HIV_0], people living with HIV (PLHIV) not on ART [HIV_1], and PLHIV on ART [ART]. HIV uninfected individuals acquired HIV and moved from the HIV_0 compartment to the HIV_1 compartment with rate λ_H. Within the HIV_1 compartment, there is a higher risk of tuberculosis progression and an increased tuberculosis mortality rate compared to the HIV_0 compartment. PLHIV are initiated on treatment with ART from HIV_1 following a sigmoid trend which increases over time. The HIV incidence rate decreases over time following a sigmoid trend which depends on ART coverage (as ART coverage increases over time, the HIV incidence rate decreases). The increases in tuberculosis mortality rate and tuberculosis progression are reduced while in ART compared to HIV_1, but still higher than in HIV_0. ART also reduces the HIV mortality rate. Model parameters used in the HIV and ART structure and their definitions are provided in Table S3.1.

1.2.2 HIV and ART diagram

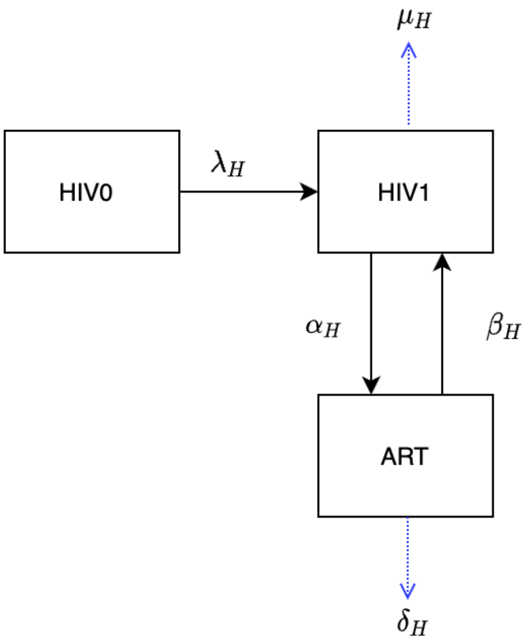

Figure S1.2 HIV and ART structure

1.2.3 HIV and ART equations

$$\frac{d\ \mathrm{HIV}_0}{dt} = -\lambda_H \mathrm{HIV}_0$$

$$\frac{d\ \mathrm{HIV}_1}{dt} = \lambda_H \mathrm{HIV}_0 + \beta_H \mathrm{ART} - (\alpha_H + \mu_H)\mathrm{HIV}_1$$

$$\frac{d\ \mathrm{ART}}{dt} = \alpha_H \mathrm{HIV}_1 - (\beta_H + \delta_H)\mathrm{ART}$$

1.2.4 Natural history equations incorporating HIV and ART

Let $p_{adj} = (1 + th1(\theta_{mul} - 1))$

$Age\ j = 0$

$$\frac{dU_{N_j}}{dt} = B_k - (\lambda_j + \mu_{j,k})U_{N_j}$$

$Age\ j \neq 0$

$$\frac{dU_{N_j}}{dt} = -(\lambda_j + \mu_{j,k})U_{N_j}$$

$$\frac{dU_{C_j}}{dt} = \phi_F I_{F_j} + \phi_S I_{S_j} - [(1 - p_C p_R)\lambda_j + \mu_{j,k}]U_{C_j}$$

$$\frac{dI_{F_j}}{dt} = \lambda_j U_{N_j} + (1 - p_C p_R)\lambda_j U_{C_j} + [(1 - p_R)\lambda_j]I_{S_j} - (\phi_F + \omega + p_{adj}\theta_j + \mu_{j,k})I_{F_j}$$

$$\frac{dI_{S_j}}{dt} = \omega I_{F_j} - (\phi_S + p_{adj}\sigma_j + (1 - p_R)\lambda_j + \mu_{j,k})I_{S_j}$$

$$\frac{dD_{S_j}}{dt} = p_{adj}\theta_j I_{F_j} + p_{adj}\sigma_j I_{S_j} + [p_{adj}\rho_j + (1 - p_R)\frac{p_{adj}\theta_j}{p_{adj}\theta_j + \omega}\lambda_j]R_j - (\chi + \zeta + \mu_{j,k})D_{S_j}$$

$$\frac{dD_{C_j}}{dt} = \zeta D_{S_j} + \frac{f_{j,k}}{\tau}T_j - (\chi + \eta_{j,k} + m_{adj}\mu_{DC_j} + \mu_{j,k})D_{C_j}$$

$$\frac{dT_j}{dt} = \eta_{j,k}D_{C_j} - \left(\frac{s_{j,k} + f_{j,k}}{\tau} + m_{adj}\mu_{T_{j,k}} + \mu_{j,k}\right)T_j$$

$$\frac{dR_j}{dt} = \frac{s_{j,k}}{\tau}T_j + (D_{S_j} + D_{C_j})\chi - [p_{adj}\rho_j + (1 - p_R)\frac{p_{adj}\theta_j}{p_{adj}\theta_j + \omega}\lambda_j + m_{adj}\mu_{R_j} + \mu_{j,k}]R_j$$

1.3 Access to Care Dimension

1.3.1 Access to care description

The access to care dimension is incorporated to allow for the negative correlation between tuberculosis burden and health care access to prevent the overestimation of vaccine impact, as well as to facilitate future analyses of equity implications of vaccine introduction. The access to care dimension contains 2 classes: high-access-to-care, representing the top 3 quintiles (60% of the population) and low-access-to-care, representing the bottom 2 quintiles (40% of the population). We assumed that there was no transition between the high- and low-access-to-care classes, as well as assuming random mixing between the high-access-to-care and low-access-to-care classes.

To constrain relative burden between access-to-care classes, we calibrated the relative tuberculosis prevalence in the high-access-to-care class to the low-access-to-care class in 2019. The calibration target, 0·674, was calculated as a weighted average from eleven studies, with lower and upper bounds (0·575–0·801) representing the 25th and 75th percentiles of the datasets.[12–22] Specifically, a weighted simple linear regression was performed on the log of the prevalence rate ratio of the upper 60% of the population relative to the lower 40% of the population by socioeconomic status (calculations performed by the authors), with weights representing the suspected overlap between potential duplicate observations (0·5 for Philippines and 0·7 for Zambia observations).

Table S1.1 TB prevalence study data

Source	Country	Prevalence rate ratio of upper 60% vs. lower 40% of population by socioeconomic status	Weight
[14]	Bangladesh	0·394	1
[17]	India	0·386	1
[18]	India	0·467	1
[20]	Kenya	0·588	1
[19]	Malawi	0·867	1
[19]	Mongolia	0·716	1
[19]	Myanmar	0·807	1
[19]	Philippines	0·755	0·5
[20]	Philippines	0·608	0·5
[16]	Rwanda	1·081	1
[19]	Rwanda	0·774	1
[12]	South Africa	0·486	1
[21]	South Africa	0·896	1
[19]	Tanzania	0·648	1
[13]	Vietnam	0·701	1
[22]	Vietnam	0·799	1
[19]	Vietnam	0·672	1
[15]	Zambia	0·534	0·7
[19]	Zambia	1·312	0·7
[21]	Zambia	0·728	0·7

To incorporate access to care into our model, we assume that the differences in tuberculosis burden between strata are due to differences in the force of infection, the rate of care-seeking (i.e., tuberculosis treatment initiation), and the rate of tuberculosis progression. We assume relative to the low-access-to-care stratum, the high-access-to-care stratum has a reduced force of infection per contact, an increased rate of treatment initiation, and a reduced rate of tuberculosis progression. Differential burden was implemented by introducing a new parameter p_E, such that $p_E \in [0, 1]$ for the high-access-to-care and $p_E = 0$ for low access-to-care. p_E was included within the model natural history structure as described in Table S1.1. This new parameter was fitted during calibration.

Table S1.2 Implementing the access-to-care parameter

	**Access-to-Care**
**Force of infection**	$p_T \times \sum_{y=1}^{n_{ygroups}} (1 - p_E) \times C[m, y] \times \left(\frac{(1-ep)(TD_{C_y} + rTD_{S_y})}{N_y} \right)$
**Treatment Initiation Rate**	$\frac{\eta_{j,k}}{(1-p_E)}$
**Rate of Tuberculosis Progression**	$(1 - p_E) \times \theta_j$<br>$(1 - p_E) \times \sigma_j$<br>$(1 - p_E) \times \rho_j$

2. Model Parameters and Data Sources

2.1 Model Parameters and Data Sources

Parameters used in the natural history model structure and the HIV and ART model structure are provided in Table S2.1 below, along with their definitions, sources, and information on whether the parameter is fixed or varied (as well as whether they are varied by age or time) during calibration. Further details about how the age varying parameters are implemented are provided in section 2.2. The parameter ranges provided for the tuberculosis natural history parameters are priors fitted during calibration in a Bayesian analysis. We assume that all values within the prior range are equally likely. The prior ranges were pre-specified based on literature review and were reviewed as new data became available.

Table S2.1 Demographic and tuberculosis natural history parameters and definitions

Description	Units	Symbol	Prior	Fixed or Varying During Calibration	Age Varying	Time Varying	Source
Births and deaths (excluding on-treatment mortality)							
Birth rate	Per year	B_k	United Nations World Population Prospects population estimates and projections	Fixed	No	Yes	[23]
Background mortality rate	Per year	$\mu_{j,k}$	Calculated in the model from United Nations population estimates and projections	Fixed	Yes, age specific mortality rates from demographic dataset	Yes	[23]
Mortality rate for clinical tuberculosis disease	Per person per year	μ_{DC_j}	(0–0·178)	Varying	Yes, value for children is greater than value for adults	No	[24]
Mortality rate post-tuberculosis disease	Per person per year	μ_{R_j}	$0.22\mu_{j,k}$	Fixed relationship	Yes, because $\mu_{j,k}$ varies	Yes, because $\mu_{j,k}$ varies	[25]
Natural history							
Force of infection	Per year	λ_j	Fitted	Fixed equation	Yes, age specific contact rates[9]	No	*Calculated*

Probability of transmission per infectious contact	-	p_T	(0–0·0068)	Varying	No	No	*Assumed*
Fraction of total tuberculosis disease that is extrapulmonary	-	ep	Country-specific average of previous 3 years	Fixed	No	No	[26,27]
Infectiousness of subclinical relative to clinical tuberculosis	-	r	0·80	Fixed	No	No	[28]
Rate of self-clearance from I_F to U_C	Per person per year	ϕ_F	0·00000140	Fixed	No	No	[2]
Rate of self-clearance from I_S to U_C	Per person per year	ϕ_S	(0·0254–0·0467)	Varying	No	No	[2]
Rate of fast progression to disease, by age	Per person per year	θ_j	(0·0696–0·111)	Varying	Yes, value for children is less than value for adults	No	[2]
Rate from I_F to I_S	Per person per year	ω	0·5	Fixed	No	No	*Defined*
Rate of reactivation from I_S, by age	Per person per year	σ_j	(0·000135–0·00113)	Varying	Yes, value for children is less than value for adults	No	[2]
Rate of progression from D_S to D_C	Per person per year	ζ	(0–12)	Varying	No	No	*Assumed*
Rate of natural cure from D_C and D_S	Per person per year	χ	(0·1–0·25)	Varying	No	No	[29,30]
Rate of relapse from R, by age	Per person per year	ρ_j	(0·0001–0·07)	Varying	Yes, value for children is less than value for adults	No	[31–33]
Treatment outcome parameters							
Treatment duration	Number of years	τ	0·5	Fixed	No	No	[3,4]
Rate of on-treatment mortality	Per person per year	$\mu_{T_j} = \frac{\kappa_j}{\tau}$	Country-specific	Varying	Yes, value for children greater than value for adults	Yes	[34]

Rate of treatment completion	Per person per year	$\frac{s_j}{\tau}$	Country-specific	Fixed equation	Yes, indirectly scaled by s_{Age}	Yes	[34]
Rate of treatment non-completion	Per person per year	$\frac{f_j}{\tau}$	Country-specific	Fixed equation	Yes, indirectly scaled by s_{Age}	Yes	[34]
Protection parameters							
Protection from reinfection for I_S, I_F, R	-	p_R	(0·6–0·85)	Varying	No	No	[5,6,29,30,35]
Relative protection from reinfection for self-clearance compared to p_R	-	p_C	0·50	Fixed	No	No	*Assumed*
SES parameter	-	p_E	(0–1)	Varying	No	No	*Assumed*
HIV parameters							
HIV incidence rate fitting factor	-	λ_{Hfit}	(0–300)	Varying	No	No	*Fitted*
Rate of ART initiation fitting factor	-	α_{Hfit}	(0–7000)	Varying	No	No	*Fitted*
Rate of ART discontinuation	Per year	β_H	0·074	Fixed	No	No	[36,37]
Mortality rate from HIV not on ART	Per year	μ_H	0·10	Fixed	No	No	[38]
Mortality rate from HIV on ART	Per year	δ_H	0·026	Fixed	No	No	[39]
Relative increase in progression rate for HIV_1	-	θ_{mul}	(3·94–14·45)	Varying	No	No	[40]
Relative reduction in θ_{mul} for HIV and ART compartments	-	$th1$	HIV_0 = 0<br>HIV_1 = 1·00<br>ART = 0·35	Fixed	No	No	[11]
Relative mortality rate adjustment for HIV and ART compartments	-	m_{adj}	HIV_0 = 1·00<br>HIV_1 = 1·50<br>ART = 1·15	Fixed	No	No	[11,24,41,42]

2.2 Operationalising Age Varying Parameters

We assume that aspects of tuberculosis natural history and mortality vary by age. This is implemented by stratifying certain natural history parameters by age and applying age-specific prior ranges and relative constraints during calibration.[43] The following table describes the method used to operationalise the age varying differences in parameters between adults, defined as all ages greater than and equal to 15, and children, defined as all ages less than 15. For the rate per year of reactivation, relapse, and fast progression to tuberculosis disease, we assume that the rate for children is less than that for adults. For the clinical tuberculosis and on-treatment mortality rates, we assume the opposite: the rate for children is higher than that for adults. Age varying for the treatment initiation rate is described in section 3.

Table S2.2 How age varying parameters are operationalised

Parameter	Parameter prior range	Age-specific constraints during calibration	Sample the corresponding age scaling parameter	Adults (θ_{A15})	Children (θ_{A0})
θ_j Rate per year of fast progression	Sample from $(0.0696, 0.111)$	Retain if value for children is less than value for adults (but still within the prior range)	Sample j_1 from $(0, 1)$	Sample θ_{A15} from $(0.0696, 0.111)$	$\max(0.0696, \theta_{A15} \times j_1)$
σ_j Rate per year of reactivation	Sample from $(0.000135, 0.00113)$	Retain if value for children is less than value for adults (but still within the prior range)	Sample j_2 from $(0, 1)$	Sample σ_{A15} from $(0.000135, 0.00113)$	$\max(0.000135, \sigma_{A15} \times j_2)$
ρ_j Rate per year of relapse	Sample from $(0.0001, 0.07)$	Retain if value for children is less than value for adults (but still within the prior range)	Sample j_3 from $(0, 1)$	Sample ρ_{A15} from $(0.0001, 0.07)$	$\max(0.0001, \rho_{A15} \times j_3)$
μ_{DC_j} Clinical tuberculosis mortality rate per year	Sample from $(0, 0.178)$	Retain if value for children is greater than value for adults	Sample S_{Age} from $(0, 1)$	$\mu_{DC_{A0}} \times S_{Age}$	Sample $\mu_{DC_{A0}}$ from $(0, 0.178)$
$\mu_{T_j} = \frac{\kappa_j}{\tau}$ On-treatment mortality rate per year	Sample from $(0, \frac{\kappa_{max}}{\tau})$	Retain if value for children is greater than value for adults	Sample S_{Age} from $(0, 1)$	$\frac{\kappa_{A0}}{\tau} \times S_{Age}$	Sample κ_{A0} from $(0, \kappa_{max})$

3. Tuberculosis treatment

3.1 Tuberculosis treatment initiation

Tuberculosis treatment was assumed to start in 1960, aligned roughly with the discovery and widespread use of rifampicin, and increase following a sigmoid curve (Figure S3.1) to 2019. The treatment initiation rate parameter, η_j, represents the age specific rate of treatment initiation from the clinical disease compartment to the on-treatment compartment. During calibration, a country-specific value for η_j was sampled between 0 and 1. η_j was multiplied by an age scaling parameter for children, j_4, also sampled between 0 and 1, to ensure that the treatment initiation rate in children was less than in adults. This was then multiplied by the value of the sigmoid curve at each year. The treatment initiation rate was calibrated to the country-specific notification rate in 2019 overall and by age reported by the WHO.[26] Due to inconsistencies in the availability of private sector treatment notification data, the contribution of the private sector was not explicitly represented in our model aside from where it had already been incorporated in WHO estimates.

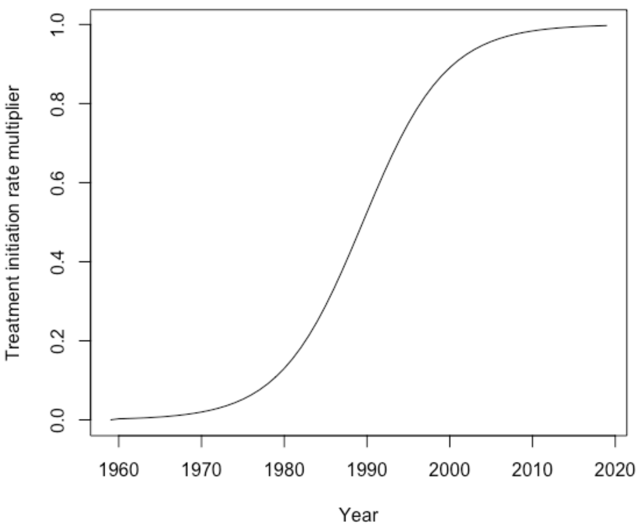

Figure S3.1 Sigmoid curve representing the scale-up in tuberculosis treatment from 1960-2019

3.2 Tuberculosis treatment outcomes

There are three possible exits from the on-treatment compartment: treatment completion, which progresses to the resolved compartment, treatment non-completion, which returns to the clinical disease compartment, and on-treatment mortality, which counts toward tuberculosis mortality. To account for the variability in tuberculosis treatment outcomes and possible underreporting of on-treatment mortality, we used the following country-specific process:

1. For each country separately, the proportion of treatment completions out of the sum of the number of treatment completions and non-completions (previously called "treatment failures") was calculated and averaged over the years of available data from WHO.

Let s_R = Reported number of treatment completions, f_R = Reported number of treatment non-completions

Note: reported number of treatment non-completions included 0·5 × (reported number lost to follow up)

$$\mathrm{SFR} = \frac{s_R}{s_R + f_R}$$

Ex. In India, averaged over 2012–2018, SFR = 0·96. This can be interpreted as of the sum of treatment completions and non-completions, on average, 96% are completions and 4% non-completions.

2. A value for child treatment mortality (κ_{A0}) was sampled between 0 and $(2 \times \text{Average Reported Treatment Mortality})$. The average reported treatment mortality is multiplied by 2 to give an upper bound in the case of unreported data.

   Ex. For India, $\kappa_{A0} \in (0, 0.135)$

3. The age multiplier, S_{Age}, was sampled from $(0, 1)$, and multiplied by κ_{A0} to calculate the adult treatment mortality

$$\kappa_{A15} = \kappa_{A0} \times S_{Age}$$

4. The success and failure rates per year were calculated as in Table S3.1

Table S3.1 Calculating treatment outcome parameter values for adults and children

Parameter	Adults	Children
κ_j On-treatment mortality fraction	$\kappa_{A0} \times S_{Age}$	Sample κ_{A0} from 0 to 2 x Average mortality on-treatment
s_j On-treatment completion fraction	$(1-\kappa_{A15})\mathrm{SFR}$	$(1-\kappa_{A0})\mathrm{SFR}$
f_j On-treatment non-completion fraction	$(1-\kappa_{A15})(1-\mathrm{SFR})$	$(1-\kappa_{A0})(1-\mathrm{SFR})$

5. Each of the parameters in Table S3.1 were divided by τ to obtain the on-treatment mortality rate per year, on-treatment completion rate per year, and on-treatment non-completion rate per year.

4. Model simulation and calibration methodology

4.1 Model simulation

For each country-specific model, we specified a system of ordinary differential equations defining the derivatives with respect to time of a set of state variables, to simulate the country-specific tuberculosis epidemic between 1900 and 2050. We initialised the simulation by distributing the population between the eight tuberculosis natural history states using a fitted parameter representing the proportion of the population uninfected at the start of the simulation. For each year of the simulation (1900–2050), our models are designed to exactly match the age and country specific UN population estimates and projections.[23] Forty percent of the population was assigned to the low access-to-care stratum and the remaining 60% of the population was assigned to the high access-to-care stratum. For countries we classified as having a higher tuberculosis burden due to HIV, the entire population began as HIV uninfected in 1900. As the simulation progresses, the HIV incidence rate is applied and transitions occur to the PLHIV not on ART compartment, and (once ART is introduced in 2000) to the PLHIV on ART compartment.

4.2 Model calibration

Broadly, our modelling approach was as follows:

1. Construct a mechanistic model(s)
2. Calibrate the model(s) by identifying areas of the input parameter space where the output of the mechanistic model was consistent with the historical epidemiologic data
3. Use the calibrated model to simulate and predict future tuberculosis epidemiology and model new vaccines

In the context of this analysis, step 1 was achieved by creating the compartment differential equation model as specified in Section 1. For step 2, we independently calibrated one model for each country by identifying areas of the parameter space that made the output of each country-specific model match the corresponding calibration targets. The model was fitted to calibration targets using history matching with emulation, a relatively new calibration method that allows us to explore high-dimensional parameter spaces efficiently and robustly.[44–47] History matching progresses as a series of iterations, called waves, where implausible areas of the parameter space, i.e., areas that are unable to give a match between the model output (e.g., the predicted incidence rate by the model) and the empirical data (e.g., the incidence rate calibration target from the WHO data), are found and discarded. In order to identify implausible parameter sets, emulators are used. Emulators are statistical approximations of model outputs that are built using a modest number of model runs. Emulators provide an estimate of the value of the model at any parameter set of interest, with the advantage that they are orders of magnitude faster than the model.

History matching with emulation, implemented through the *hmer* package in R,[48] considerably reduced the size of the parameter space to investigate. Rejection sampling was then performed on the reduced space to identify at least 1000 parameter sets that matched all targets for each country.

If countries were unable to find at least 1000 fully fitted parameter sets using history matching with emulation, they were subsequently assessed using an Approximate Bayesian Computation using Markov Chain Monte Carlo method (ABC-MCMC). ABC-MCMC was conducted using the *easyABC* package in R, modified by the Sebastian Funk, Gwenan Knight, and the Tuberculosis Modelling group at LSHTM for adaptive sampling and to accept seeded parameter values.[49,50] We used parameter sets with the maximum number of targets fitted using history matching with emulation as starting seeds for multiple MCMC chains per country, with the ABC-MCMC algorithm continuously adapting using the last 1000 points, a burn in of 1000 samples, and the noise factor set to 0·0001.

Once we had obtained 1000 parameter sets that produced output consistent with the calibration targets, we used those parameter sets with the mechanistic model to simulate the future (step 3) for each country.

5. Low- and middle-income countries

5.1 Eligible countries

To decide which low- and middle-income countries (LMICs) to model on, we used the 135 LMICs indicated on the 2019 World Bank Income Level classifications.[51] The 135 countries were broken down into 29 low-income countries (LICs), 50 lower middle-income countries (LMICs), and 56 upper middle-income countries (UMICs). Distribution by World Bank Income and WHO region are shown below in Table S5.1.

Table S5.1 LMICs by WHO Region and World Bank Income Level 2019

WHO Region	World Bank Income Level 2019			*Total*
	LIC	LMIC	UMIC	
AFR	21	19	5	45
AMR	1	4	20	25
EMR	5	6	5	16
EUR	1	4	15	20
SEAR	1	7	3	11
WPR	0	10	8	18
Total	29	50	56	135

Abbreviations: AFR = WHO African Region, AMR = WHO Region of the Americas, EMR = WHO Eastern Mediterranean Region, EUR = WHO European Region, LIC = low-income countries, LMIC = lower-middle income countries, SEAR = WHO South-East Asian Region, UMIC = upper-middle income countries, WPR = WHO Western Pacific Region

5.2 Countries excluded from attempting calibration

Of the 135 LMIC countries, 20 countries were excluded from attempting calibration due to missing data for calibration. We considered imputing data for countries where it was missing but wanted to keep consistent methods and data sources across the included countries. We do not believe that this omission will have a large impact on our conclusions, given that the excluded countries represented only 3.7% of the total LMIC TB incidence and 5% of the total LMIC TB mortality in 2019. Countries excluded and reasons for exclusion from attempting calibration are provided in Table S5.2 below.

Table S5.2 Countries excluded from attempting calibration and reasons for exclusion

Country	Reason for Exclusion
American Samoa	Missing critical epidemiological data for calibration, no contact matrices available
Belize	No case notification or incidence data for children
Comoros	No case notification data
Democratic Republic of the Congo	No case notification data by age
Republic of the Congo	No population estimates
Democratic People's Republic of Korea	Missing critical epidemiological data for calibration
Djibouti	No case notification data by age
Dominica	Missing critical epidemiological data for calibration, no contact matrices available
Federated States of Micronesia	Missing critical epidemiological data for calibration, no contact matrices available
Grenada	Missing critical epidemiological data for calibration, no contact matrices available
Haiti	Missing 2020 contact matrix
Kiribati	Missing 2020 contact matrix
Kosovo	Missing critical epidemiological data for calibration
Lebanon	Missing 2020 contact matrix
Marshall Islands	Missing critical epidemiological data for calibration, no contact matrices available
Samoa	No case notification or incidence data for children
Somalia	No contact matrices available
St. Lucia	No case notification or incidence data for children
Tuvalu	No contact matrices available
West Bank and Gaza	Missing critical epidemiological data for calibration

5.3 Countries classified as having a higher tuberculosis burden due to HIV

We classified countries as having a higher tuberculosis burden due to HIV if at least 15% of the total tuberculosis cases were in people living with HIV (PLHIV) and if the HIV prevalence in the country was greater than 1%, and included a separate stratum to dynamically model the tuberculosis-HIV co-epidemic. Our definition resulted in 23 of the 115 worth running countries classified as having a higher tuberculosis burden due to HIV (Table S5.3).

Table S5.3 HIV prevalence and proportion of total tuberculosis cases that were in PLHIV for countries classified as having a higher tuberculosis burden due to HIV

Country	HIV Prevalence in 2019 (%)[23,52]	Proportion of total tuberculosis cases that were PLHIV in 2019 (%)[26]
Botswana	16·5 (14·8–17·8)	48·6 (39·0–59·0)
Central African Republic	2·1 (1·8–2·7)	25·4 (20·0–30·0)
Côte d'Ivoire	1·7 (1·4–1·9)	17·5 (15·0–21·0)
Cameroon	2·0 (1·7–2·2)	26·8 (21·0–32·0)
Gabon	2·3 (1·7–3·0)	32·8 (19·3–44·0)
Ghana	1·1 (0·8–1·5)	20·8 (17·0–26·0)
The Gambia	1·2 (0·9–1·5)	17·7 (14·0–22·0)
Guinea-Bissau	2·1 (1·8–2.4)	31·3 (25·0–38·0)
Equatorial Guinea	4·8 (3·5–6·5)	26·5 (25·8–26·8)
Guyana	1·1 (1·0–1.2)	19·0 (18·0–20·0)
Kenya	2·9 (2·5–3·2)	26·2 (21·0–32·0)
Lesotho	16·0 (15·1–16·9)	61·6 (50·0–74·0)
Mozambique	7·2 (5·9–9·2)	33·8 (27·0–41·0)
Malawi	5·9 (5·2–5·9)	46·6 (46·2–56·0)
Namibia	8·4 (7·6–8·8)	32·5 (32·5–39·0)
Rwanda	1·8 (1·6–2·0)	21·1 (17·0–25·0)
Eswatini	17·4 (16·5–19·2)	60·1 (56·6–62·4)
Togo	1·5 (1·2–1·7)	16·2 (13·0–20·0)
Tanzania	2·9 (2·6–3·1)	23·6 (19·0–29·0)

Uganda	3·4<br>(3·2–3·6)	39·0<br>(31·0–47·0)
South Africa	12·8<br>(11·8–13·7)	58·0<br>(46·0–70·0)
Zambia	6·7<br>(5·4–7·3)	46·2<br>(35·0–56·0)
Zimbabwe	9·6<br>(8·2–10·9)	59·8<br>(48·0–72·0)

6. No-New-Vaccine baselines

6.1 *Status Quo No-New-Vaccine* baseline

The primary no-new-vaccine simulated was the "*Status Quo No-New-Vaccine*" baseline, which assumed non-vaccine tuberculosis interventions continue at current levels into the future. As reported country-level data includes the high coverage levels of neonatal BCG vaccination, this was not explicitly modelled. We assumed that BCG vaccination would not be discontinued over the model time horizon.

All countries were fitted to nine calibration targets in 2019:

- The country-specific tuberculosis incidence rate per 100,000 population (all ages, ages 0–14, and ages 15+)
- The country-specific tuberculosis case notification rate per 100,000 population (all ages, ages 0–14, and ages 15+)
- The country-specific tuberculosis mortality rate per 100,000 population (all ages)
- The global estimate of the ratio of the prevalence of subclinical tuberculosis with the prevalence of active tuberculosis (subclinical + clinical tuberculosis)[1]
- The global estimate of the ratio of the prevalence of active tuberculosis in the high access-to-care class relative to low access-to-care[12–22]

In addition to the targets above, countries classified as having a higher tuberculosis burden due to HIV were fitted to four HIV specific calibration targets for all ages in 2019:

- HIV prevalence (%)
- ART coverage (%)
- Tuberculosis incidence rate in PLHIV per 100,000 population (all ages)
- Tuberculosis mortality rate in PLHIV per 100,000 population (all ages)

For the country-specific calibration targets, Table S6.1 indicates where the data to inform the targets was sourced and which variables were used.

For the data obtained from the WHO, we subset the data sets to the 135 LMICs and excluded countries if they were missing key variables indicated in Table S6.1 used to calculate calibration targets. For the data obtained from the UN population division and Prem et al.,[9] we subset the data sets to the 135 LMICs and excluded countries if they were missing population estimates and projections or contact matrices. Countries excluded and reasons for exclusion from further analysis are detailed in Table S5.2.

Data manipulation to create incidence and case notification rate calibration targets

The age-specific and case notification data required further manipulation to a usable form for calibration targets. The best estimate, as well as low and high estimates for the number of incident cases by age were transformed to rates per 100,000 population. We aggregated the UN population data in 2019 into the required age groups (children = 0–14, adults 15–99), and calculated the targets as follows:

$$\frac{\text{Number of incident tuberculosis cases in age group}}{\text{Total population of age group}} \times 100,000$$

Only one estimate of the number of case notifications per age group was provided. Once we calculated the estimate of the age specific case notification rate following the same method as used for the number of incident tuberculosis cases in the age group, we manually added 20% uncertainty bounds.

Data manipulation for simulation age groups

We simulated the model with 82 age groups (single ages from 0–79, and then aggregated groups for ages 80–89 and 90–99). We aggregated age specific data from the UN population estimates and projection by year to create the two age groups used for ages 80–99.

Table S6.1 Sources and variables used for calculations of country-specific calibration targets

Calibration target description	Source	Variables used
**All countries**		
The country-specific tuberculosis incidence rate per 100,000 population (all ages, ages 0–14, and ages 15+)	WHO TB incidence estimates disaggregated by age group, sex and risk factor (Retrieved 28 October 2020)[53]<br><br>UN population estimates and projections[23]	best<br>lo<br>hi
The country-specific tuberculosis case notification rate per 100,000 population (all ages, ages 0–14, and ages 15+)	Case Notifications (Retrieved 28 October 2020)[27]<br>- Number of notified TB cases reported by countries and territories to the WHO<br><br>UN population estimates and projections[23]<br><br>The Case Notifications dataset downloaded from the WHO website provides the number of estimated case notifications. We added 20% uncertainty on the calculated target to create upper and lower bounds for calibration.	newrel_f014<br>newrel_m014<br>newrel_f15plus<br>newrel_m15plus<br>c_newinc
The country-specific tuberculosis mortality rate per 100,000 population (all ages)	WHO TB burden estimates (Retrieved 28 October 2020)[26]<br>- Estimates generated by WHO	e_mort_100k<br>e_mort_100k_lo<br>e_mort_100k_hi
**Countries classified as having a higher tuberculosis burden due to HIV**		
HIV prevalence (%)	HIV estimates with uncertainty bounds 1990-Present (Retrieved 28 October 2020)[52]<br>- Sheet 2: HIV estimates – by Area<br><br>UN population estimates and projections[23]	Estimated adults and children living with HIV<br><br>Estimate, Low, High
ART coverage (%)	HIV estimates with uncertainty bounds 1990-Present (Retrieved 28 October 2020)[52]<br>- Sheet 4: HIV Test & Treat – by Area	Among people who know their HIV status, the percent on ART (All Ages)<br><br>Estimate, Low, High
Tuberculosis incidence rate in PLHIV per 100,000 population (all ages)	WHO TB burden estimates (Retrieved 28 October 2020)[26]<br>- Estimates generated by WHO	e_inc_tbhiv_100k<br>e_inc_tbhiv_100k_lo<br>e_inc_tbhiv_100k_hi
Tuberculosis mortality rate in PLHIV per 100,000 population (all ages)	WHO TB burden estimates (Retrieved 28 October 2020)[26]<br>- Estimates generated by WHO	e_mort_tbhiv_100k<br>e_mort_tbhiv_100k_lo<br>e_mort_tbhiv_100k_hi

6.2 *2025 End TB No-New-Vaccine* baseline

To provide conservative estimates on absolute vaccine impact, we simulated an alternative *No-New-Vaccine* baseline assuming scale-up between 2019 and 2025 in order to meet the End TB incidence target in 2025 of a reduction in 50% of the tuberculosis incidence rate in 2015 by 2025 for all ages.[54] To implement this, an additional parameter, p_F (equal to 1 up to and including 2019 and sampled between 0 and 1 afterwards), was included in the model as a contact rate multiplier within the force of infection equation, and as a multiplier on the progression to disease flows. Using the fully fitted parameter sets for each country from the *Status Quo No-New-Vaccine baseline*, we then varied p_F during calibration to hit the country-specific target of a reduction in 50% of the tuberculosis incidence rate in 2015 in 2025.

7. Vaccination

7.1 Vaccine profile

The vaccine profile for an adult/adolescent vaccine and infant vaccine were based on the WHO Preferred Product Characteristics for New Tuberculosis vaccines,[55] and are outlined in Table S7.1 below.

Table S7.1 WHO Preferred Product Characteristics for New Tuberculosis Vaccines

Vaccine	Host infection status at time of vaccination required for efficacy	Effect type	Vaccine efficacy	Duration of protection
Adolescent / Adult	Pre- and post-infection	Prevention of disease	50%	Lifelong
				10 years
Infant	Pre-infection	Prevention of disease	80%	Lifelong
				10 years

Vaccine efficacy was assumed to be the same in both PLHIV and HIV-naïve recipients in countries classified as having a higher tuberculosis burden due to HIV, and in both younger age groups and older adults. The vaccine was assumed to have the same impact on preventing drug-susceptible and drug-resistant tuberculosis as specified in the WHO PPCs,[55] and as we were modelling a prevention of disease vaccine, there was no direct impact on *Mtb* transmission or the force of infection.

7.2 Vaccine delivery scenarios

The infant vaccine was implemented in two scenarios, and, separately, the adolescent/adult vaccine was implemented in three scenarios. The *Basecase* and *Accelerated Scale-up* scenarios included routine neonatal vaccination for the infant vaccine (85% coverage), and routine vaccination of 9-year-olds (80% coverage) with a one-time vaccination campaign for ages ten and older (70% coverage) for the adolescent/adult vaccine. The *Routine Only* scenario (adolescent/adult vaccine only) was introduced through routine 9-year-old vaccination only (i.e., no campaign). Specifics of the infant and adolescent/adult vaccine scenarios are provided in Table S7.5.

7.2.1 Country-specific introduction years

In the *Basecase* and *Routine Only* scenarios, vaccines were introduced in country-specific introduction years between 2028 and 2047. To calculate the specific year of introduction, countries were divided into two general categories: those procuring with support from Gavi, the Vaccine Alliance, and those self-procuring. Determination of country status was based on eligibility information posted on Gavi's website.[56] Countries transitioning from Gavi support are able to benefit from Gavi pricing and incremental financing for a period of 5-10 years. For countries that have already initiated the period of transition by 2019, this window will have largely ended by the time of tuberculosis vaccine availability through Gavi. As such, these countries were categorised as self-procuring

countries. Countries that have not yet commenced transition, including India and Nigeria, were categorised as Gavi supported countries, given the long grace period post-commencement of transition. For more information, please see Gavi, https://www.gavi.org/types-support/sustainability/transition (last accessed 2 November 2022).

Through a consultative process with experts from WHO, Gavi, PATH, PDVAC, CHAI, and industry partners, factors influencing likelihood of being an early or late adopter were identified for both Gavi and self-procuring countries. Identified factors include disease burden, immunization capacity, and early adopter status. Country-specific registration timelines and commercial prioritization were also deemed important determinants of introduction timing for self-procuring countries.

Additional factors for Gavi countries: For countries procuring through Gavi, timelines for introduction are also influenced by Gavi processes. Prior to offering a new vaccine, Gavi requires that products be licensed, included in Gavi's Vaccine Investment Strategy, reviewed by SAGE, recommended in a WHO position paper, WHO prequalified, and approved for procurement by Gavi (Table S7.2). In addition, time for country application processing, contracting, and delivery must be factored. Through consultations, it was determined that a baseline time of roughly two years post licensure would be needed for Gavi processes prior to first country introduction, assuming several steps advance in parallel.

Table S7.2 Timelines for Gavi processes post licensure

	Cumulative additional time (years)		
**Activities post licensure**	**Low End**	**High End**	**Average**
WHO PQ	0·25	1·00	0·63
SAGE Policy Review & WHO Position Paper	0·25	0·50	0·38
Gavi Decision	0·25	0·50	0·38
National review & Country applications	0·25	0·75	0·50
Contracting & delivery	0·25	0·50	0·38
**Years**	**1·25**	**3·25**	**2·25**

Weight of criteria, indicators, and scoring: Differential weight was assigned to criteria based on their relative impact on the order of country adoption. This weight varied for self-procuring and Gavi countries (Table S7.3).

Table S7.3 Weight of criteria influencing order of country adoption

Criteria	Self-procuring countries	Gavi countries
Disease burden	30%	45%
Immunization capacity	15%	30%
Early adopter/leader	15%	25%
Lack of regulatory barriers	15%	NA
Commercial prioritization	25%	NA

The following indicators were used to measure each of the variables identified in Table S7.3.

Table S7.4 Indicators of criteria influencing order of country adoption

Criteria	Indicator
**Disease burden**	Tuberculosis incidence
**Immunization capacity**	% receiving 3 doses DPT3 among infants 1 years of age (The percent of infants receiving 3 doses DPT3 is commonly used as a proxy for assessing immunization infrastructure)
**Lack of regulatory barriers**	Signatories to WHO PQ or SRA collaborative registration scheme<br>Lack of requirements for additional local clinical trial data
**Early adopter/leader**	Time to policy adoption of universal Xpert MTB/RIF screening for presumed tuberculosis cases<br>Time to adoption of HPV
**Commercial prioritization**	
Ability to finance vaccines	GDP per capita
Political will to address tuberculosis	Spending per tuberculosis case
Market potential	Population

To standardise across these varied metrics, a point value ranging from 1–5 per criteria was assigned, with a score of 1 correlating with an earlier adopter and score of 5 correlating with a later adopter.

*Continuous variable*s such as disease burden or population were divided into quintiles. Those in the highest quintile were assigned a score of 1, those in the second highest quintile received a score of 2, and so forth. *Categorical variables* such as registration or early adopter status were scored based on whether countries met fixed criteria. For instance, countries that are signatories of WHO PQ or SRA collaborative registration schemes were assigned a score of 1. Those that are not signatories and have requirements for additional clinical trial data in local populations received a score of 5.

Scores were then weighted as reflected in Table S7.3 and aggregated into a composite score to determine countries' relative position in the queue of introductions. Composite scores for each of the 135 countries are provided in the SupplementaryMaterial_CountryTimelines.xls.

Assumptions for the pace of introduction—i.e., how many countries per year would introduce the product and what the scale up curve might look like— was informed with data from pneumococcal vaccine (PCV) scale-up.[57] The percent of countries adopting each year (year 1 to year 12) for PCV was calculated. These annual percentages were then applied to tuberculosis vaccine scale up (based on a total n=135 countries: 78 self-procuring countries and 57 Gavi countries). The first year of tuberculosis vaccine scale up was estimated to be 2028, with Gavi countries following a similar scale up trajectory but delayed by two years due to required Gavi lead time for processing new vaccines (Table S7.2). Because PCV data is only available for 12 years, data was extrapolated for years 13 to 20 of tuberculosis vaccine roll out at a steady state. Country introduction timelines were adjusted—where applicable—to group countries with the same composite score in the same year of adoption. The cumulative number of countries introducing the vaccine by year is shown in Figure S7.1, and the country-specific introduction year for each country is in Table S8.1.

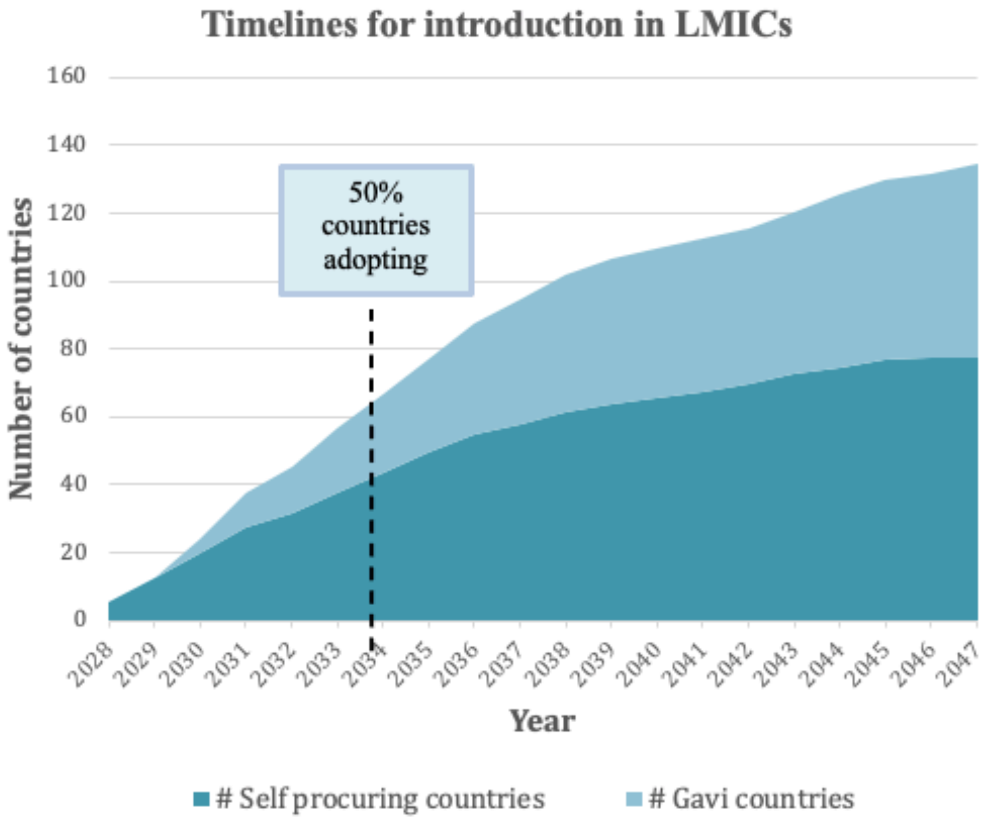

Figure S7.1 **Assumed cumulative number of countries introducing a novel vaccine per year**

7.2.2 Vaccine coverage targets

For each vaccine implementation scenario, low, medium, and high coverage targets for 5 years post-introduction were evaluated. The medium coverage target for the routine infant vaccination was 85%, based on the 2019 DTP3 (diphtheria, tetanus toxoid, and pertussis) average coverage level according to the WHO and UNICEF estimates of national immunisation coverage, with 10% uncertainty (low coverage = 75%, high coverage = 95%).[57] Routine adolescent vaccination assumed a medium coverage target of 80% aligning with HPV coverage in South Africa combined with aggregated secondary school enrolment in China and India as assumed in Harris 2020,[58] also with 10% uncertainty targets (low coverage = 70%, high coverage = 90%). The medium coverage target for the adolescent/adult campaign was 70% aligning with the lower bound of the MenAfriVac campaigns in sub-Saharan Africa as assumed in Harris 2020,[58] with a wider uncertainty of 20% (low coverage = 50%, high coverage = 90%). In the *Accelerated Scale-up* implementation, the 5-year coverage targets are achieved instantly in year 1, while in the *Basecase* and *Routine Only* implementations, the scale-up to coverage occurs linearly over 5 years.

Table S7.5 Vaccine scenarios for the infant and adolescent/adult vaccines

Characteristics	Infant Vaccine Scenarios		Adolescent/Adult Vaccine Scenarios		
	Basecase	***Accelerated Scale-up***	***Basecase***	***Accelerated Scale-up***	***Routine Only***
**Ages Targeted**	*Neonatal:* Routine	*Neonatal:* Routine	*Age 9:* Routine<br><br>*Ages 10+:* One-time vaccination campaign over 5 years	*Age 9:* Routine<br><br>*Ages 10+:* One-time vaccination campaign in 2025	*Age 9:* Routine
**Introduction Year**	Country-specific	2025	Country-specific	2025	Country-specific
**Vaccine Rollout Trend**	5-year linear scale-up to coverage	Instant scale-up to coverage	5-year linear scale-up to coverage	Instant scale-up to coverage	5-year linear scale-up to coverage
**Target Coverage *(Low/Med/High)***	75% / 85% / 95%		Age 9: 70% / 80% / 90%<br>Ages 10+: 50% / 70% / 90%		

7.3 Vaccine implementation

7.3.1 Vaccine structure

To simplify accounting for the number of vaccinees and vaccinations in the model, we included vaccines through an additional "vaccine structure" with three compartments (Figure S7.2) with the influences of vaccines on tuberculosis natural history parameters occurring separately in the natural history structure (Figure S7.3). Each compartment in the vaccine structure is replicated for all tuberculosis natural history compartments, access-to-care strata, HIV statuses, and ages.

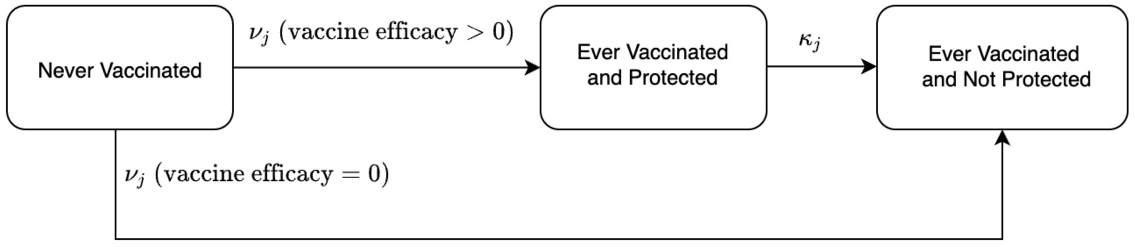

Figure S7.2 Vaccine structure

Before vaccination, all individuals in the model begin in the *Never Vaccinated* compartment, with no vaccine protection. Upon vaccination, individuals either transition to the *Ever Vaccinated and Protected* compartment (with vaccine protection) or *Ever Vaccinated and Not Protected* compartment (with no vaccine protection), depending on vaccine specific host infection status at time of vaccination required for vaccine to be efficacious and their infection status at the time of vaccination, summarised in Table S7.6.

In this work, we modelled the infant vaccine as a pre-infection (PRI) vaccine, meaning the individual must be uninfected at the time of vaccination for the vaccine to be efficacious. We modelled the adolescent/adult vaccine as a pre- and post-infection (PPI) vaccine, which means that it will be efficacious in any infection status at time of vaccination aside from active disease. We assumed that the effect of disease on the immune response is likely to be substantially larger than any additional benefit from the vaccine, and therefore would not be efficacious in those compartments. For example, the Phase 2b M72/AS01$_E$ trial saw a small number of cases in each arm within the first 6 months after ruling out those who were XPERT positive on the day they were tested. Assuming those cases were individuals who were subclinical but not XPERT positive on the day they were tested, the vaccine had no impact on their disease progression.[59]

The arrow directly from *Never Vaccinated* to *Ever Vaccinated and Not Protected* was included to account for individuals who may be accidentally administered a vaccine which would not be efficacious (i.e., vaccine efficacy is zero) given their infection status at the time of vaccination. As individuals with subclinical disease present with no symptoms, it is possible that they may be accidentally vaccinated, as seen in the Phase 2b M72/AS01$_E$ trial. Similarly, with a PRI vaccine, if no pre-vaccination testing is available, it is possible that individuals who are not uninfected may be vaccinated. By including the flow directly to *Ever Vaccinated and Not Protected*, we could easily identify and track these individuals, and ensure they received no protection from the vaccine in the model.

Table S7.6 Transitions within the vaccine structure following vaccination based on natural history state and host infection status at time of vaccination required for vaccine to be efficacious

	**Host infection status at the time of vaccination required for vaccine to be efficacious**	
**Natural History State (Infection Status) at time of vaccination**	**Pre-infection vaccine** (i.e., the infant vaccine)	**Pre- and post-infection vaccine** (i.e., the adolescent/adult vaccine)
Uninfected – Naïve	*Ever Vaccinated and Protected*	*Ever Vaccinated and Protected*
Uninfected – Cleared	*Ever Vaccinated and Not Protected*	*Ever Vaccinated and Protected*
Infection – Fast	*Ever Vaccinated and Not Protected*	*Ever Vaccinated and Protected*
Infection – Slow	*Ever Vaccinated and Not Protected*	*Ever Vaccinated and Protected*
Subclinical Disease	*Ever Vaccinated and Not Protected*	*Ever Vaccinated and Not Protected*
Clinical Disease	*NA*	*NA*
On-treatment	*NA*	*NA*
Resolved	*Ever Vaccinated and Not Protected*	*Ever Vaccinated and Protected*

Waning, or loss of vaccine protection, moved individuals from the *Ever Vaccinated and Protected* compartment to the *Ever Vaccinated and Not Protected* compartment. We assumed duration of protection was 10 years on average, in addition to a sensitivity analysis with lifelong duration of protection. The shape of waning immunity was modelled as an exponential distribution, based on similar shapes for waning vaccine immunity of BCG[60] and other vaccines.[61,62]

7.3.1 Vaccine implementation in the tuberculosis natural history model

Vaccines are incorporated in the tuberculosis natural history structure as indicated with the orange boxes in Figure S7.3 by reducing the rate of progression to disease parameters into the subclinical disease compartment from the infection-fast, infection-slow, and resolved compartments by (1-p_V), where p_V is the vaccine efficacy. Vaccine efficacy was modelled as “degree”, also known as “leaky”. Degree vaccines assume that everyone who has been vaccinated receives some protection from the vaccine equivalent to the value of the vaccine efficacy.

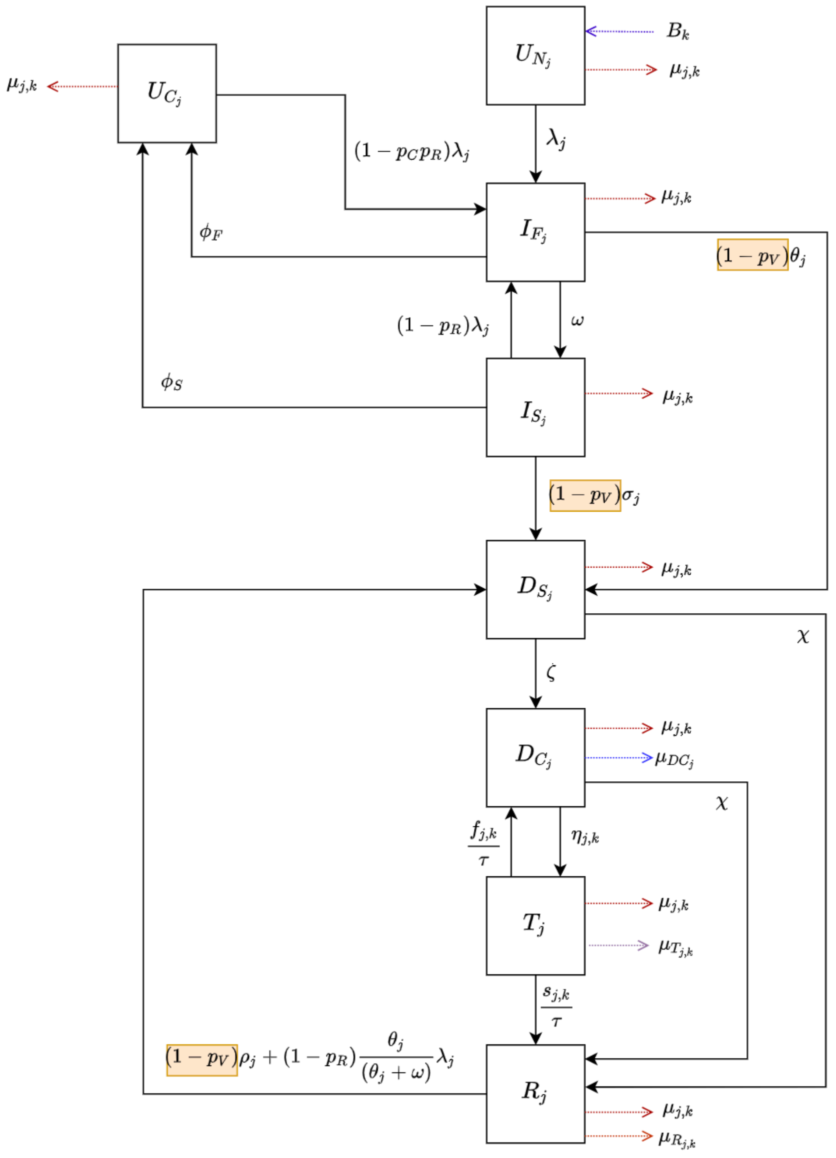

Figure S7.3 Tuberculosis natural history model incorporating vaccination

Abbreviations: D_C = Clinical Disease; D_S = Subclinical Disease; I_F = Infection-Fast; I_S = Infection-Slow; R = Resolved; T = On-Treatment; U_C = Uninfected-Cleared; U_N = Uninfected-Naive.

Subscript *j* represents parameters that vary by age, and subscript *k* represents parameters that vary over time.

8. Model outcomes

8.1 Epidemiological impact measures

The following measures were calculated for each vaccine scenario as the median and 95% uncertainty range

- Percent incidence rate reduction in 2050 for each vaccine scenario compared to the estimated value in 2050 by *No-New-Vaccine* baseline
- Incidence rate per 100,000 population in 2035 for each vaccine scenario
- Percent mortality rate reduction in 2050 for each vaccine scenario compared to the estimated value in 2050 by *No-New-Vaccine* baseline
- Cumulative cases averted for each vaccine scenario between vaccine introduction (either 2025 or country-specific years) and 2050 compared to the cumulative number of cases estimated by the *No-New-Vaccine* baseline between the corresponding years
- Cumulative deaths averted for each vaccine scenario between vaccine introduction (either 2025 or country-specific years) and 2050 compared to the cumulative number of cases estimated by the *No-New-Vaccine* baseline between the corresponding years
- Cumulative treatments averted for each vaccine scenario between vaccine introduction (either 2025 or country-specific years) and 2050 compared to the cumulative number of cases estimated by the *No-New-Vaccine* baseline between the corresponding years

8.2 Groupings for reporting model outcomes

The epidemiological impact measures were calculated and reported for the calibrated countries by WHO region, World Bank Income Group, for tuberculosis burden, and overall. Countries are divided into the six WHO regions,[63] [African region (AFR), region of the Americas (AMR), Eastern-Mediterranean region (EMR), South-East Asian region (SEAR), and Western-Pacific region (WPR)], three income groups based on the 2021 World Bank Income Groups[51] for low- and middle-income countries [low-income countries (LIC), lower-middle-income countries (LMIC) or upper-middle-income countries (UMIC)], and by whether they were or were not included on the WHO high TB burden list[64] (*High TB Burden* vs *Other* respectively). Groups for each of the LMICs are in Table S8.1.

Table S8.1 Country-specific introduction year, WHO region, 2021 World Bank income group, and WHO TB burden level for LMICs

Country	Gavi/Self Procuring	Introduction Year	WHO Region	2021 World Bank Income Group	WHO High TB Burden
Afghanistan	Gavi	2031	EMR	LIC	Other
Albania	Self-Procuring	2035	EUR	UMIC	Other
Algeria	Self-Procuring	2032	AFR	LMIC	Other
American Samoa	Self-Procuring	2044	WPR	UMIC	Other
Angola	Self-Procuring	2032	AFR	LMIC	High TB Burden
Argentina	Self-Procuring	2031	AMR	UMIC	Other
Armenia	Self-Procuring	2033	EUR	UMIC	Other
Azerbaijan	Self-Procuring	2028	EUR	UMIC	Other
Bangladesh	Gavi	2035	SEAR	LMIC	High TB Burden
Belarus	Self-Procuring	2028	EUR	UMIC	Other
Belize	Self-Procuring	2034	AMR	UMIC	Other
Benin	Gavi	2037	AFR	LMIC	Other
Bhutan	Self-Procuring	2034	SEAR	LMIC	Other
Bolivia	Self-Procuring	2037	AMR	LMIC	Other
Bosnia and Herzegovina	Self-Procuring	2039	EUR	UMIC	Other
Botswana	Self-Procuring	2028	AFR	UMIC	Other
Brazil	Self-Procuring	2030	AMR	UMIC	High TB Burden
Bulgaria	Self-Procuring	2029	EUR	UMIC	Other
Burkina Faso	Gavi	2039	AFR	LIC	Other
Burundi	Gavi	2044	AFR	LIC	Other
Cabo Verde	Self-Procuring	2043	AFR	LMIC	Other
Cambodia	Gavi	2036	WPR	LMIC	Other
Cameroon	Gavi	2031	AFR	LMIC	Other
Central African Republic	Gavi	2033	AFR	LIC	High TB Burden
Chad	Gavi	2033	AFR	LIC	Other
China	Self-Procuring	2029	WPR	UMIC	High TB Burden
Colombia	Self-Procuring	2030	AMR	UMIC	Other
Comoros	Gavi	2046	AFR	LMIC	Other
Congo	Gavi	2035	AFR	LMIC	High TB Burden
Costa Rica	Self-Procuring	2033	AMR	UMIC	Other
Côte d'Ivoire	Gavi	2034	AFR	LMIC	Other
Cuba	Self-Procuring	2035	AMR	UMIC	Other
Democratic People's Republic of Korea	Gavi	2035	SEAR	LIC	High TB Burden

Democratic Republic of the Congo	Gavi	2030	AFR	LIC	High TB Burden
Djibouti	Gavi	2043	EMR	LMIC	Other
Dominica	Self-Procuring	2031	AMR	UMIC	Other
Dominican Republic	Self-Procuring	2031	AMR	UMIC	Other
Ecuador	Self-Procuring	2033	AMR	UMIC	Other
Egypt	Self-Procuring	2033	EMR	LMIC	Other
El Salvador	Self-Procuring	2039	AMR	LMIC	Other
Equatorial Guinea	Self-Procuring	2042	AFR	UMIC	Other
Eritrea	Gavi	2047	AFR	LIC	Other
Ethiopia	Gavi	2030	AFR	LIC	High TB Burden
Fiji	Self-Procuring	2031	WPR	UMIC	Other
Gabon	Self-Procuring	2038	AFR	UMIC	High TB Burden
Gambia	Gavi	2039	AFR	LIC	Other
Georgia	Self-Procuring	2029	EUR	UMIC	Other
Ghana	Gavi	2040	AFR	LMIC	Other
Grenada	Self-Procuring	2035	AMR	UMIC	Other
Guatemala	Self-Procuring	2036	AMR	UMIC	Other
Guinea	Gavi	2033	AFR	LIC	Other
Guinea-Bissau	Gavi	2043	AFR	LIC	Other
Guyana	Self-Procuring	2030	AMR	UMIC	Other
Haiti	Gavi	2033	AMR	LIC	Other
Honduras	Self-Procuring	2037	AMR	LMIC	Other
India	Gavi	2033	SEAR	LMIC	High TB Burden
Indonesia	Self-Procuring	2034	SEAR	LMIC	High TB Burden
Iran	Self-Procuring	2031	EMR	LMIC	Other
Iraq	Self-Procuring	2033	EMR	UMIC	Other
Jamaica	Self-Procuring	2036	AMR	UMIC	Other
Jordan	Self-Procuring	2037	EMR	UMIC	Other
Kazakhstan	Self-Procuring	2028	EUR	UMIC	Other
Kenya	Gavi	2032	AFR	LMIC	High TB Burden
Kiribati	Self-Procuring	2041	WPR	LMIC	Other
Kosovo	Self-Procuring	2045	EUR	UMIC	Other
Kyrgyz Republic	Gavi	2044	EUR	LMIC	Other
Lao People's Democratic Republic	Gavi	2035	WPR	LMIC	Other
Lebanon	Self-Procuring	2038	EMR	UMIC	Other
Lesotho	Gavi	2039	AFR	LMIC	High TB Burden
Liberia	Gavi	2037	AFR	LIC	High TB Burden
Libya	Self-Procuring	2035	EMR	UMIC	Other

Madagascar	Gavi	2031	AFR	LIC	Other
Malawi	Gavi	2038	AFR	LIC	Other
Malaysia	Self-Procuring	2028	WPR	UMIC	Other
Maldives	Self-Procuring	2034	SEAR	UMIC	Other
Mali	Gavi	2037	AFR	LIC	Other
Marshall Islands	Self-Procuring	2041	WPR	UMIC	Other
Mauritania	Gavi	2042	AFR	LMIC	Other
Mexico	Self-Procuring	2029	AMR	UMIC	Other
Micronesia	Self-Procuring	2045	WPR	LMIC	Other
Mongolia	Self-Procuring	2032	WPR	LMIC	High TB Burden
Montenegro	Self-Procuring	2044	EUR	UMIC	Other
Morocco	Self-Procuring	2029	EMR	LMIC	Other
Mozambique	Gavi	2032	AFR	LIC	High TB Burden
Myanmar	Gavi	2031	SEAR	LMIC	High TB Burden
Namibia	Self-Procuring	2030	AFR	UMIC	High TB Burden
Nepal	Gavi	2036	SEAR	LMIC	Other
Nicaragua	Gavi	2047	AMR	LMIC	Other
Niger	Gavi	2036	AFR	LIC	Other
Nigeria	Gavi	2030	AFR	LMIC	High TB Burden
North Macedonia	Self-Procuring	2038	EUR	UMIC	Other
Pakistan	Gavi	2031	EMR	LMIC	High TB Burden
Papua New Guinea	Gavi	2032	WPR	LMIC	High TB Burden
Paraguay	Self-Procuring	2035	AMR	UMIC	Other
Peru	Self-Procuring	2029	AMR	UMIC	Other
Philippines	Self-Procuring	2030	WPR	LMIC	High TB Burden
Republic of Moldova	Self-Procuring	2034	EUR	UMIC	Other
Russian Federation	Self-Procuring	2030	EUR	UMIC	Other
Rwanda	Gavi	2045	AFR	LIC	Other
Samoa	Self-Procuring	2046	WPR	UMIC	Other
Sao Tome and Principe	Gavi	2044	AFR	LMIC	Other
Senegal	Gavi	2038	AFR	LMIC	Other
Serbia	Self-Procuring	2036	EUR	UMIC	Other
Sierra Leone	Gavi	2037	AFR	LIC	High TB Burden
Solomon Islands	Gavi	2047	WPR	LMIC	Other
Somalia	Gavi	2030	EMR	LIC	Other
South Africa	Self-Procuring	2029	AFR	UMIC	High TB Burden
South Sudan	Gavi	2034	AFR	LIC	Other
Sri Lanka	Self-Procuring	2028	SEAR	LMIC	Other

St. Lucia	Self-Procuring	2031	AMR	UMIC	Other
St. Vincent and the Grenadines	Self-Procuring	2032	AMR	UMIC	Other
Sudan	Gavi	2036	EMR	LIC	Other
Suriname	Self-Procuring	2040	AMR	UMIC	Other
Swaziland	Self-Procuring	2036	AFR	LMIC	Other
Syrian Arab Republic	Gavi	2036	EMR	LIC	Other
Tajikistan	Gavi	2045	EUR	LMIC	Other
Thailand	Self-Procuring	2031	SEAR	UMIC	High TB Burden
Timor-Leste	Self-Procuring	2031	SEAR	LMIC	Other
Togo	Gavi	2041	AFR	LIC	Other
Tonga	Self-Procuring	2040	WPR	UMIC	Other
Tunisia	Self-Procuring	2036	EMR	LMIC	Other
Turkey	Self-Procuring	2030	EUR	UMIC	Other
Turkmenistan	Self-Procuring	2034	EUR	UMIC	Other
Tuvalu	Self-Procuring	2043	WPR	UMIC	Other
Uganda	Gavi	2034	AFR	LIC	High TB Burden
Ukraine	Self-Procuring	2033	EUR	LMIC	Other
United Republic of Tanzania	Gavi	2031	AFR	LMIC	High TB Burden
Uzbekistan	Gavi	2038	EUR	LMIC	Other
Vanuatu	Self-Procuring	2042	WPR	LMIC	Other
Venezuela	Self-Procuring	2035	AMR	UMIC	Other
Vietnam	Self-Procuring	2038	WPR	LMIC	High TB Burden
West Bank and Gaza	Self-Procuring	2043	EMR	LMIC	Other
Yemen	Gavi	2036	EMR	LIC	Other
Zambia	Gavi	2034	AFR	LIC	High TB Burden
Zimbabwe	Gavi	2032	AFR	LMIC	Other

Abbreviations: AFR = WHO African Region, AMR = WHO Region of the Americas, EMR = WHO Eastern Mediterranean Region, EUR = WHO European Region, LIC = low-income countries, LMIC = lower-middle income countries, SEAR = WHO South-East Asian Region, UMIC = upper-middle income countries, WPR = WHO Western Pacific Region

8.2 Calculating uncertainty

To appropriately represent the global uncertainty and remove inter-country variability in parameters that are likely to be the same across countries when generating impact estimates (e.g., those governing the underlying biology of *Mtb*), we used the following process:

1. We obtained 1000 fitted parameter sets for each country by thinning the total number of fitted parameter sets per country to 1000.

2. Within each country, the 1000 parameter sets were ordered and ranked from smallest to largest by 2019 tuberculosis incidence rate.

3. The parameter sets for all countries were then pairwise grouped on their rank value. For example, the rank 1 parameter sets were grouped together for all countries, the rank 2 parameter sets were grouped together for all countries, etc.

4. Within each pairwise rank group, we calculated the measure of interest by combining all information. For example, to calculate the incidence rate, we summed the number of cases from all countries with rank 1 and divided by the sum of the population for all countries with rank 1. This was continued for all ranks until there were 1000 estimates of the measure of interest.

5. We combined the 1000 estimates for the measure of interest, generated the distribution and calculated all country and group-level estimates.

SUPPLEMENTAL RESULTS:

9. Model Calibration

9.1 LMIC calibration

Of the 135 LMICs, there were 20 countries which were excluded from calibration due to missing crucial data for calibration (as described in Table S5.3). The 10 countries that did not calibrate out of the 115 worth running were: Algeria, Bosnia and Herzegovina, Cabo Verde, Guinea-Bissau, Guyana, Jamaica, North Macedonia, St. Vincent and the Grenadines, Tonga, and Turkmenistan. Reasons for why the ten countries were unable to be calibrated have been thoroughly explored by our colleagues Scarponi et al, where the authors provided strong evidence that the models were misspecified and could not be calibrated to the target ranges.[65] Of the 105 calibrated countries, 21 countries were classified as having a higher tuberculosis burden due to HIV.

9.2 WHO Region and 2021 World Bank Income Group for Calibrated LMICs

Table S9.1 WHO regions and 2021 World bank income groups for the 105 calibrated LMICs

**WHO Region**	**World Bank Income Level 2019**			***Total***
	LIC	LMIC	UMIC	
AFR	20	14	5	39
AMR	0	4	13	17
EMR	4	5	3	12
EUR	0	4	12	16
SEAR	0	8	2	10
WPR	0	8	3	11
Total	24	43	38	105

Abbreviations: AFR = WHO African Region, AMR = WHO Region of the Americas, EMR = WHO Eastern Mediterranean Region, EUR = WHO European Region, LIC = low-income countries, LMIC = lower-middle income countries, SEAR = WHO South-East Asian Region, UMIC = upper-middle income countries, WPR = WHO Western Pacific Region

9.3 *Status Quo No-New-Vaccine* baseline calibration target values

Table S9.2 presents the common country-specific calibration targets for the 105 calibrated countries, and Table S9.3 highlights the HIV specific calibration targets for the 21 countries classified as having a higher tuberculosis burden due to HIV. Two additional calibration targets were assumed consistent across countries: the global fraction of subclinical tuberculosis among active tuberculosis in 2019 [50·4% (36·1%–79·7%)],[1] and the risk ratio of active tuberculosis in the high-access-to-care group relative to low-access-to-care in 2019 [0·674 (0·575, 0·801)].[12–22]

Table S9.2 Values for the seven country specific calibration targets for all calibrated countries in 2019. Point values represent the mean with 95% confidence intervals in brackets

Country	Tuberculosis Incidence Rate[23,53] (per 100,000 population per year)			Tuberculosis Notification Rate[23,27] (per 100,000 population per year)			Tuberculosis Mortality Rate[23,26] (per 100,000 population per year)
	Ages 0–14	Ages 15+	Ages 0–99	Ages 0–14	Ages 15+	Ages 0–99	Ages 0–99
Afghanistan	92·8 (48·9–136·2)	260·5 (137·1–383·8)	189·3 (115·7–262·9)	71·0 (56·8–85·2)	187·2 (149·7–224·6)	137·8 (110·3–165·4)	26 (16–39)
Albania	2·6 (2·2–3·0)	19·3 (16·4–22·3)	16·3 (13·9–18·7)	2·2 (1·8–2·6)	16·9 (13·5–20·2)	14·3 (11·4–17·2)	0·3 (0·2–0·5)
Angola	107·8 (58·6–155·0)	565·1 (312·0–818·2)	351·9 (213·7–487·0)	59·4 (47·5–71·3)	384·3 (307·5–461·2)	232·8 (186·3–279·4)	62 (39–89)
Argentina	9·1 (7·9–10·9)	35·5 (29·6–41·5)	29·0 (24·6–33·5)	8·1 (6·5–9·8)	31·2 (24·9–37·4)	25·6 (20·4–30·7)	1·6 (1·4–1·8)
Armenia	7·7 (5·7–9·8)	31·1 (23·0–39·3)	26·4 (19·6–32·8)	6·2 (4·9–7·4)	24·9 (19·9–29·9)	21·0 (16·8–25·2)	0·6 (0·4–0·8)
Azerbaijan	19·5 (14·0–24·6)	72·8 (53·3–92·3)	59·7 (44·8–74·6)	7·6 (6·1–9·1)	44·6 (35·7–53·6)	48·0 (38·4–57·6)	6·1 (5·6–6·6)
Bangladesh	74·4 (49·6–96·9)	276·4 (187·9–364·9)	221·4 (156·4–285·8)	27·8 (22·2–33·3)	235·3 (188·3–282·4)	178·8 (143·1–214·6)	24 (15–35)
Belarus	4·3 (3·2–5·3)	34·4 (25·5–43·4)	29·6 (22·2–36·0)	0·5 (0·4–0·6)	28·0 (22·4–33·6)	23·3 (18·7–28·0)	3·3 (3·1–3·5)
Benin	11·4 (6·6–16·3)	86·5 (49·9–124·6)	55·1 (33·9–77·1)	4·6 (3·7–5·6)	59·2 (47·3–71·0)	36·1 (28·9–43·4)	11 (7·2–15)
Bhutan	32·1 (23·8–40·9)	210·6 (154·5–263·3)	170·4 (123·2–209·7)	14·5 (11·6–17·4)	171·5 (137·2–205·8)	131·7 (105·4–158·0)	18 (12–26)

Bolivia	25·8<br>(15·3–36·9)	137·7<br>(82·6–200·3)	104·2<br>(65·1–147·7)	6·9<br>(5·6–8·3)	90·3<br>(72·3–108·4)	64·8<br>(51·8–77·8)	11<br>(8·1–15)
Botswana	74·6<br>(55·3–93·9)	340·8<br>(249·0–432·5)	251·8<br>(191·0–312·5)	29·8<br>(23·9–35·8)	187·2<br>(149·8–224·7)	134·1<br>(107·3–160·9)	78<br>(61–96)
Brazil	7·0<br>(5·9–8·1)	55·8<br>(47·4–64·2)	45·5<br>(38·9–52·1)	6·0<br>(4·8–7·3)	48·5<br>(38·8–58·2)	39·6<br>(31·7–47·5)	3·2<br>(2·9–3·4)
Bulgaria	8·8<br>(6·4–10·7)	21·8<br>(16·6–28·5)	20·0<br>(15·7–25·7)	5·3<br>(4·2–6·3)	20·7<br>(16·5–24·8)	18·4<br>(14·7–22·1)	1·6<br>(1·6–1·7)
Burkina Faso	9·8<br>(5·6–14·3)	77·4<br>(44·5–106·7)	47·2<br>(28·5–64·0)	1·5<br>(1·2–1·8)	42·0<br>(33·6–50·4)	24·2<br>(19·4–29·0)	11<br>(7·2–16)
Burundi	24·8<br>(13·8–34·4)	174·7<br>(100·1–254·1)	104·1<br>(65·0–147·4)	6·2<br>(5·0–7·4)	102·1<br>(81·7–122·5)	58·6<br>(46·9–70·3)	24<br>(15–35)
Cambodia	68·3<br>(39·0–97·5)	387·3<br>(220·1–545·8)	285·1<br>(175·9–400·3)	36·1<br>(28·9–43·3)	247·0<br>(197·6–296·4)	181·4<br>(145·1–217·7)	20<br>(13–28)
Cameroon	43·8<br>(24·6–62·0)	274·9<br>(154·2–395·6)	177·8<br>(108·2–247·3)	11·4<br>(9·2–13·7)	154·6<br>(123·7–185·6)	94·0<br>(75·2–112·8)	48<br>(34–65)
Central African Republic	182·3<br>(100·8–263·9)	826·7<br>(450·9–1202·5)	547·9<br>(337·2–758·7)	79·1<br>(63·3–94·9)	391·2<br>(313·0–469·5)	253·9<br>(203·1–304·6)	158<br>(111–215)
Chad	36·2<br>(20·1–52·2)	235·8<br>(129·7–342·0)	144·2<br>(87·8–194·4)	14·0<br>(11·2–16·8)	148·5<br>(118·8–178·2)	85·5<br>(68·4–102·6)	31<br>(21–42)
China	14·9<br>(12·5–16·8)	67·5<br>(57·4–77·6)	58·1<br>(49·7–66·5)	2·6<br>(2·1–3·1)	61·2<br>(49·0–73·5)	50·8<br>(40·6–61·0)	2·3<br>(2·1–2·6)
Colombia	7·2<br>(5·4–8·8)	43·6<br>(33·4–53·9)	35·8<br>(25·8–43·7)	3·5<br>(2·8–4·2)	35·7<br>(28·5–42·8)	28·4<br>(22·7–34·1)	3·4<br>(3–3·9)
Costa Rica	1·7<br>(1·2–2·1)	12·3<br>(9·0–15·6)	10·1<br>(7·5–12·5)	1·3<br>(1·1–1·6)	9·8<br>(7·9–11·8)	8·0<br>(6·4–9·6)	0·8<br>(0·7–0·9)
Cote d'Ivoire	30·8<br>(17·7–43·8)	213·5<br>(120·1–306·9)	136·1<br>(81·7–190·5)	9·5<br>(7·6–11·4)	134·7<br>(107·8–161·6)	82·5<br>(66·0–99·0)	30<br>(20–41)
Cuba	0·4<br>(0·3–0·5)	7·6<br>(6·4–8·7)	6·4<br>(5·5–7·4)	0·3<br>(0·2–0·4)	6·6<br>(5·3–7·9)	5·6<br>(4·5–6·7)	0·4<br>(0·3–0·5)
Dominican Republic	6·7<br>(5·0–8·4)	55·4<br>(41·2–69·6)	41·9<br>(31·7–52·1)	2·7<br>(2·2–3·2)	45·2<br>(36·2–54·3)	33·4<br>(26·8–40·1)	4<br>(2·4–6)
Ecuador	8·7<br>(6·4–11·0)	59·7<br>(43·8–75·6)	45·5<br>(34·5–57·0)	3·9<br>(3·1–4·7)	49·1<br>(39·2–58·9)	36·5<br>(29·2–43·8)	4·6<br>(3·9–5·3)
Egypt	2·6<br>(2·3–2·9)	16·6<br>(14·2–18·1)	12·0<br>(10·0–12·9)	1·4<br>(1·1–1·7)	11·4<br>(9·1–13·7)	8·0<br>(6·4–9·6)	0·5<br>(0·4–0·5)

El Salvador	18·5 (13·3–23·7)	72·0 (53·0–93·2)	58·9 (43·4–72·8)	7·8 (6·2–9·3)	53·7 (43·0–64·5)	46·6 (37·3–56·0)	1·7 (1·4–2)
Equatorial Guinea	45·9 (39·9–53·9)	257·3 (222·3–292·4)	184·4 (154·9–206·5)	19·2 (15·3–23·0)	169·0 (135·2–202·8)	114·3 (91·4–137·2)	39 (31–48)
Eritrea	29·6 (7·6–51·6)	127·3 (32·8–220·3)	85·8 (31·5–143·0)	15·8 (12·7–19·0)	78·2 (62·6–93·9)	52·3 (41·8–62·7)	17 (8–29)
Ethiopia	37·6 (24·3–53·1)	207·9 (133·1–282·6)	140·1 (94·6–184·7)	24·4 (19·5–29·3)	149·6 (119·7–179·5)	99·1 (79·3–118·9)	22 (14–30)
Fiji	57·5 (38·4–76·7)	69·9 (47·7–92·2)	66·3 (49·4–83·2)	47·2 (37·7–56·6)	55·5 (44·4–66·6)	53·0 (42·4–63·6)	5·3 (4·9–5·7)
Gabon	160·9 (92·8–235·2)	732·7 (410·3–1025·8)	506·3 (317·6–736·5)	45·4 (36·3–54·5)	368·7 (295·0–442·4)	248·5 (198·8–298·2)	110 (66–165)
Gambia	37·6 (26·1–50·2)	251·6 (175·3–327·8)	157·6 (115·0–200·2)	12·5 (10·0–15·1)	189·8 (151·8–227·7)	111·6 (89·2–133·9)	27 (20–35)
Georgia	20·0 (16·2–23·7)	87·6 (72·0–103·2)	75·1 (62·6–87·6)	9·0 (7·2–10·8)	65·6 (52·5–78·7)	54·3 (43·4–65·1)	4·2 (3·8–4·6)
Ghana	55·4 (16·7–96·8)	194·2 (57·7–335·9)	144·7 (55·9–230·1)	7·2 (5·8–8·7)	72·8 (58·2–87·3)	48·3 (38·6–58·0)	50 (29–77)
Guatemala	10·9 (7·9–14·1)	34·4 (24·1–43·9)	26·2 (19·9–33·0)	7·4 (5·9–8·9)	28·0 (22·4–33·6)	21·1 (16·9–25·4)	2·4 (2·1–2·6)
Guinea	37·8 (21·6–54·1)	276·9 (166·2–401·5)	172·3 (109·6–242·7)	20·9 (16·7–25·0)	210·9 (168·7–253·1)	128·3 (102·7–154·0)	29 (20–39)
Honduras	4·3 (3·2–5·3)	43·2 (31·3–55·1)	30·8 (23·6–39·0)	1·6 (1·3–1·9)	35·4 (28·3–42·5)	24·9 (19·9–29·8)	5·0 (4·2–5·8)
India	91·6 (55·8–127·6)	229·4 (139·6–320·1)	193·2 (125·9–259·8)	40·0 (32·0–48·0)	201·1 (160·9–241·4)	158·2 (126·6–189·9)	33 (30–35)
Indonesia	200·2 (179·0–221·3)	352·1 (314·5–389·6)	312·2 (284·2–340·3)	98·8 (79·0–118·6)	245·5 (196·4–294·6)	207·7 (166·1–249·2)	36 (33–38)
Iran	3·0 (2·3–3·9)	16·0 (11·8–20·8)	13·3 (9·6–15·7)	1·6 (1·2–1·9)	13·1 (10·5–15·8)	10·3 (8·2–12·3)	1·2 (1·1–1·2)
Iraq	12·7 (10·7–14·7)	57·5 (49·3–65·7)	40·7 (35·6–45·8)	3·8 (3·0–4·6)	24·8 (19·9–29·8)	16·8 (13·5–20·2)	2·1 (1·9–2·3)
Jordan	1·1 (0·8–1·4)	7·7 (5·7–9·8)	5·5 (4·2–6·9)	0·9 (0·7–1·0)	6·2 (5·0–7·5)	4·4 (3·5–5·3)	0·1 (0·1–0·2)
Kazakhstan	6·7 (3·9–9·3)	91·0 (56·1–128·9)	70·1 (41·5–97·0)	6·6 (5·2–7·9)	92·1 (73·7–110·5)	67·4 (53·9–80·9)	1·9 (1·4–2·4)

Kenya	87·4 (43·2–126·2)	384·8 (193·9–572·4)	266·3 (150·3–382·3)	40·3 (32·2–48·3)	237·9 (190·3–285·5)	160·4 (128·3–192·5)	62 (42–85)
Kyrgyz Republic	16·8 (14·4–19·7)	154·7 (131·6–180·1)	110·7 (93·5–126·2)	14·6 (11·7–17·6)	134·7 (107·8–161·6)	95·7 (76·5–114·8)	5·9 (5·4–6·3)
Lao PDR	47·5 (25·9–64·8)	206·0 (119·5–288·5)	153·4 (94·8–209·2)	4·1 (3·3–4·9)	138·5 (110·8–166·2)	95·1 (76·1–114·1)	30 (19–44)
Lesotho	137·7 (75·4–202·9)	905·8 (494·7–1323·8)	658·7 (376·4–941·1)	41·2 (32·9–49·4)	472·0 (377·6–566·4)	332·1 (265·7–398·6)	224 (153–309)
Liberia	129·2 (69·6–188·9)	444·4 (232·5–615·3)	303·8 (188·4–425·3)	70·2 (56·2–84·3)	234·8 (187·8–281·7)	167·9 (134·3–201·4)	74 (49–103)
Libya	15·8 (8·4–23·1)	75·9 (41·0–110·8)	59·0 (33·9–84·1)	5·4 (4·3–6·5)	43·2 (34·6–51·8)	32·7 (26·1–39·2)	12 (7·1–19)
Madagascar	69·8 (38·6–101·0)	342·0 (192·8–497·4)	233·6 (140·9–322·6)	28 (22·4–33·6)	212·4 (169·9–254·9)	138·0 (110·4–165·6)	46 (27–68)
Malawi	48·2 (18·5–77·8)	218·4 (85·5–360·8)	144·9 (69·8–225·5)	18·9 (15·1–22·6)	146·0 (116·8–175·2)	90·7 (72·6–108·9)	37 (24–53)
Malaysia	12·9 (11·0–14·5)	114·9 (98·4–135·4)	90·8 (78·2–106·4)	11·2 (9·0–13·5)	101·4 (81·1–121·7)	80·0 (64·0–96·0)	4·8 (4·3–5·3)
Maldives	3·8 (2·8–4·7)	44·7 (32·9–56·4)	35·8 (26·4–45·2)	2·8 (2·3–3·4)	35·5 (28·4–42·6)	29·0 (23·2–34·8)	2·1 (1·9–2·3)
Mali	9·8 (5·7–14·0)	90·7 (53·1–125·5)	50·9 (32·6–71·2)	3·7 (3·0–4·4)	63·4 (50·7–76·1)	35·2 (28·1–42·2)	9·1 (6–13)
Mauritania	22·2 (12·7–31·6)	132·3 (77·2–191·1)	88·4 (55·2–123·7)	8·6 (6·9–10·4)	85·4 (68·3–102·5)	55·1 (44·1–66·1)	17 (10–25)
Mexico	3·9 (3·0–5·1)	29·7 (22·3–38·2)	23·5 (17·2–29·0)	2·0 (1·6–2·4)	24·5 (19·6–29·4)	18·6 (14·9–22·3)	2 (1·9–2·2)
Mongolia	201·3 (70·5–332·2)	537·7 (183·7–851·3)	434·1 (186–682·1)	40·9 (32·7–49·0)	173·4 (138·8–208·1)	132·6 (106·1–159·1)	10 (9·2–12)
Montenegro	0·9 (0·7–1·1)	17·7 (15·0–19·5)	14·6 (12·4–17·5)	0·9 (0·7–1·1)	15·4 (12·3–18·4)	12·7 (10·2–15·3)	0·2 (0·2–0·2)
Morocco	24·4 (20·3–28·5)	123·9 (105·1–142·7)	96·0 (82·3–112·4)	21·2 (17·0–25·5)	107·6 (86·1–129·2)	84·3 (67·5–101·2)	8·1 (5–12)
Mozambique	259·8 (111·3–400·8)	444·0 (189·4–692·6)	362·2 (207·5–517·0)	95·4 (76·3–114·5)	492·3 (393·9–590·8)	316·2 (253·0–379·5)	37 (25–52)
Myanmar	271·3 (142·8–392·7)	339·7 (182·3–497·0)	322·0 (199·8–442·2)	169·2 (135·4–203·1)	276·7 (221·3–332·0)	248·9 (199·1–298·6)	41 (27–59)

Namibia	152·1 (99·9–206·4)	698·9 (444·8–889·5)	481·1 (336·7–641·4)	79·6 (63·7–95·5)	449·1 (359·3–538·9)	312·7 (250·2–375·3)	107 (80–137)
Nepal	60·3 (30·7–89·9)	312·6 (158·8–466·4)	237·7 (129·3–346·0)	20·4 (16·3–24·5)	147·2 (117·8–176·7)	110·1 (88·1–132·1)	59 (32–92)
Nicaragua	13·8 (10·2–17·9)	54·5 (39·2–69·7)	42·8 (32·1–53·5)	8·9 (7·1–10·6)	45·2 (36·2–54·2)	34·3 (27·5–41·2)	2·3 (1·9–2·6)
Niger	17·2 (9·5–24·1)	153·9 (85·5–213·8)	85·8 (51·5–115·8)	4·6 (3·6–5·5)	93·9 (75·1–112·7)	49·3 (39·4–59·1)	17 (10–25)
Nigeria	94·5 (51·3–137·8)	316·3 (170·5–461·3)	218·9 (134·9–303·0)	10·8 (8·6–12·9)	95·2 (76·1–114·2)	58·4 (46·7–70·1)	77 (49–110)
Pakistan	100·1 (64·5–137·0)	351·2 (224·7–477·8)	263·2 (180·1–346·3)	59·9 (47·9–71·8)	200·9 (160·7–241·0)	151·6 (121·3–181·9)	20 (16–25)
Papua New Guinea	353·2 (256·9–417·4)	476·9 (353·2–600·5)	433·0 (353·2–512·8)	220·2 (176·2–264·3)	402·2 (321·8–482·7)	342·6 (274·1–411·1)	50 (34–70)
Paraguay	11·2 (9·2–12·6)	60·1 (50·1–70·2)	46·8 (39·7–52·5)	9·6 (7·7–11·5)	52·9 (42·3–63·4)	40·2 (32·2–48·3)	4·5 (3·8–5·2)
Peru	26·8 (19·5–34·1)	148·1 (111·1–189·3)	120·0 (89·2–147·6)	16·6 (13·3–19·9)	123·4 (98·7–148·0)	97·7 (78·2–117·2)	8·8 (7·2–11)
Philippines	209·4 (91·0–324·7)	705·1 (310·0–1101·5)	554·0 (276·6–831·5)	129·5 (103·6–155·4)	487·5 (390·0–585·1)	378·4 (302·8–454·1)	26 (22–29)
Republic of Moldova	18·7 (15·3–20·2)	91·2 (76·5–105·9)	79·1 (66·8–91·5)	15·7 (12·6–18·9)	79·6 (63·7–95·6)	69·5 (55·6–83·4)	6·2 (5·4–7·1)
Russian Federation	7·6 (4·5–10·6)	59·5 (35·2–82·9)	50·0 (30·2–69·2)	7·7 (6·1–9·2)	59·2 (47·4–71·1)	50·3 (40·2–60·3)	6·7 (6–7·4)
Rwanda	12·3 (9·0–15·7)	86·8 (63·1–110·4)	57·0 (42·8–71·3)	8·6 (6·9–10·4)	72·0 (57·6–86·4)	45·7 (36·5–54·8)	7·5 (5·5–9·7)
Sao Tome and Principe	21·0 (4·4–38·6)	184·9 (36·2–329·5)	116·3 (29·8–200·0)	12·1 (9·7–14·6)	103·7 (82·9–124·4)	65·1 (52·1–78·1)	26 (13–44)
Senegal	21·5 (14·3–30·1)	182·5 (128·8–246·9)	116·6 (79·8–153·4)	8·5 (6·8–10·2)	137·0 (109·6–164·4)	82·0 (65·6–98·4)	18 (12–25)
Serbia	1·3 (1·1–1·5)	17·5 (14·8–18·9)	14·8 (12·5–17·1)	1·1 (0·9–1·3)	14·4 (11·5–17·2)	12·6 (10·1–15·1)	0·5 (0·5–0·6)
Sierra Leone	100·6 (56·6–147·7)	431·8 (237·5–626·2)	294·4 (179·2–409·6)	73·9 (59·1–88·6)	333·4 (266·7–400·0)	227·7 (182·2–273·3)	40 (26–56)
Solomon Islands	24·6 (17·5–31·6)	94·8 (67·3–122·2)	65·7 (49·3–82·1)	19·7 (15·8–23·7)	75·1 (60·0–90·1)	52·8 (42·3–63·4)	7·3 (4·7–10)

South Africa	224·0 (141·5–306·5)	774·1 (488·0–1060·2)	614·8 (409·8–818)	97·0 (77·6–116·4)	464·2 (371·4–557·0)	357·8 (286·3–429·4)	99 (59–150)
South Sudan	106·6 (56·5–158·8)	309·4 (170·2–464·1)	226·0 (135·6–316·4)	72·8 (58·2–87·3)	200·8 (160·6–240·9)	147·6 (118·0–177·1)	42 (28–60)
Sri Lanka	15·5 (10·8–19·6)	80·2 (54·9–104·8)	65·7 (45·5–79·7)	4·6 (3·7–5·6)	49·1 (39·3–59·0)	38·5 (30·8–46·2)	3·6 (2·9–4·4)
Sudan	19·8 (12·2–27·3)	97·6 (58·5–136·6)	67·7 (44·4–88·8)	11·3 (9·1–13·6)	68·3 (54·6–81·9)	46·2 (37·0–55·5)	10 (6·6–15)
Suriname	4·5 (3·8–5·8)	37·7 (28·2–47·1)	29·2 (20·6–36·1)	3·8 (3·1–4·6)	29·9 (23·9–35·9)	22·9 (18·3–27·5)	4·5 (3·7–5·4)
Swaziland	80·6 (43·7–117·4)	532·4 (294·2–770·6)	365·8 (209·0–513·9)	40·5 (32·4–48·6)	378·3 (302·6–453·9)	250·5 (200·4–300·6)	84 (55–118)
Syrian Arab Republic	6·6 (4·7–8·3)	24·6 (17·8–31·4)	18·7 (14·1–23·4)	4·2 (3·4–5·1)	20·1 (16·1–24·1)	15·2 (12·1–18·2)	0·1 (0·1–0·1)
Tajikistan	18·2 (13·3–22·9)	121·0 (88·7–153·4)	82·6 (62·2–103·0)	11·7 (9·4–14·0)	91·2 (73–109·5)	61·7 (49·4–74·1)	8·5 (7·6–9·4)
Thailand	28·2 (20·5–35·9)	174·4 (127·8–221·0)	150·8 (112·0–188·1)	7·5 (6·0–9·0)	147·5 (118·0–177·0)	126·1 (100·9–151·3)	16 (13–21)
Timor-Leste	157·6 (89·2–228·1)	702·9 (394·6–1011·2)	494·9 (301·6–696·0)	75·5 (60·4–90·6)	454·6 (363·6–545·5)	313·2 (250·6–375·8)	90 (54–134)
Togo	5·7 (4·5–6·6)	58·7 (46·1–71·3)	37·1 (29·7–44·5)	2·6 (2·1–3·1)	52·2 (41·8–62·7)	31·9 (25·5–38·2)	3·6 (2·5–5)
Tunisia	11·3 (8·1–14·1)	42·9 (31·6–54·2)	35·1 (26·5–44·5)	7·5 (6·0–9·0)	34·8 (27·8–41·8)	28·2 (22·6–33·8)	1·3 (0·9–1·6)
Turkey	2·8 (2·4–3·3)	19·0 (15·8–22·2)	15·6 (13·2–18·0)	2·4 (2·0–2·9)	17·0 (13·6–20·4)	13·5 (10·8–16·2)	0·4 (0·3–0·4)
Uganda	77·7 (35·0–121·4)	304·0 (135·1–472·9)	198·8 (106·2–293·7)	39·9 (31·9–47·9)	238·1 (190·5–285·7)	148·9 (119·1–178·6)	35 (24–48)
Ukraine	18·5 (10·7–25·7)	89·2 (51·4–124·4)	77·3 (47·7–106·8)	8·3 (6·7–10·0)	67·0 (53·6–80·5)	57·7 (46·2–69·2)	12 (9·9–13)
United Republic of Tanzania	82·6 (22·8–145·5)	356·1 (98·2–617·1)	236·2 (89·6–384·4)	48·1 (38·5–57·8)	211·7 (169·4–254·1)	140·0 (112·0–168·0)	55 (32–84)
Uzbekistan	33·7 (21·1–46·3)	80·9 (51·1–110·7)	66·7 (45·5–91·0)	23·1 (18·4–27·7)	60·0 (48·0–72·0)	49·3 (39·5–59·2)	5·4 (5·0–5·8)
Vanuatu	16·4 (11·2–20·7)	59·8 (40·3–76·2)	40·0 (30·7–53·4)	12·1 (9·6–14·5)	43·0 (34·4–51·6)	31·0 (24·8–37·2)	5·4 (3·5–7·7)

Venezuela	11·5<br>(8·4–14·1)	58·0<br>(42·5–72·5)	45·6<br>(34·0–56·1)	7·2<br>(5·8–8·7)	47·0<br>(37·6–56·4)	36·1<br>(28·9–43·3)	3·4<br>(2·7–4·2)
Vietnam	35·7<br>(20·5–49·1)	218·7<br>(126·9–311·9)	176·2<br>(105·7–247·8)	7·6<br>(6·1–9·1)	136·0<br>(108·8–163·2)	106·3<br>(85·0–127·5)	12<br>(8·0–16)
Yemen	15·7<br>(14·0–18·4)	67·7<br>(56·4–79·0)	48·0<br>(41·1–54·9)	10·8<br>(8·7–13·0)	50·7<br>(40·5–60·8)	35·1<br>(28·0–42·1)	6·6<br>(4·7–8·9)
Zambia	80·6<br>(45·3–114·6)	534·3<br>(302·4–766·2)	330·3<br>(201·6–459·1)	31·1<br>(24·9–37·4)	339·5<br>(271·6–407·4)	202·4<br>(161·9–242·9)	86<br>(62–114)
Zimbabwe	43·7<br>(30·8–56·7)	306·9<br>(212·5–413·2)	198·0<br>(143·4–252·6)	19·0<br>(15·2–22·8)	234·2<br>(187·3–281·0)	143·4<br>(114·8–172·1)	43<br>(33–54)

Table S9.3 **HIV specific calibration targets for countries classified as having a higher tuberculosis burden due to HIV for 2019 and for ages 0–99. Point values represent the mean with 95% confidence intervals in brackets**

Country	HIV Prevalence[23,52] (%)	ART Coverage[52] (%)	Tuberculosis Incidence Rate in PLHIV[26] (per 100,000 population per year)	Tuberculosis Mortality Rate in PLHIV[26] (per 100,000 population per year)
Botswana	16·5 (14·8–17·8)	82 (74–89)	123 (95–155)	49 (23–81)
Central African Republic	2·1 (1·8–2·7)	46 (37–59)	137 (88–195)	61 (17–115)
Côte d'Ivoire	1·7 (1·4–1·9)	63 (54–74)	24 (16–35)	8·5 (2·3–15·5)
Cameroon	2·0 (1·7–2·2)	62 (54–68)	48 (31–69)	19 (5–37)
Gabon	2·3 (1·7–3·0)	51 (38–66)	171 (65–327)	26 (0–194)
Ghana	1·1 (0·8–1·5)	45 (32–61)	30 (15–52)	16 (0–40)
Gambia	1·2 (0·9–1·5)	29 (23–37)	28 (21–37)	7·9 (3·3–12·1)
Equatorial Guinea	4·8 (3·5–6·5)	35 (27–48)	48 (41–55)	16 (8–24)
Kenya	2·9 (2·5–3·2)	74 (65–86)	70 (43–104)	24 (6–48)
Lesotho	16·0 (15·1–16·9)	65 (61–70)	403 (250–591)	168 (38–328)
Mozambique	7·2 (5·9–9·2)	60 (48–74)	122 (75–180)	18 (4–38)
Malawi	5·9 (5·2–5·9)	79 (71–84)	68 (36–109)	23 (1·0 –49)
Namibia	8·4 (7·6–8·8)	85 (79–91)	158 (113–210)	50 (20–88)
Rwanda	1·8 (1·6–2·0)	87 (77–95)	12 (9·2–15)	2·5 (1·1–4·3)
Swaziland	17·4 (16·5–19·2)	96 (88–100)	218 (129–329)	61 (11–125)

Togo	1·5<br>(1·2–1·7)	64<br>(54–79)	6<br>(4·8–7·4)	1·0<br>(0·3–1·8)
United Republic of Tanzania	2·9<br>(2·6–3·1)	75<br>(67–81)	56<br>(27–97)	20<br>(0·0 –48)
Uganda	3·4<br>(3·2–3·6)	84<br>(78–92)	78<br>(46–119)	19<br>(3·0 –39)
South Africa	12·8<br>(11·8–13·7)	70<br>(64–74)	357<br>(248–486)	62<br>(0·0 –168)
Zambia	6·7<br>(5·4–7·3)	85<br>(80–92)	154<br>(100–220)	53<br>(15–99)
Zimbabwe	9·6<br>(8·2–10·9)	85<br>(74–97)	119<br>(88–154)	31<br>(13–53)

9.4 Calibrated *Status-Quo No-New-Vaccine* baseline trends

Each country was calibrated individually to either the nine or thirteen calibration targets as in section 9.3. We investigated the trends in incidence, mortality, and case notifications throughout the entire simulation period (1900–2050) when just fitting to 2019 targets. We observed declining trends in incidence and mortality aligning with the declining incidence and mortality rates predicted by the WHO.

Here we show the tuberculosis incidence and mortality rates plotted from 2000–2050 for the selected grouping for reporting model outcomes. In Figure S9.1, looking by WHO region, we see the incidence rates are highest in AFR and SEAR, and lowest in AMR and EUR. In Figure S9.2, we see that correspondingly, the mortality rates are highest in AFR and SEAR, and lowest in AMR, EUR, and WPR. The estimated model medians for all WHO regions demonstrate decreasing trends from 2000 to 2050.

In Figure S9.3 and Figure S9.4, we show the incidence and mortality rate trends by income group. Both incidence and mortality rates follow a trend with the highest estimated medians in lower-middle-income countries, followed by low-income countries and high-income countries, which aligns with the expectation of burden within each region.

In Figure S9.5 and S9.6, we compare incidence and mortality rates between countries included on the WHO high TB burden list and all other countries modelled, and as expected, higher values are predicted for countries on the high TB burden list.

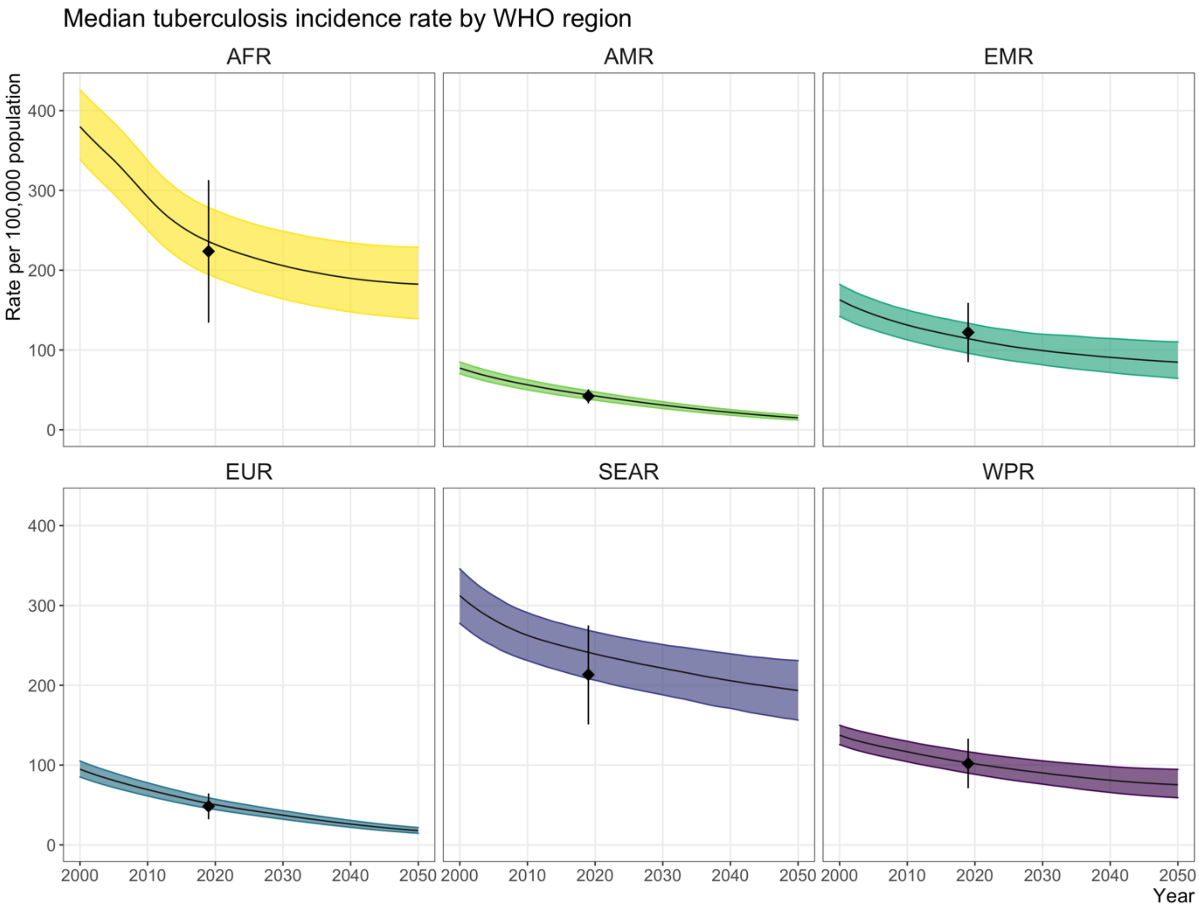

Figure S9.1 Tuberculosis incidence rates for the *Status Quo No-New-Vaccine* baseline by WHO region

The black diamond is the WHO median estimate of the incidence in 2019 for the 105 modelled LMICs by WHO region with 95% uncertainty range. The black line is the model estimated median incidence rate, with shaded 95% uncertainty ranges.

AFR = WHO African Region, AMR = WHO Region of the Americas, EMR = WHO Eastern Mediterranean Region, EUR = WHO European Region, SEAR = WHO South-East Asian Region, WPR = WHO Western Pacific Region

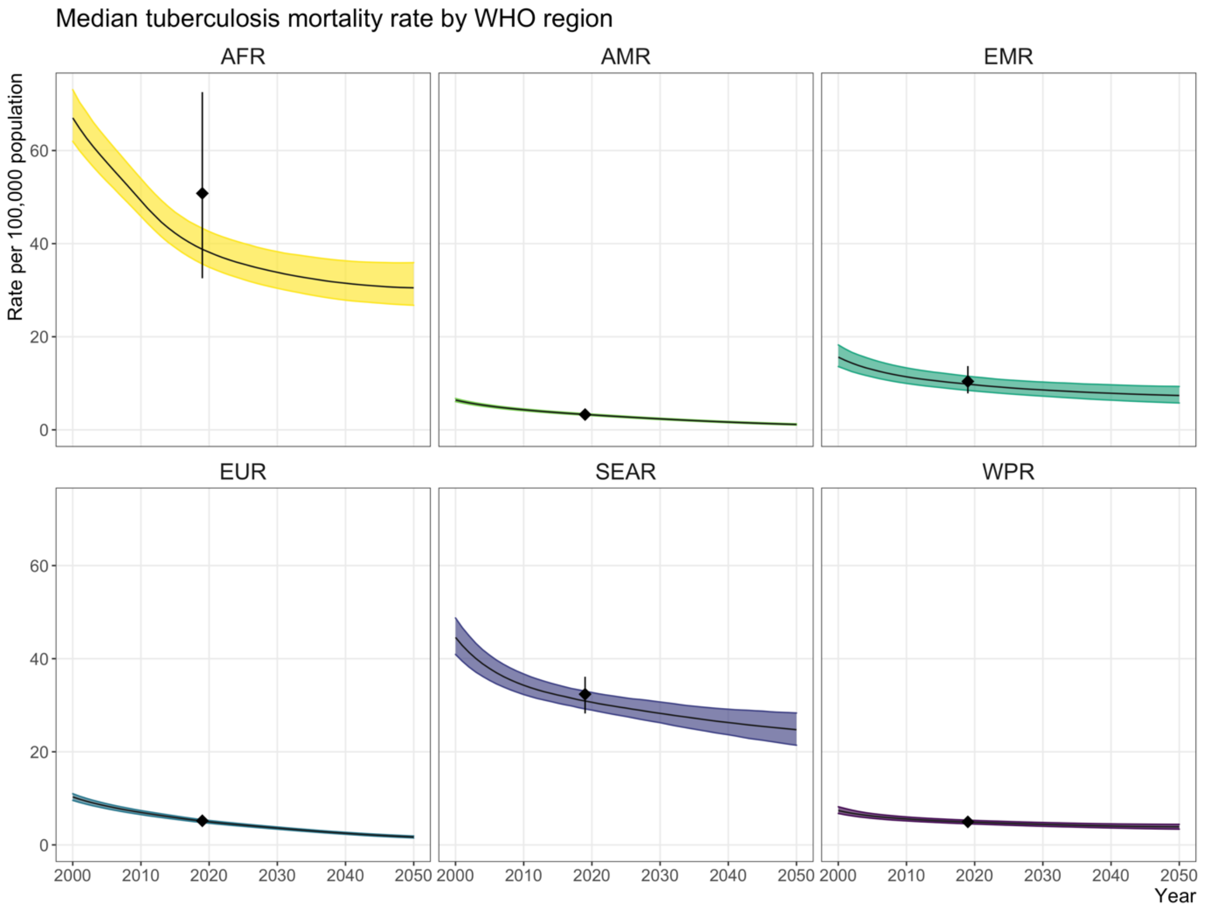

Figure S9.2 Tuberculosis mortality rates for the *Status Quo No-New-Vaccine* baseline by WHO region

The black diamond is the WHO median estimate of the mortality rate in 2019 for the 105 modelled LMICs by WHO region with 95% uncertainty range. The black line is the model estimated median mortality rate, with shaded 95% uncertainty ranges.

AFR = WHO African Region, AMR = WHO Region of the Americas, EMR = WHO Eastern Mediterranean Region, EUR = WHO European Region, SEAR = WHO South-East Asian Region, WPR = WHO Western Pacific Region

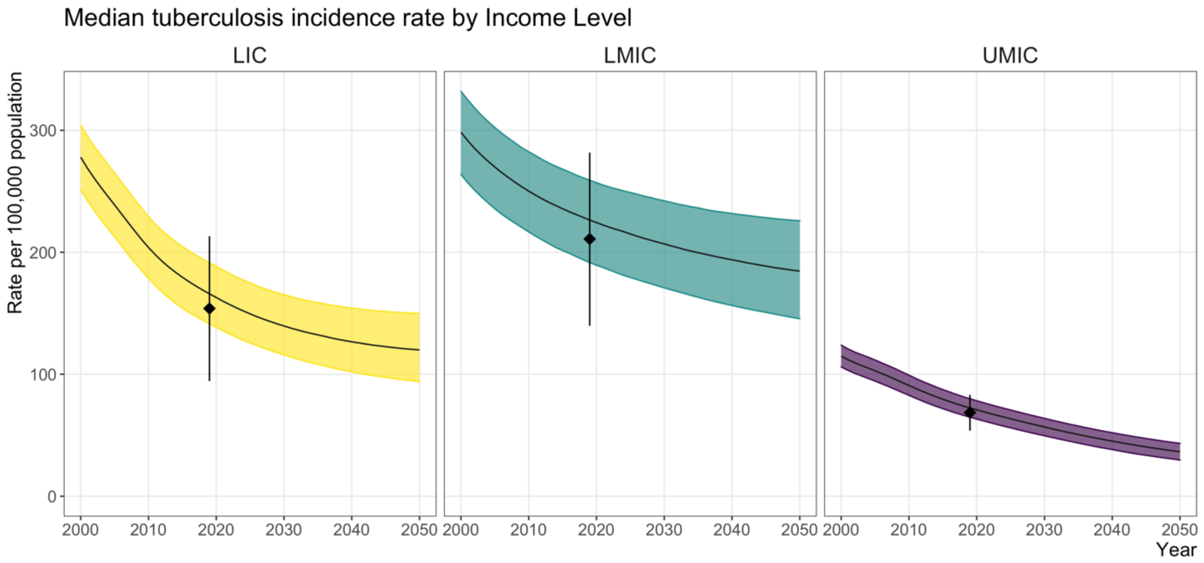

Figure S9.3 Tuberculosis incidence rates for the *Status Quo No-New-Vaccine* baseline by income group

The black diamond is the WHO median estimate of the incidence rate in 2019 for the 105 modelled LMICs by income group with 95% uncertainty range. The black line is the model estimated median incidence rate, with shaded 95% uncertainty ranges.

LIC = low-income countries, LMIC = lower-middle income countries, UMIC = upper-middle income countries

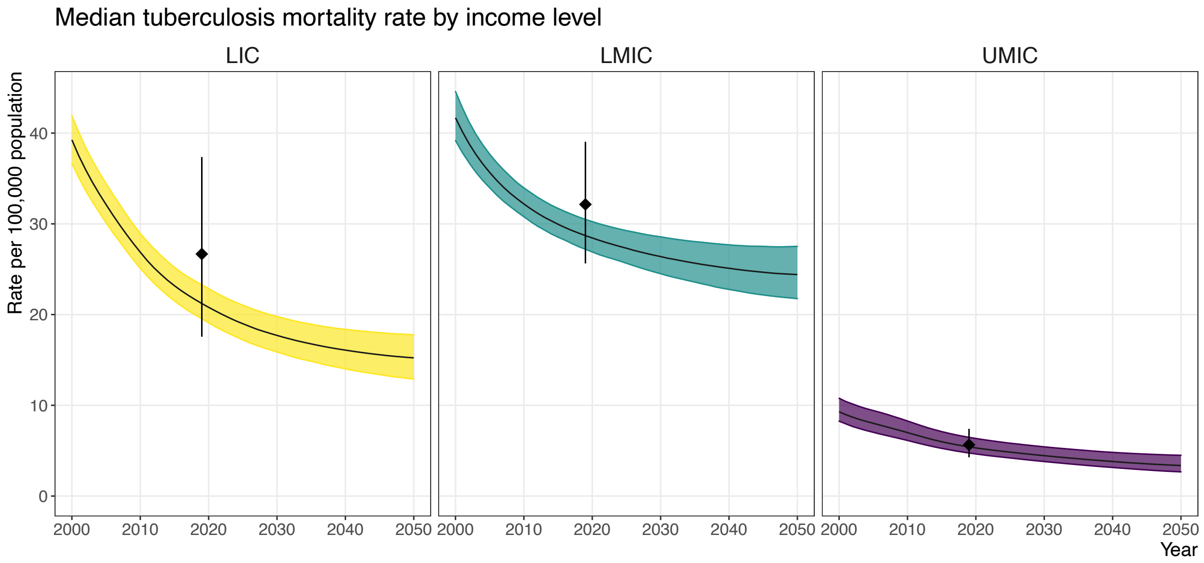

Figure S9.4 Tuberculosis mortality rates for the *Status Quo No-New-Vaccine* baseline by income group

The black diamond is the WHO median estimate of the mortality rate in 2019 for the 105 modelled LMICs by income group with 95% uncertainty range. The black line is the model estimated median mortality rate, with shaded 95% uncertainty ranges.

LIC = low-income countries, LMIC = lower-middle income countries, UMIC = upper-middle income countries

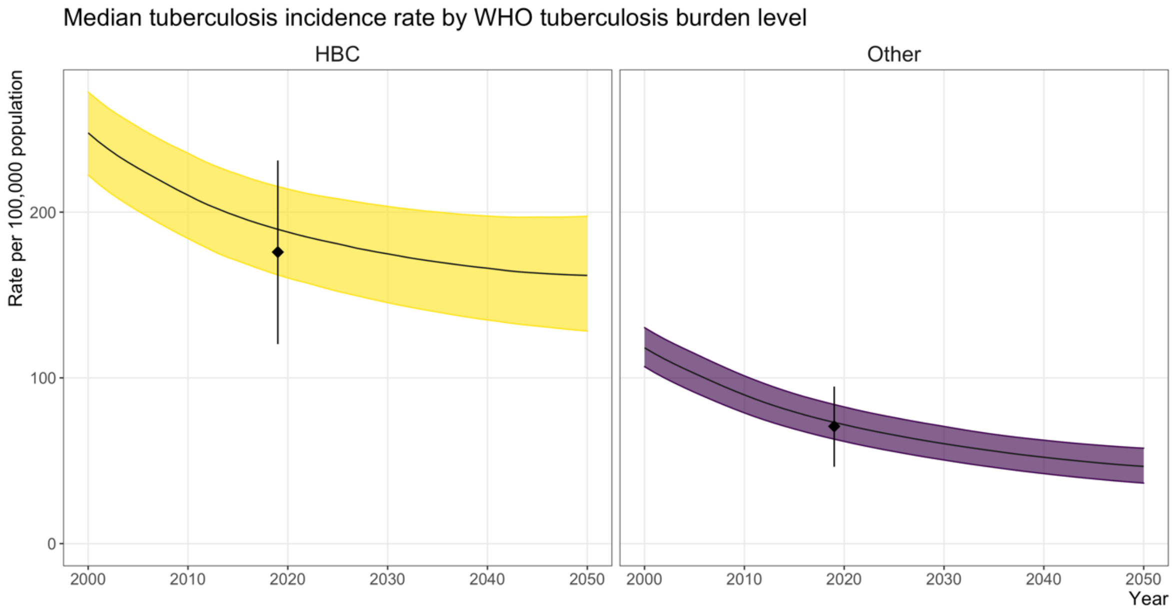

Figure S9.5 **Tuberculosis incidence rates for the *Status Quo No-New-Vaccine* baseline for the countries included on the WHO high-TB-burden list and for all other countries modelled**

The black diamond is the WHO median estimate of the incidence rate in 2019 for the 105 modelled LMICs by burden level with 95% uncertainty range. The black line is the model estimated median incidence rate, with shaded 95% uncertainty ranges.

HBC = high burden countries

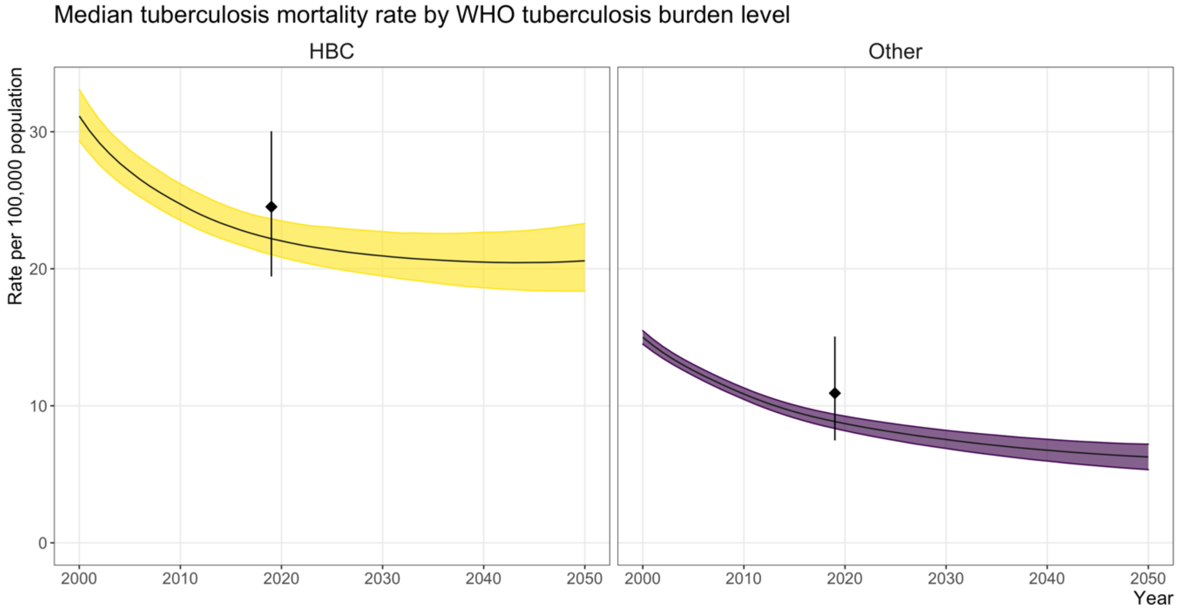

Figure S9.6 **Tuberculosis mortality rates for the *Status Quo No-New-Vaccine* baseline for the countries included on the WHO high-TB-burden list and for all other countries modelled**

The black diamond is the WHO median estimate of the mortality rate in 2019 for the 105 modelled LMICs by burden level with 95% uncertainty range. The black line is the model estimated median mortality rate, with shaded 95% uncertainty ranges.

HBC = high burden countries

10. Vaccine Health Impact Results

10.1 Incidence and mortality rate reductions and cumulative cases, treatments, and deaths averted

As stated in the main text, delivery of a 50% efficacy vaccine with an average of 10-years protection and medium coverage will have a substantial impact, which varies based on delivery and vaccine characteristics. For the adolescent/adult vaccine, compared to the *Basecase* implementation, the *Accelerated Scale-up* scenario averted approximately 60% more cases, treatments, and deaths by 2050, and almost ten times as many as the *Routine Only* scenario, demonstrating the benefits of instantly introducing and scaling-up to coverage, as well as including a campaign for ages ten and over. We performed scenario analyses by varying certain vaccine and delivery characteristics, the results of which are presented in Table S10.1 (adolescent/adult vaccine) and Table S10.2 (infant vaccine) below, as the median estimate and 95% uncertainty range. Decreasing the target vaccine coverage correspondingly decreased the health impact estimates, and increasing the target vaccine coverage, increasing the duration of protection to lifelong, or increasing the vaccine efficacy increases the health impact estimates. The order of vaccine scenario health impact results within each table is as follows:

Primary scenarios (as in main text)

- *Basecase*, medium coverage, 50% efficacy, 10-years protection
- *Accelerated Scale-up*, medium coverage, 50% efficacy, 10-years protection
- *Routine Only*, medium coverage, 50% efficacy, 10-years protection (adolescent/adult vaccine only)

Low and high coverage targets

- *Basecase*, low coverage, 50% efficacy, 10-years protection
- *Basecase*, high coverage, 50% efficacy, 10-years protection
- *Accelerated Scale-up*, low coverage, 50% efficacy, 10-years protection
- *Accelerated Scale-up*, high coverage, 50% efficacy, 10-years protection
- *Routine Only*, low coverage, 50% efficacy, 10-years protection (adolescent/adult vaccine only)
- *Routine Only*, high coverage, 50% efficacy, 10-years protection (adolescent/adult vaccine only)

Lifelong duration of protection

- *Basecase*, medium coverage, 50% efficacy, lifelong protection
- *Accelerated Scale-up*, medium coverage, 50% efficacy, lifelong protection
- *Routine Only*, medium coverage, 50% efficacy, lifelong protection (adolescent/adult vaccine only)

75% efficacy (adolescent/adult vaccine only)

- *Basecase*, medium coverage, 75% efficacy, 10-years protection
- *Accelerated Scale-up*, medium coverage, 75% efficacy, 10-years protection

2025 End TB No-New-Vaccine baseline (adolescent/adult vaccine only)

- *Basecase*, medium coverage, 50% efficacy, 10-years protection
- *Accelerated Scale-up*, medium coverage, 50% efficacy, 10-years protection

Table S10.1 Estimated health impact in 2050 by WHO region, income level, and tuberculosis burden level for the adolescent and adult vaccine scenarios

	Health Impact Measure	All modelled countries	WHO region						WHO tuberculosis burden level		World Bank income group		
**Vaccine Scenario**			**AFR**	**HBC**	**All other countries**	**EUR**	**SEAR**	**WPR**	**HBC**	**All other countries**	**LIC**	**LMIC**	**UMIC**
**Primary scenarios**													
Basecase<br><br>Medium coverage, 50% efficacy, 10 years protection	Averted cases before 2050	44·0m (37·2–51·6)	13·9m (11·7–16·7)	0·5m (0·5–0·6)	3·9m (3·1–4·8)	0·3m (0·3–0·4)	19·5m (15·9–23·1)	5·9m (5·0–6·9)	39·8m (33·7–46·7)	4·1m (3·4–4·9)	5·0m (4·1–6·0)	34·3m (28·6–40·3)	4·7m (4·1–5·4)
	Averted tx before 2050	24·9m (21·9–27·3)	6·3m (5·7–6·8)	0·4m (0·3–0·4)	2·4m (2·0–2·8)	0·2m (0·2–0·3)	11·7m (10·1–13·4)	3·8m (3·3–4·2)	22·6m (19·9–24·8)	2·3m (2·0–2·6)	2·9m (2·5–3·2)	19·0m (16·6–21·2)	2·9m (2·7–3·2)
	Averted deaths before 2050	5·0m (4·6–5·4)	2·1m (1·9–2·3)	0·04m (0·03–0·04)	0·3m (0·2–0·4)	0·028m (0·026–0·031)	2·2m (2·0–2·6)	0·3m (0·2–0·3)	4·5m (4·2–4·9)	0·5m (0·4–0·5)	0·6m (0·5–0·6)	4·1m (3·7–4·4)	0·4m (0·3–0·5)
	IRR in 2050 (%)	25·4% (23·9–27·7)	27·0% (25·7–31·3)	15·9% (15·2–16·9)	26·7% (23·7–31·6)	20·2% (18·6–22·6)	25·4% (23·3–28·2)	19·8% (18·3–22·2)	25·4% (23·8–27·9)	25·1% (24·1–26·6)	27·3% (26·0–29·1)	26·1% (24·3–28·9)	16·7% (15·8–18·0)
	MRR in 2050 (%)	27·1% (25·6–30·1)	27·7% (26·3–33·3)	17·7% (16·8–18·7)	28·1% (25·0–32·8)	19·9% (18·6–21·6)	26·5% (24·3–29·4)	23·1% (21·2–25·8)	27·3% (25·5–30·6)	25·9% (25·0–27·1)	27·8% (26·6–29·4)	27·6% (25·8–31·3)	19·4% (18·1–21·3)
Accelerated Scale-up<br><br>Medium coverage, 50% efficacy, 10 years protection	Averted cases before 2050	65·5m (55·6–76·0)	19·5m (16·7–23·1)	0·8m (0·7–1·0)	5·4m (4·3–6·7)	0·6m (0·5–0·7)	31·0m (25·8–36·4)	8·1m (6·9–9·5)	58·6m (49·9–67·9)	7·0m (5·8–8·2)	7·5m (6·2–9·0)	51·7m (43·6–60·2)	6·4m (5·6–7·2)
	Averted tx before 2050	38·6m (34·4–42·3)	9·2m (8·5–9·9)	0·6m (0·5–0·7)	3·4m (2·9–4·0)	0·4m (0·4–0·5)	19·5m (16·8–22·2)	5·3m (4·8–5·9)	34·6m (30·7–37·9)	4·0m (3·5–4·4)	4·5m (4·0–5·0)	30·0m (26·5–33·3)	4·1m (3·7–4·4)
	Averted deaths before 2050	7·9m (7·3–8·5)	3·1m (2·9–3·4)	0·06m (0·05–0·06)	0·5m (0·4–0·6)	0·06m (0·05–0·06)	3·8m (3·3–4·3)	0·40m (0·36–0·45)	7·0m (6·4–7·6)	0·8m (0·8–0·9)	0·9m (0·8–1·0)	6·5m (5·9–7·0)	0·5m (0·4–0·6)
	IRR in 2050 (%)	25·2% (23·9–27·5)	27·6% (26·3–32·1)	15·2% (14·4–16·2)	27·1% (24·5–31·4)	18·4% (16·4–21·6)	24·7% (22·8–27·3)	19·4% (18·1–21·3)	25·2% (23·8–27·6)	25·3% (24·5–26·8)	27·5% (26·3–29·2)	25·9% (24·3–28·6)	16·3% (15·5–17·3)
	MRR in 2050 (%)	26·7% (25·2–29·9)	28·2% (26·8–34·6)	16·2% (15·3–17·3)	27·9% (25·2–32·3)	18·1% (16·5–20·7)	25·3% (23·2–28·2)	21·8% (20·2–24·3)	26·8% (25·1–30·4)	26·1% (25·3–27·2)	27·7% (26·6–29·2)	27·2% (25·5–31·0)	18·4% (17·3–20·0)
Routine Only<br><br>Medium coverage, 50% efficacy, 10 years protection	Averted cases before 2050	8·8m (7·6–10·1)	3·5m (3·0–3·9)	0·04m (0·03–0·05)	0·9m (0·7–1·2)	0·02m (0·02–0·03)	3·4m (2·6–4·4)	1·0m (0·8–1·2)	8·1m (7·0–9·3)	0·7m (0·6–0·8)	1·1m (0·9–1·3)	7·2m (6·2–8·3)	0·5m (0·4–0·7)
	Averted tx before 2050	4·1m (3·7–4·6)	1·2m (1·1–1·4)	0·03m (0·02–0·03)	0·5m (0·4–0·6)	0·01m (0·01–0·02)	1·8m (1·4–2·2)	0·6m (0·5–0·7)	3·8m (3·4–4·2)	0·3m (0·3–0·4)	0·6m (0·5–0·6)	3·3m (2·9–3·8)	0·3m (0·2–0·3)
	Averted deaths before 2050	1·1m (0·9–1·2)	0·5m (0·4–0·6)	0·003m (0·003–0·004)	0·08m (0·06–0·10)	0·002m (0·002–0·003)	0·4m (0·3–0·5)	0·1m (0·0–0·1)	1·0m (0·8–1·1)	0·08m (0·07–0·09)	0·12m (0·10–0·14)	0·9m (0·7–1·0)	0·1m (0·0–0·1)

	IRR in 2050 (%)	9·9% (9·0–11·6)	11·2% (10·3–14·7)	3·4% (3·1–3·9)	11·9% (9·9–15·3)	4·1% (3·4–5·2)	9·1% (7·8–11·1)	7·7% (6·5–9·5)	10·2% (9·1–12·0)	8·0% (7·3–9·2)	10·5% (9·6–11·9)	10·4% (9·2–12·5)	5·2% (4·4–6·3)
	MRR in 2050 (%)	9·9% (8·9–12·3)	10·7% (9·7–15·2)	3·7% (3·3–4·2)	11·9% (9·9–15·1)	3·8% (3·3–4·5)	8·7% (7·3–10·7)	9·2% (7·5–11·7)	10·2% (9·1–12·9)	7·2% (6·5–8·1)	9·6% (8·8–10·7)	10·2% (9·0–13·1)	6·2% (5·2–7·8)
**Low and high coverage**													
Basecase Low coverage, 50% efficacy, 10 years protection	Averted cases before 2050	33·5m (28·5–39·2)	10·7m (9·1–12·8)	0·4m (0·3–0·5)	3·0m (2·4–3·7)	0·3m (0·2–0·3)	14·7m (12·1–17·5)	4·4m (3·8–5·2)	30·4m (25·7–35·5)	3·1m (2·6–3·7)	3·9m (3·2–4·6)	26·1m (22·0–30·7)	3·5m (3·0–4·0)
	Averted tx before 2050	18·8m (16·6–20·6)	4·8m (4·4–5·1)	0·3m (0·2–0·3)	1·8m (1·5–2·2)	0·2m (0·1–0·2)	8·8m (7·6–10·1)	2·8m (2·5–3·2)	17·0m (15·1–18·8)	1·7m (1·5–1·9)	2·2m (1·9–2·5)	14·4m (12·6–16·0)	2·2m (2·0–2·3)
	Averted deaths before 2050	3·8m (3·5–4·1)	1·6m (1·5–1·8)	0·03m (0·02–0·03)	0·2m (0·2–0·3)	0·021m (0·019–0·023)	1·7m (1·5–1·9)	0·21m (0·18–0·23)	3·5m (3·2–3·8)	0·3m (0·3–0·4)	0·4m (0·4–0·5)	3·1m (2·8–3·4)	0·3m (0·2–0·4)
	IRR in 2050 (%)	20·2% (19·0–22·2)	21·6% (20·5–25·4)	12·2% (11·6–13·0)	21·5% (19·0–25·8)	15·5% (14·2–17·5)	20·1% (18·3–22·6)	15·7% (14·4–17·7)	20·3% (18·9–22·5)	19·7% (18·9–21·0)	21·7% (20·6–23·4)	20·8% (19·3–23·3)	13·0% (12·2–14·1)
	MRR in 2050 (%)	21·5% (20·2–24·2)	22·0% (20·9–27·0)	13·5% (12·8–14·4)	22·5% (19·9–26·6)	15·2% (14·1–16·6)	20·9% (19·0–23·4)	18·3% (16·6–20·7)	21·7% (20·2–24·6)	20·1% (19·3–21·2)	22·0% (20·9–23·4)	22·0% (20·4–25·2)	15·1% (14·0–16·8)
Basecase High coverage, 50% efficacy, 10 years protection	Averted cases before 2050	54·2m (45·7–63·6)	17·0m (14·2–20·5)	0·7m (0·6–0·8)	4·8m (3·8–5·9)	0·4m (0·4–0·5)	24·1m (19·7–28·5)	7·3m (6·2–8·6)	49·1m (41·4–57·6)	5·1m (4·2–6·1)	6·2m (5·0–7·4)	42·1m (35·1–49·7)	6·0m (5·2–6·8)
	Averted tx before 2050	30·8m (27·1–33·9)	7·7m (7·0–8·4)	0·5m (0·4–0·5)	2·9m (2·4–3·4)	0·29m (0·26–0·33)	14·5m (12·5–16·6)	4·7m (4·2–5·3)	27·9m (24·6–30·7)	2·9m (2·5–3·2)	3·5m (3·1–4·0)	23·5m (20·5–26·2)	3·7m (3·4–4·0)
	Averted deaths before 2050	6·1m (5·7–6·7)	2·6m (2·3–2·9)	0·0m (0·0–0·1)	0·4m (0·3–0·5)	0·04m (0·03–0·04)	2·8m (2·4–3·2)	0·3m (0·3–0·4)	5·6m (5·1–6·1)	0·6m (0·5–0·6)	0·7m (0·6–0·8)	5·0m (4·6–5·4)	0·5m (0·4–0·6)
	IRR in 2050 (%)	30·3% (28·6–32·8)	32·1% (30·6–36·7)	19·5% (18·6–20·6)	31·6% (28·2–37·0)	24·6% (22·9–27·3)	30·3% (28·0–33·5)	23·9% (22·2–26·4)	30·3% (28·5–33·0)	30·2% (29·1–31·7)	32·5% (31·0–34·5)	31·1% (29·0–34·1)	20·2% (19·2–21·8)
	MRR in 2050 (%)	32·4% (30·7–35·7)	33·1% (31·6–39·1)	21·7% (20·7–22·9)	33·3% (29·9–38·5)	24·4% (22·9–26·4)	31·8% (29·3–35·1)	27·8% (25·6–30·8)	32·6% (30·6–36·2)	31·4% (30·4–32·7)	33·3% (32·0–35·1)	33·0% (31·0–37·0)	23·5% (22·0–25·7)
Accelerated Scale-up Low coverage, 50% efficacy, 10 years protection	Averted cases before 2050	50·9m (43·4–59·0)	15·3m (13·3–18·1)	0·6m (0·5–0·7)	4·3m (3·4–5·2)	0·5m (0·4–0·6)	24·0m (19·9–28·3)	6·2m (5·3–7·2)	45·6m (38·9–52·8)	5·4m (4·5–6·3)	5·9m (4·9–7·1)	40·2m (34·1–46·8)	4·8m (4·2–5·4)
	Averted tx before 2050	29·7m (26·6–32·6)	7·2m (6·6–7·7)	0·4m (0·4–0·5)	2·6m (2·2–3·1)	0·3m (0·3–0·4)	15·0m (13·0–17·2)	4·0m (3·6–4·5)	26·6m (23·8–29·3)	3·1m (2·7–3·4)	3·5m (3·1–3·9)	23·2m (20·6–25·8)	3·0m (2·8–3·3)
	Averted deaths before 2050	6·1m (5·7–6·6)	2·5m (2·2–2·7)	0·04m (0·04–0·05)	0·4m (0·3–0·4)	0·04m (0·04–0·05)	2·9m (2·5–3·4)	0·3m (0·3–0·3)	5·5m (5·0–6·0)	0·7m (0·6–0·7)	0·7m (0·6–0·8)	5·0m (4·6–5·5)	0·4m (0·3–0·5)

	IRR in 2050 (%)	20·8% (19·5–22·8)	22·7% (21·6–26·8)	12·0% (11·3–12·8)	22·4% (20·2–26·3)	14·9% (13·2–17·8)	20·3% (18·6–22·7)	15·7% (14·6–17·5)	20·8% (19·5–22·9)	20·7% (20·0–22·1)	22·7% (21·6–24·2)	21·4% (19·9–23·8)	13·0% (12·3–13·8)
	MRR in 2050 (%)	21·9% (20·6–24·8)	23·2% (21·9–28·9)	12·8% (12·0–13·7)	23·1% (20·7–27·0)	14·6% (13·2–16·9)	20·7% (18·9–23·4)	17·8% (16·4–20·0)	22·0% (20·6–25·2)	21·2% (20·5–22·3)	22·7% (21·8–24·2)	22·4% (20·9–25·9)	14·7% (13·8–16·1)
Accelerated Scale-up High coverage, 50% efficacy, 10 years protection	Averted cases before 2050	79·6m (67·5–92·6)	23·5m (20·0–28·0)	1·0m (0·9–1·2)	6·5m (5·2–8·1)	0·8m (0·7–0·9)	37·7m (31·4–44·1)	10·0m (8·5–11·7)	71·1m (60·4–82·7)	8·5m (7·1–10·1)	9·1m (7·5–10·8)	62·6m (52·7–73·1)	8·0m (7·0–9·0)
	Averted tx before 2050	47·0m (41·9–51·7)	11·2m (10·2–12·0)	0·7m (0·7–0·8)	4·1m (3·5–4·8)	0·6m (0·5–0·6)	23·7m (20·5–27·0)	6·6m (5·9–7·3)	42·2m (37·4–46·4)	4·9m (4·3–5·4)	5·4m (4·8–6·0)	36·5m (32·3–40·6)	5·1m (4·7–5·5)
	Averted deaths before 2050	9·5m (8·8–10·3)	3·8m (3·5–4·2)	0·1m (0·1–0·1)	0·5m (0·4–0·7)	0·07m (0·06–0·07)	4·6m (4·0–5·2)	0·5m (0·4–0·5)	8·5m (7·8–9·2)	1·0m (0·9–1·1)	1·1m (1·0–1·2)	7·8m (7·2–8·5)	0·6m (0·5–0·8)
	IRR in 2050 (%)	29·4% (28·0–31·9)	32·1% (30·7–37·0)	18·3% (17·2–19·4)	31·4% (28·6–36·1)	21·5% (19·5–25·0)	28·8% (26·7–31·7)	22·9% (21·5–25·0)	29·4% (27·9–32·0)	29·6% (28·7–31·2)	32·0% (30·7–33·9)	30·2% (28·4–33·1)	19·5% (18·6–20·6)
	MRR in 2050 (%)	31·2% (29·6–34·7)	33·0% (31·4–39·8)	19·4% (18·3–20·7)	32·4% (29·4–37·1)	21·4% (19·6–24·2)	29·6% (27·3–32·7)	25·7% (23·8–28·3)	31·3% (29·5–35·1)	30·6% (29·8–31·8)	32·3% (31·2–34·0)	31·8% (29·9–35·9)	21·9% (20·7–23·7)
Routine Only Low coverage, 50% efficacy, 10 years protection	Averted cases before 2050	7·8m (6·8–8·9)	3·1m (2·6–3·5)	0·04m (0·03–0·04)	0·8m (0·6–1·1)	0·02m (0·02–0·03)	3·0m (2·3–3·8)	0·9m (0·7–1·1)	7·2m (6·2–8·2)	0·6m (0·5–0·7)	1·0m (0·8–1·2)	6·3m (5·5–7·3)	0·5m (0·4–0·6)
	Averted tx before 2050	3·6m (3·3–4·0)	1·1m (1·0–1·2)	0·022m (0·019–0·025)	0·4m (0·4–0·6)	0·013m (0·011–0·015)	1·5m (1·3–1·9)	0·5m (0·4–0·6)	3·3m (3·0–3·7)	0·29m (0·26–0·33)	0·5m (0·4–0·6)	2·9m (2·6–3·3)	0·2m (0·2–0·3)
	Averted deaths before 2050	0·9m (0·8–1·1)	0·5m (0·4–0·6)	0·003m (0·002–0·003)	0·1m (0·0–0·1)	0·0019m (0·0017–0·0022)	0·3m (0·3–0·4)	0·1m (0·0–0·1)	0·9m (0·7–1·0)	0·07m (0·06–0·08)	0·1m (0·1–0·1)	0·8m (0·7–0·9)	0·0m (0·0–0·1)
	IRR in 2050 (%)	8·8% (7·9–10·3)	9·9% (9·2–13·1)	3·0% (2·7–3·5)	10·6% (8·7–13·6)	3·6% (3·0–4·6)	8·1% (6·9–9·9)	6·8% (5·8–8·5)	9·0% (8·1–10·6)	7·1% (6·4–8·1)	9·3% (8·5–10·5)	9·2% (8·1–11·1)	4·6% (3·9–5·6)
	MRR in 2050 (%)	8·8% (7·8–10·9)	9·5% (8·6–13·5)	3·3% (2·9–3·7)	10·6% (8·7–13·4)	3·3% (2·9–4·0)	7·7% (6·5–9·5)	8·1% (6·6–10·3)	9·1% (8·0–11·4)	6·3% (5·7–7·2)	8·5% (7·8–9·5)	9·1% (8·0–11·6)	5·5% (4·5–6·9)
Routine Only High coverage, 50% efficacy, 10 years protection	Averted cases before 2050	9·8m (8·5–11·3)	3·9m (3·3–4·4)	0·0m (0·0–0·1)	1·0m (0·8–1·3)	0·03m (0·02–0·03)	3·8m (2·9–4·9)	1·1m (0·9–1·4)	9·1m (7·8–10·4)	0·8m (0·6–0·9)	1·2m (1·0–1·5)	8·0m (6·9–9·3)	0·6m (0·5–0·7)
	Averted tx before 2050	4·6m (4·2–5·1)	1·4m (1·2–1·5)	0·03m (0·02–0·03)	0·6m (0·4–0·7)	0·02m (0·01–0·02)	2·0m (1·6–2·4)	0·6m (0·5–0·8)	4·2m (3·8–4·7)	0·4m (0·3–0·4)	0·6m (0·5–0·7)	3·6m (3·2–4·2)	0·3m (0·3–0·4)
	Averted deaths before 2050	1·2m (1·0–1·3)	0·6m (0·5–0·7)	0·004m (0·003–0·004)	0·08m (0·06–0·11)	0·002m (0·002–0·003)	0·4m (0·3–0·6)	0·07m (0·05–0·09)	1·1m (0·9–1·3)	0·09m (0·07–0·10)	0·1m (0·1–0·2)	1·0m (0·8–1·2)	0·1m (0·0–0·1)
	IRR in 2050 (%)	11·0% (10·0–12·9)	12·5% (11·5–16·3)	3·8% (3·4–4·4)	13·2% (11·0–17·0)	4·6% (3·8–5·8)	10·1% (8·6–12·3)	8·6% (7·3–10·6)	11·3% (10·1–13·3)	8·9% (8·1–10·2)	11·7% (10·7–13·2)	11·5% (10·2–13·8)	5·8% (4·9–7·0)

	MRR in 2050 (%)	11·0% (9·9–13·6)	12·0% (10·9–16·9)	4·1% (3·7–4·7)	13·3% (11·0–16·8)	4·2% (3·6–5·0)	9·7% (8·2–11·9)	10·3% (8·4–13·0)	11·4% (10·1–14·3)	8·0% (7·3–9·0)	10·7% (9·8–11·9)	11·4% (10·0–14·5)	6·9% (5·8–8·7)
**Lifelong protection**													
Basecase Medium coverage, 50% efficacy, lifelong protection	Averted cases before 2050	69·7m (58·3–82·0)	21·9m (18·1–26·6)	0·9m (0·8–1·0)	6·2m (4·9–7·7)	0·6m (0·5–0·6)	30·3m (24·7–35·8)	9·8m (8·3–11·7)	63·2m (52·9–74·5)	6·5m (5·3–7·7)	7·8m (6·4–9·4)	53·7m (44·5–63·7)	8·2m (7·1–9·4)
	Averted tx before 2050	38·4m (33·9–42·4)	9·7m (8·7–10·5)	0·6m (0·5–0·7)	3·7m (3·1–4·4)	0·4m (0·3–0·4)	17·8m (15·3–20·3)	6·2m (5·5–6·9)	34·9m (30·7–38·5)	3·5m (3·1–3·9)	4·4m (3·8–4·9)	29·1m (25·3–32·5)	5·0m (4·5–5·3)
	Averted deaths before 2050	7·5m (6·9–8·2)	3·2m (2·9–3·5)	0·06m (0·05–0·06)	0·5m (0·4–0·6)	0·04m (0·04–0·05)	3·3m (2·9–3·8)	0·4m (0·4–0·5)	6·8m (6·3–7·5)	0·7m (0·6–0·8)	0·8m (0·7–0·9)	6·1m (5·6–6·6)	0·6m (0·5–0·8)
	IRR in 2050 (%)	50·2% (48·1–53·2)	51·4% (49·4–56·5)	40·7% (39·2–42·4)	52·8% (48·4–59·2)	42·7% (40·8–45·2)	50·2% (47·1–53·8)	44·8% (41·9–48·7)	50·6% (48·2–53·8)	47·5% (46·0–49·4)	51·2% (49·2–53·6)	51·0% (48·4–54·7)	42·0% (40·0–44·9)
	MRR in 2050 (%)	49·5% (47·3–53·2)	49·5% (47·5–55·8)	40·8% (39·4–42·5)	52·2% (48·1–58·2)	40·8% (39·1–42·8)	49·3% (46·3–52·9)	45·7% (42·6–49·7)	50·1% (47·7–54·1)	45·1% (43·4–46·8)	48·9% (47·0–51·1)	50·2% (47·6–54·5)	42·9% (40·1–46·6)
Accelerated Scale-up Medium coverage, 50% efficacy, lifelong protection	Averted cases before 2050	116·7m (98·4–136·4)	34·1m (28·4–41·2)	1·6m (1·3–1·8)	9·5m (7·6–11·7)	1·2m (1·0–1·3)	55·3m (45·6–64·8)	15·0m (12·7–17·7)	104·2m (88·0–121·9)	12·4m (10·2–14·7)	13·2m (10·8–15·9)	91·4m (76·3–107·3)	12·1m (10·6–13·7)
	Averted tx before 2050	67·9m (60·1–74·7)	16·1m (14·5–17·3)	1·1m (1·0–1·2)	5·9m (5·0–6·9)	0·8m (0·7–0·9)	34·2m (29·5–38·9)	9·7m (8·6–10·8)	60·9m (53·8–67·2)	7·0m (6·2–7·7)	7·8m (6·8–8·6)	52·5m (46·1–58·5)	7·6m (6·9–8·1)
	Averted deaths before 2050	13·4m (12·4–14·5)	5·2m (4·8–5·8)	0·11m (0·10–0·12)	0·8m (0·6–0·9)	0·10m (0·09–0·11)	6·5m (5·7–7·4)	0·7m (0·6–0·8)	11·9m (11·0–13·0)	1·5m (1·3–1·6)	1·5m (1·4–1·7)	10·9m (10·1–11·9)	0·9m (0·8–1·2)
	IRR in 2050 (%)	55·6% (53·5–58·7)	57·7% (55·8–63·0)	43·4% (41·9–45·1)	57·4% (52·8–64·1)	47·1% (44·6–50·6)	55·7% (52·4–59·6)	47·9% (45·1–51·9)	55·7% (53·3–59·1)	54·9% (53·6–56·8)	58·0% (56·1–60·4)	56·6% (53·9–60·3)	44·4% (42·6–47·2)
	MRR in 2050 (%)	55·7% (53·4–59·8)	56·5% (54·5–63·5)	43·7% (42·2–45·4)	57·1% (52·7–63·3)	46·1% (43·7–49·0)	55·2% (51·8–59·3)	49·4% (46·3–53·5)	55·9% (53·4–60·4)	54·4% (53·1–56·0)	56·7% (54·9–58·9)	56·5% (53·7–61·2)	45·9% (43·3–49·5)
Routine Only Medium coverage, 50% efficacy, lifelong protection	Averted cases before 2050	13·4m (11·6–15·5)	5·3m (4·6–6·1)	0·06m (0·05–0·08)	1·4m (1·1–1·8)	0·04m (0·03–0·05)	5·0m (4·0–6·5)	1·6m (1·2–1·9)	12·4m (10·7–14·4)	1·1m (0·9–1·2)	1·7m (1·4–2·0)	10·9m (9·4–12·7)	0·9m (0·7–1·1)
	Averted tx before 2050	6·2m (5·6–6·9)	1·9m (1·7–2·1)	0·04m (0·03–0·05)	0·8m (0·6–1·0)	0·02m (0·02–0·03)	2·6m (2·1–3·2)	0·9m (0·7–1·1)	5·7m (5·1–6·4)	0·5m (0·4–0·6)	0·8m (0·7–1·0)	4·9m (4·4–5·6)	0·4m (0·4–0·5)
	Averted deaths before 2050	1·5m (1·3–1·7)	0·8m (0·6–0·9)	0·00m (0·00–0·01)	0·11m (0·08–0·15)	0·003m (0·003–0·004)	0·5m (0·4–0·7)	0·08m (0·07–0·11)	1·4m (1·2–1·6)	0·1m (0·1–0·1)	0·2m (0·1–0·2)	1·3m (1·1–1·5)	0·08m (0·06–0·12)
	IRR in 2050 (%)	16·8% (15·3–19·2)	19·2% (18·0–24·0)	6·5% (5·8–7·4)	20·4% (17·2–25·9)	6·8% (5·8–8·3)	15·1% (13·0–18·0)	13·5% (11·6–16·5)	17·2% (15·6–19·8)	13·3% (12·2–15·1)	17·6% (16·2–19·6)	17·5% (15·7–20·4)	9·9% (8·5–11·7)

	MRR in 2050 (%)	15·8% (14·2–19·2)	17·3% (15·9–23·7)	6·3% (5·7–7·1)	19·3% (16·2–24·0)	5·9% (5·2–6·9)	13·7% (11·7–16·5)	14·4% (12·0–17·6)	16·3% (14·7–20·0)	11·3% (10·2–12·7)	15·2% (14·0–16·8)	16·3% (14·5–20·3)	10·9% (9·1–13·5)
**75% efficacy vaccine**													
Basecase Medium coverage, 75% efficacy, 10 years protection	Averted cases before 2050	64·3m (54·0–75·6)	20·3m (17·0–24·6)	0·8m (0·7–0·9)	5·6m (4·5–7·0)	0·5m (0·4–0·6)	28·4m (23·2–33·5)	8·7m (7·3–10·3)	58·2m (49·0–68·5)	6·1m (5·0–7·2)	7·3m (6·0–8·9)	49·8m (41·4–58·9)	7·1m (6·2–8·1)
	Averted tx before 2050	36·4m (32·0–40·1)	9·2m (8·3–10·0)	0·6m (0·5–0·6)	3·4m (2·9–4·0)	0·3m (0·3–0·4)	17·1m (14·7–19·6)	5·6m (4·9–6·3)	33·0m (29·0–36·4)	3·4m (2·9–3·8)	4·2m (3·7–4·7)	27·7m (24·1–30·9)	4·4m (4·0–4·8)
	Averted deaths before 2050	7·3m (6·7–7·9)	3·1m (2·8–3·4)	0·1m (0·0–0·1)	0·4m (0·4–0·6)	0·04m (0·04–0·05)	3·3m (2·9–3·7)	0·04m (0·04–0·05)	6·6m (6·1–7·2)	0·7m (0·6–0·7)	0·8m (0·7–0·9)	5·9m (5·4–6·4)	0·6m (0·5–0·7)
	IRR in 2050 (%)	36·4% (34·5–39·1)	38·7% (37·1–44·0)	23·4% (22·4–24·7)	37·7% (34·1–43·8)	29·1% (27·1–32·1)	36·2% (33·6–39·7)	29·1% (27·2–32·1)	36·4% (34·4–39·4)	36·0% (34·9–37·8)	39·0% (37·4–41·2)	37·3% (35·0–40·7)	24·8% (23·7–26·6)
	MRR in 2050 (%)	38·8% (36·9–42·5)	39·8% (38·2–46·6)	26·0% (24·8–27·3)	39·7% (35·9–45·4)	28·9% (27·3–31·1)	37·9% (35·1–41·4)	33·7% (31·3–37·1)	39·0% (36·9–43·1)	37·4% (36·3–38·9)	39·9% (38·5–41·9)	39·5% (37·2–43·8)	28·9% (27·2–31·6)
Accelerated Scale-up Medium coverage, 75% efficacy, 10 years protection	Averted cases before 2050	95·0m (80·5–110·9)	28·2m (23·9–33·9)	1·2m (1·1–1·4)	7·8m (6·2–9·6)	0·9m (0·8–1·1)	44·8m (37·1–52·5)	12·0m (10·2–14·1)	84·8m (71·8–98·9)	10·1m (8·4–12·0)	10·9m (8·9–13·0)	74·5m (62·6–87·3)	9·6m (8·4–10·9)
	Averted tx before 2050	56·0m (49·7–61·6)	13·4m (12·2–14·5)	0·9m (0·8–1·0)	4·9m (4·1–5·7)	0·7m (0·6–0·7)	28·2m (24·3–32·1)	7·9m (7·0–8·8)	50·2m (44·5–55·3)	5·8m (5·1–6·4)	6·5m (5·7–7·2)	43·4m (38·2–48·2)	6·1m (5·6–6·6)
	Averted deaths before 2050	11·4m (10·5–12·3)	4·5m (4·1–5·0)	0·09m (0·08–0·10)	0·6m (0·5–0·8)	0·08m (0·07–0·09)	5·4m (4·8–6·2)	0·6m (0·5–0·7)	10·1m (9·3–11·0)	1·2m (1·1–1·4)	1·3m (1·2–1·5)	9·3m (8·5–10·1)	0·8m (0·6–1·0)
	IRR in 2050 (%)	35·8% (34·2–38·5)	39·2% (37·6–44·6)	22·0% (20·8–23·2)	37·9% (34·6–43·5)	25·6% (23·3–29·6)	34·9% (32·5–38·2)	28·2% (26·6–30·7)	35·9% (34·1–38·7)	35·8% (34·7–37·4)	38·9% (37·5–40·9)	36·7% (34·7–40·0)	24·1% (23·1–25·3)
	MRR in 2050 (%)	37·8% (36·0–41·7)	40·1% (38·4–47·7)	23·4% (22·1–24·9)	39·1% (35·6–44·5)	25·5% (23·6–28·6)	35·8% (33·2–39·3)	31·6% (29·5–34·8)	37·9% (35·9–42·3)	36·9% (36·0–38·3)	39·2% (37·9–41·1)	38·5% (36·3–43·1)	27·3% (25·9–29·4)
***2025 End TB No-New-Vaccine* baseline**													
Basecase Medium coverage, 50% efficacy, 10 years protection	Averted cases before 2050	10·1m (8·4–12·9)	2·9m (2·3–4·2)	0·19m (0·17–0·22)	0·8m (0·6–1·8)	0·1m (0·1–0·2)	4·0m (3·0–6·4)	1·7m (1·3–2·8)	1·0m (0·8–1·4)	7·1m (5·6–9·8)	1·9m (1·6–2·6)	9·0m (7·3–11·8)	1·1m (0·9–1·2)
	Averted tx before 2050	6·1m (5·0–7·8)	1·5m (1·2–2·0)	0·1m (0·1–0·2)	0·5m (0·4–1·1)	0·10m (0·08–0·13)	2·5m (1·8–4·0)	1·1m (0·9–1·9)	0·6m (0·5–0·8)	4·3m (3·2–5·9)	1·2m (1·0–1·6)	5·4m (4·3–7·2)	0·6m (0·6–0·7)
	Averted deaths before 2050	1·1m (0·8–1·5)	0·4m (0·3–0·7)	0·01m (0·01–0·02)	0·1m (0·0–0·1)	0·01m (0·01–0·02)	0·5m (0·3–0·8)	0·07m (0·05–0·13)	0·1m (0·1–0·2)	0·8m (0·6–1·2)	0·1m (0·1–0·2)	0·9m (0·7–1·4)	0·11m (0·10–0·14)

	IRR in 2050 (%)	12·2% (9·7–16·5)	14·2% (10·2–21·8)	7·8% (6·7–9·4)	11·1% (6·5–20·6)	10·8% (9·1–13·9)	11·7% (8·3–16·7)	9·1% (6·7–16·2)	13·4% (10·5–17·6)	12·4% (9·5–17·7)	9·3% (5·8–14·1)	12·1% (9·3–16·8)	12·9% (11·0–15·3)
	MRR in 2050 (%)	14·5% (11·6–20·1)	15·8% (11·6–25·5)	9·5% (8·2–11·4)	12·9% (7·9–22·2)	11·5% (9·5–14·3)	13·5% (9·9–18·5)	12·2% (9·2–19·4)	15·4% (12·7–19·2)	14·6% (11·2–21·2)	12·0% (7·0–18·0)	14·4% (11·2–20·6)	15·3% (13·3–17·7)
Accelerated Scale-up<br>Medium coverage, 50% efficacy, 10 years protection	Averted cases before 2050	15·4m (12·8–20·0)	4·3m (3·4–6·1)	0·3m (0·3–0·3)	1·2m (0·8–2·6)	0·3m (0·2–0·3)	6·6m (4·8–10·5)	2·4m (1·9–3·8)	1·6m (1·3–2·1)	11·2m (8·8–15·5)	2·6m (2·1–3·5)	13·6m (10·9–18·1)	1·9m (1·6–2·1)
	Averted tx before 2050	9·6m (7·8–12·5)	2·3m (1·8–3·1)	0·2m (0·2–0·2)	0·8m (0·5–1·6)	0·19m (0·16–0·24)	4·3m (3·0–6·9)	1·7m (1·3–2·7)	1·0m (0·8–1·3)	6·9m (5·2–9·7)	1·7m (1·4–2·1)	8·5m (6·6–11·3)	1·1m (1·0–1·3)
	Averted deaths before 2050	1·7m (1·3–2·5)	0·7m (0·5–1·1)	0·02m (0·02–0·03)	0·1m (0·1–0·2)	0·02m (0·02–0·03)	0·8m (0·5–1·4)	0·1m (0·1–0·2)	0·2m (0·1–0·3)	1·3m (1·0–2·0)	0·2m (0·1–0·3)	1·5m (1·1–2·3)	0·2m (0·2–0·3)
	IRR in 2050 (%)	9·2% (6·5–14·0)	12·1% (7·7–21·0)	4·7% (3·4–6·6)	8·8% (3·6–19·5)	5·7% (3·8–9·2)	7·7% (4·1–13·6)	6·8% (4·0–14·7)	10·4% (6·9–15·4)	9·2% (5·8–15·3)	7·7% (4·0–12·6)	9·3% (6·2–14·5)	8·9% (6·5–11·9)
	MRR in 2050 (%)	10·6% (7·3–17·3)	13·0% (8·1–24·9)	5·5% (4·1–7·7)	9·7% (4·2–20·4)	6·3% (4·1–9·7)	8·3% (4·5–14·5)	8·5% (5·2–16·7)	11·3% (7·8–15·9)	10·4% (6·5–18·4)	10·0% (4·6–15·8)	10·7% (7·0–18·0)	10·1% (7·4–13·4)

Abbreviations: AFR = WHO African Region, AMR = WHO Region of the Americas, EMR = WHO Eastern Mediterranean Region, EUR = WHO European Region, HBC = high burden countries, IRR = incidence rate reduction, LIC = low-income countries, LMIC = lower-middle income countries, MRR = mortality rate reduction, SEAR = WHO South-East Asian Region, tx = treatments, UMIC = upper-middle income countries, WPR = WHO Western Pacific Region

Table S10.2 Estimated health impact in 2050 by WHO region, income level, and tuberculosis burden level for the infant vaccine scenarios

	Health Impact Measure	All modelled countries	WHO Region						TB Burden Level		World Bank Income Group		
**Vaccine Scenario**			**AFR**	**AMR**	**EMR**	**EUR**	**SEAR**	**WPR**	**HBC**	**All other countries**	**LIC**	**LMIC**	**UMIC**
Basecase<br><br>Medium coverage, 80% efficacy, 10 years protection	Averted cases before 2050	6·7m (5·8–7·7)	2·9m (2·5–3·4)	0·03m (0·02–0·03)	0·8m (0·6–1·1)	0·02m (0·01–0·02)	2·2m (1·6–2·8)	0·8m (0·6–1·0)	6·2m (5·3–7·1)	0·5m (0·4–0·6)	0·9m (0·7–1·1)	5·4m (4·7–6·2)	0·4m (0·3–0·5)
	Averted tx before 2050	2·7m (2·4–2·9)	0·9m (0·8–0·9)	0·02m (0·01–0·02)	0·4m (0·3–0·5)	0·009m (0·008–0·01)	1·0m (0·8–1·2)	0·4m (0·3–0·5)	2·4m (2·2–2·7)	0·2m (0·2–0·3)	0·4m (0·4–0·5)	2·1m (1·9–2·3)	0·2m (0·1–0·2)
	Averted deaths before 2050	0·9m (0·8–1·0)	0·5m (0·4–0·6)	0·003m (0·002–0·003)	0·08m (0·05–0·11)	0·0018m (0·0015–0·0021)	0·3m (0·2–0·4)	0·1m (0·0–0·1)	0·8m (0·7–1·0)	0·07m (0·06–0·08)	0·11m (0·09–0·13)	0·7m (0·6–0·9)	0·1m (0·0–0·1)
	IRR in 2050 (%)	8·8% (7·9–10·4)	11·0% (10·0–14·5)	2·7% (2·4–3·1)	12·0% (9·7–15·6)	2·9% (2·5–3·4)	6·9% (5·8–8·6)	7·2% (5·9–9·2)	9·0% (8·1–10·7)	7·1% (6·4–8·2)	9·8% (8·9–11·1)	9·1% (8·1–11·1)	4·7% (3·9–5·9)
	MRR in 2050 (%)	9·8% (8·7–12·0)	11·3% (10·1–15·7)	3·7% (3·2–4·3)	13·4% (10·5–18·1)	3·3% (2·9–3·9)	7·2% (5·9–9·6)	11·2% (8·5–15·4)	10·1% (8·9–12·5)	7·1% (6·4–8·0)	9·9% (9·0–11·2)	10·0% (8·7–12·5)	6·6% (5·4–8·5)
Accelerated Scale-up<br><br>Medium coverage, 80% efficacy, 10 years protection	Averted cases before 2050	16·3m (14·0–18·8)	6·3m (5·4–7·2)	0·07m (0·06–0·08)	1·7m (1·3–2·2)	0·07m (0·06–0·09)	6·6m (5·1–8·6)	1·5m (1·2–1·9)	14·7m (12·6–17·1)	1·6m (1·3–1·9)	2·2m (1·8–2·8)	13·3m (11·4–15·5)	0·8m (0·6–0·9)
	Averted tx before 2050	7·7m (6·9–8·6)	2·2m (2·0–2·4)	0·04m (0·04–0·05)	0·9m (0·8–1·2)	0·04m (0·04–0·05)	3·6m (2·9–4·3)	0·9m (0·7–1·0)	6·9m (6·2–7·8)	0·7m (0·7–0·8)	1·1m (1·0–1·3)	6·2m (5·5–7·0)	0·4m (0·3–0·4)
	Averted deaths before 2050	2·3m (2·0–2·6)	1·1m (0·9–1·2)	0·007m (0·006–0·008)	0·2m (0·1–0·2)	0·007m (0·006–0·009)	0·9m (0·7–1·2)	0·1m (0·1–0·2)	2·0m (1·8–2·3)	0·2m (0·2–0·3)	0·3m (0·2–0·3)	1·9m (1·6–2·2)	0·1m (0·1–0·1)
	IRR in 2050 (%)	14·3% (13·0–16·7)	16·7% (15·4–21·6)	4·6% (4·2–5·2)	17·6% (14·5–22·3)	7·5% (6·2–9·8)	12·9% (11·0–15·8)	10·3% (8·7–12·6)	14·4% (13·0–17·0)	13·4% (12·4–14·9)	16·3% (15·1–18·1)	14·9% (13·3–17·9)	6·5% (5·6–7·8)
	MRR in 2050 (%)	15·9% (14·2–19·3)	17·5% (15·9–24·1)	5·8% (5·2–6·6)	19·2% (15·5–24·7)	7·7% (6·5–9·5)	13·4% (11·2–17·0)	15·0% (12·0–19·3)	16·1% (14·3–19·9)	14·1% (13·1–15·3)	16·8% (15·6–18·5)	16·3% (14·4–20·3)	8·9% (7·5–11·1)
**Low and high coverage**													
Basecase<br><br>Low coverage, 80% efficacy, 10 years protection	Averted cases before 2050	6·0m (5·2–6·8)	2·6m (2·2–3·0)	0·02m (0·02–0·03)	0·7m (0·5–1·0)	0·01m (0·01–0·02)	1·9m (1·5–2·5)	0·7m (0·5–0·9)	5·5m (4·7–6·3)	0·5m (0·4–0·6)	0·8m (0·6–1·0)	4·8m (4·2–5·5)	0·4m (0·3–0·5)
	Averted tx before 2050	2·4m (2·2–2·6)	0·8m (0·7–0·8)	0·01m (0·01–0·02)	0·4m (0·3–0·5)	0·008m (0·007–0·009)	0·9m (0·7–1·0)	0·3m (0·3–0·4)	2·2m (2·0–2·4)	0·20m (0·18–0·23)	0·4m (0·3–0·4)	1·8m (1·7–2·1)	0·2m (0·1–0·2)
	Averted deaths before 2050	0·8m (0·7–0·9)	0·4m (0·4–0·5)	0·003m (0·002–0·003)	0·1m (0·0–0·1)	0·002m (0·001–0·002)	0·2m (0·2–0·3)	0·1m (0·0–0·1)	0·7m (0·6–0·8)	0·06m (0·05–0·07)	0·09m (0·08–0·12)	0·7m (0·6–0·8)	0·0m (0·0–0·1)

	IRR in 2050 (%)	7·9% (7·1–9·3)	9·8% (9·0–13·1)	2·4% (2·2–2·8)	10·8% (8·6–14·0)	2·6% (2·3–3·0)	6·2% (5·2–7·7)	6·5% (5·3–8·2)	8·1% (7·2–9·6)	6·3% (5·7–7·3)	8·7% (7·9–9·9)	8·2% (7·2–9·9)	4·2% (3·5–5·2)
	MRR in 2050 (%)	8·7% (7·8–10·7)	10·1% (9·0–14·1)	3·3% (2·9–3·9)	12·0% (9·4–16·2)	2·9% (2·5–3·4)	6·4% (5·2–8·6)	10·0% (7·6–13·8)	9·0% (8·0–11·2)	6·3% (5·7–7·2)	8·8% (8·0–10·0)	8·9% (7·7–11·2)	5·9% (4·8–7·5)
Basecase<br><br>High coverage, 80% efficacy, 10 years protection	Averted cases before 2050	7·4m (6·5–8·6)	3·3m (2·8–3·7)	0·03m (0·03–0·04)	0·9m (0·7–1·2)	0·018m (0·015–0·022)	2·4m (1·8–3·1)	0·9m (0·7–1·1)	6·9m (5·9–7·9)	0·6m (0·5–0·7)	1·0m (0·8–1·2)	6·0m (5·2–6·9)	0·5m (0·4–0·6)
	Averted tx before 2050	3·0m (2·7–3·3)	1·0m (0·9–1·0)	0·017m (0·015–0·019)	0·5m (0·4–0·6)	0·010m (0·009–0·011)	1·1m (0·9–1·3)	0·4m (0·4–0·5)	2·7m (2·5–3·0)	0·3m (0·2–0·3)	0·5m (0·4–0·5)	2·3m (2·1–2·6)	0·2m (0·2–0·2)
	Averted deaths before 2050	1·0m (0·9–1·1)	0·5m (0·4–0·6)	0·003m (0·003–0·004)	0·08m (0·06–0·12)	0·0020m (0·0017–0·0024)	0·3m (0·2–0·4)	0·1m (0·0–0·1)	0·9m (0·8–1·1)	0·08m (0·06–0·09)	0·12m (0·10–0·14)	0·8m (0·7–1·0)	0·1m (0·0–0·1)
	IRR in 2050 (%)	9·7% (8·8–11·4)	12·1% (11·1–16·0)	3·0% (2·7–3·5)	13·3% (10·7–17·1)	3·2% (2·8–3·7)	7·7% (6·4–9·5)	8·0% (6·5–10·1)	9·9% (8·9–11·8)	7·8% (7·0–9·1)	10·8% (9·8–12·2)	10·1% (8·9–12·2)	5·2% (4·4–6·5)
	MRR in 2050 (%)	10·8% (9·6–13·2)	12·5% (11·2–17·3)	4·1% (3·6–4·8)	14·8% (11·6–19·9)	3·7% (3·2–4·3)	8·0% (6·5–10·6)	12·3% (9·4–16·9)	11·2% (9·9–13·8)	7·9% (7·2–8·9)	10·9% (9·9–12·3)	11·1% (9·6–13·8)	7·4% (6·0–9·3)
Accelerated Scale-up<br><br>Low coverage, 80% efficacy, 10 years protection	Averted cases before 2050	14·6m (12·6–16·8)	5·6m (4·8–6·4)	0·06m (0·05–0·07)	1·5m (1·1–2·0)	0·06m (0·05–0·08)	5·9m (4·6–7·7)	1·4m (1·1–1·7)	13·1m (11·3–15·3)	1·4m (1·2–1·7)	2·0m (1·6–2·5)	11·9m (10·2–13·9)	0·7m (0·5–0·8)
	Averted tx before 2050	6·9m (6·2–7·7)	2·0m (1·8–2·2)	0·04m (0·03–0·04)	0·8m (0·7–1·1)	0·04m (0·03–0·05)	3·2m (2·6–3·9)	0·8m (0·6–0·9)	6·2m (5·5–7·0)	0·7m (0·6–0·7)	1·0m (0·9–1·2)	5·5m (4·9–6·3)	0·3m (0·3–0·4)
	Averted deaths before 2050	2·0m (1·8–2·3)	1·0m (0·8–1·1)	0·006m (0·005–0·008)	0·1m (0·1–0·2)	0·006m (0·005–0·008)	0·8m (0·6–1·0)	0·1m (0·1–0·2)	1·8m (1·6–2·1)	0·20m (0·17–0·22)	0·3m (0·2–0·3)	1·7m (1·4–1·9)	0·1m (0·1–0·1)
	IRR in 2050 (%)	12·9% (11·7–15·1)	15·0% (13·9–19·5)	4·1% (3·8–4·7)	15·8% (13·0–20·1)	6·7% (5·5–8·8)	11·6% (9·9–14·2)	9·2% (7·8–11·3)	13·0% (11·6–15·4)	12·0% (11·1–13·5)	14·6% (13·5–16·3)	13·4% (11·9–16·1)	5·8% (5·0–7·0)
	MRR in 2050 (%)	14·3% (12·8–17·4)	15·7% (14·2–21·8)	5·2% (4·7–5·9)	17·3% (13·9–22·3)	6·9% (5·8–8·5)	12·1% (10·1–15·4)	13·5% (10·8–17·4)	14·5% (12·8–18·0)	12·6% (11·7–13·8)	15·1% (14·0–16·7)	14·6% (12·9–18·3)	8·0% (6·7–9·9)
Accelerated Scale-up<br><br>High coverage, 80% efficacy, 10 years protection	Averted cases before 2050	18·0m (15·5–20·7)	6·9m (5·9–7·9)	0·08m (0·07–0·09)	1·9m (1·4–2·4)	0·08m (0·06–0·10)	7·3m (5·7–9·4)	1·7m (1·3–2·1)	16·2m (13·9–18·9)	1·8m (1·5–2·1)	2·5m (2·0–3·1)	14·6m (12·5–17·1)	0·9m (0·7–1·0)
	Averted tx before 2050	8·5m (7·6–9·5)	2·5m (2·2–2·7)	0·05m (0·04–0·05)	1·0m (0·8–1·3)	0·05m (0·04–0·06)	3·9m (3·3–4·8)	0·9m (0·8–1·1)	7·7m (6·9–8·6)	0·8m (0·7–0·9)	1·3m (1·1–1·4)	6·8m (6·1–7·8)	0·4m (0·3–0·5)
	Averted deaths before 2050	2·5m (2·2–2·8)	1·2m (1·0–1·4)	0·008m (0·007–0·009)	0·2m (0·1–0·2)	0·008m (0·007–0·010)	1·0m (0·7–1·3)	0·1m (0·1–0·2)	2·2m (1·9–2·6)	0·2m (0·2–0·3)	0·3m (0·3–0·4)	2·1m (1·8–2·4)	0·1m (0·1–0·1)
	IRR in 2050 (%)	15·7% (14·3–18·3)	18·3% (17·0–23·5)	5·1% (4·7–5·7)	19·3% (15·9–24·3)	8·2% (6·8–10·7)	14·1% (12·1–17·3)	11·3% (9·6–13·8)	15·8% (14·2–18·6)	14·7% (13·6–16·3)	17·8% (16·5–19·8)	16·3% (14·6–19·5)	7·2% (6·1–8·6)

	MRR in 2050 (%)	17·4% (15·7–21·1)	19·2% (17·5–26·2)	6·4% (5·8–7·3)	21·0% (17·1–27·0)	8·4% (7·2–10·4)	14·7% (12·4–18·6)	16·5% (13·3–21·1)	17·7% (15·7–21·7)	15·5% (14·4–16·8)	18·5% (17·1–20·3)	17·9% (15·8–22·2)	9·8% (8·3–12·2)
**Lifelong protection**													
Basecase Medium coverage, 80% efficacy, lifelong protection	Averted cases before 2050	11·8m (10·2–13·7)	5·2m (4·4–6·0)	0·1m (0·0–0·1)	1·4m (1·1–1·8)	0·029m (0·025–0·034)	3·8m (2·9–4·9)	1·4m (1·1–1·8)	10·9m (9·4–12·6)	0·9m (0·8–1·1)	1·5m (1·2–1·9)	9·5m (8·2–11·0)	0·8m (0·6–1·0)
	Averted tx before 2050	4·6m (4·2–5·0)	1·5m (1·3–1·6)	0·029m (0·025–0·032)	0·7m (0·6–0·9)	0·02m (0·01–0·02)	1·7m (1·4–2·0)	0·7m (0·6–0·8)	4·2m (3·8–4·6)	0·4m (0·3–0·4)	0·7m (0·6–0·8)	3·6m (3·2–4·0)	0·3m (0·3–0·4)
	Averted deaths before 2050	1·5m (1·3–1·7)	0·8m (0·7–0·9)	0·01m (0·00–0·01)	0·1m (0·1–0·2)	0·003m (0·003–0·004)	0·5m (0·3–0·6)	0·1m (0·1–0·2)	1·4m (1·2–1·6)	0·11m (0·09–0·13)	0·2m (0·1–0·2)	1·2m (1·1–1·4)	0·1m (0·1–0·1)
	IRR in 2050 (%)	17·4% (15·9–20·1)	21·6% (20·0–27·5)	6·1% (5·5–7·0)	23·4% (19·2–29·6)	5·8% (5·2–6·6)	13·7% (11·7–16·7)	15·1% (12·5–18·8)	17·8% (16·2–20·9)	13·6% (12·3–15·6)	18·8% (17·2–21·2)	18·0% (16·1–21·4)	10·5% (8·8–12·9)
	MRR in 2050 (%)	18·1% (16·2–21·7)	20·9% (18·9–28·2)	7·5% (6·5–8·6)	24·6% (19·7–32·0)	6·4% (5·6–7·3)	13·5% (11·1–17·5)	20·8% (16·1–27·7)	18·7% (16·7–22·7)	12·8% (11·6–14·4)	17·9% (16·4–20·1)	18·5% (16·2–22·7)	13·4% (11·1–16·7)
Accelerated Scale-up Medium coverage, 80% efficacy, lifelong protection	Averted cases before 2050	32·2m (27·5–37·5)	12·0m (10·4–14·1)	0·1m (0·1–0·2)	3·2m (2·4–4·2)	0·1m (0·1–0·2)	13·5m (10·6–17·0)	3·1m (2·5–3·9)	29·1m (24·6–34·0)	3·2m (2·6–3·7)	4·4m (3·6–5·4)	26·2m (22·3–30·9)	1·6m (1·3–2·0)
	Averted tx before 2050	15·3m (13·7–17·1)	4·4m (4·0–4·7)	0·09m (0·08–0·10)	1·8m (1·4–2·2)	0·09m (0·07–0·10)	7·2m (6·0–8·7)	1·7m (1·4–2·0)	13·8m (12·3–15·6)	1·5m (1·3–1·6)	2·2m (1·9–2·5)	12·3m (10·9–14·0)	0·8m (0·7–0·9)
	Averted deaths before 2050	4·2m (3·7–4·8)	2·0m (1·7–2·2)	0·01m (0·01–0·02)	0·3m (0·2–0·4)	0·01m (0·01–0·02)	1·7m (1·3–2·2)	0·2m (0·2–0·3)	3·8m (3·3–4·3)	0·4m (0·4–0·5)	0·5m (0·5–0·6)	3·5m (3·0–4·0)	0·2m (0·1–0·3)
	IRR in 2050 (%)	31·8% (29·4–35·5)	36·2% (34·2–43·3)	11·8% (10·9–13·0)	37·1% (31·7–45·4)	17·1% (14·6–21·3)	29·6% (26·1–34·7)	23·7% (20·4–28·1)	32·1% (29·5–36·2)	29·5% (27·9–32·0)	35·5% (33·4–38·5)	33·1% (30·1–37·7)	15·9% (13·7–18·8)
	MRR in 2050 (%)	33·0% (30·2–38·3)	35·5% (33·0–45·5)	13·4% (12·2–14·9)	38·4% (32·2–47·3)	16·7% (14·6–19·8)	29·2% (25·3–35·1)	30·5% (25·3–37·4)	33·5% (30·3–39·3)	29·3% (27·7–31·4)	34·7% (32·7–37·4)	33·7% (30·4–40·1)	19·8% (16·9–24·0)

Abbreviations: AFR = WHO African Region, AMR = WHO Region of the Americas, EMR = WHO Eastern Mediterranean Region, EUR = WHO European Region, HBC = high burden countries, IRR = incidence rate reduction, LIC = low-income countries, LMIC = lower-middle income countries, MRR = mortality rate reduction, SEAR = WHO South-East Asian Region, tx = treatments, UMIC = upper-middle income countries, WPR = WHO Western Pacific Region

10.2 Absolute differences in numbers averted between scenarios

In Table S10.3 we quantify the absolute difference in the number of cases, treatments, and deaths averted by each of the primary delivery scenarios presented in the main text for the adolescent/adult vaccine and the infant vaccine.

Table S10.3 Absolute differences in cases, treatments, and deaths averted by the primary vaccine delivery scenarios presented in the main text

	Health Impact Measure	All modelled countries	WHO Region						TB Burden Level		World Bank Income Group		
Vaccine Scenario			AFR	AMR	EMR	EUR	SEAR	WPR	HBC	All other countries	LIC	LMIC	UMIC
Adolescent/adult vaccine													
Difference between Accelerated Scale-up scenario and Basecase scenario	Averted cases before 2050	21·5m (4·0–38·8)	5·6m (0·0–11·4)	0·3m (0·1–0·5)	1·5m (-0·5–3·6)	0·3m (0·1–0·4)	11·5m (2·7–20·5)	2·2m (0·0–4·5)	18·8m (3·1–34·3)	2·8m (0·9–4·8)	2·5m (0·2–4·9)	17·4m (3·2–31·6)	1·7m (0·2–3·1)
	Averted tx before 2050	13·7m (7·1–20·4)	2·9m (1·7–4·2)	0·2m (0·1–0·3)	1·0m (0·1–2·0)	0·2m (0·1–0·3)	7·7m (3·3–12·1)	1·6m (0·5–2·6)	12·0m (5·9–18·0)	1·7m (1·0–2·4)	1·6m (0·7–2·4)	11·0m (5·4–16·7)	1·1m (0·6–1·7)
	Averted deaths before 2050	2·9m (1·9–3·9)	1·0m (0·5–1·5)	0·02m (0·01–0·03)	0·1m (0·0–0·3)	0·03m (0·02–0·03)	1·5m (0·7–2·4)	0·1m (0·1–0·2)	2·5m (1·5–3·4)	0·4m (0·2–0·5)	0·3m (0·2–0·5)	2·4m (1·5–3·3)	0·1m (-0·1–0·3)
Difference between Basecase scenario and Routine Only scenario	Averted cases before 2050	35·2m (27·1–44·0)	10·4m (7·8–13·7)	0·5m (0·4–0·6)	3·0m (1·9–4·1)	0·3m (0·3–0·4)	16·1m (11·6–20·4)	4·9m (3·8–6·2)	31·7m (24·3–39·7)	3·4m (2·6–4·3)	3·9m (2·8–5·2)	27·1m (20·3–34·1)	4·2m (3·4–5·0)
	Averted tx before 2050	20·8m (17·3–23·6)	5·1m (4·3–5·7)	0·3m (0·3–0·4)	1·9m (1·4–2·4)	0·2m (0·2–0·2)	10·0m (7·9–12·0)	3·2m (2·7–3·7)	18·8m (15·7–21·4)	2·0m (1·6–2·3)	2·3m (1·9–2·7)	15·7m (12·9–18·3)	2·7m (2·3–2·9)
	Averted deaths before 2050	3·9m (3·4–4·5)	1·6m (1·3–1·9)	0·03m (0·03–0·04)	0·2m (0·1–0·3)	0·03m (0·02–0·03)	1·9m (1·4–2·3)	0·2m (0·2–0·3)	3·6m (3·0–4·1)	0·4m (0·3–0·4)	0·4m (0·4–0·5)	3·2m (2·7–3·7)	0·3m (0·2–0·4)
Difference between Accelerated Scale-up scenario and Routine Only scenario	Averted cases before 2050	56·7m (45·6–68·4)	16·0m (12·8–20·2)	0·8m (0·7–0·9)	4·5m (3·1–6·0)	0·6m (0·5–0·7)	27·6m (21·4–33·8)	7·1m (5·7–8·7)	50·5m (40·5–60·9)	6·3m (5·0–7·7)	6·4m (4·9–8·1)	44·5m (35·3–54·0)	5·9m (4·9–6·8)
	Averted tx before 2050	34·5m (29·8–38·6)	8·0m (7·1–8·8)	0·6m (0·5–0·6)	2·9m (2·2–3·6)	0·4m (0·4–0·5)	17·7m (14·6–20·8)	4·8m (4·1–5·5)	30·8m (26·5–34·5)	3·7m (3·2–4·1)	3·9m (3·3–4·5)	26·7m (22·8–30·4)	3·8m (3·4–4·1)
	Averted deaths before 2050	6·8m (6·1–7·6)	2·6m (2·2–3·0)	0·1m (0·1–0·1)	0·4m (0·3–0·5)	0·1m (0·0–0·1)	3·4m (2·8–4·0)	0·3m (0·3–0·4)	6·1m (5·3–6·8)	0·8m (0·7–0·9)	0·8m (0·7–0·9)	5·6m (4·9–6·3)	0·5m (0·3–0·6)
Infant vaccine													

Difference between Accelerated Scale-up scenario and Basecase scenario	Averted cases before 2050	9·6m (6·3–13·0)	3·3m (2·0–4·7)	0·04m (0·03–0·06)	0·9m (0·2–1·6)	0·05m (0·04–0·08)	4·5m (2·4–6·9)	0·8m (0·2–1·3)	8·5m (5·5–11·8)	1·1m (0·7–1·4)	1·4m (0·7–2·1)	7·9m (5·2–10·8)	0·4m (0·1–0·6)
	Averted tx before 2050	5·0m (4·0–6·2)	1·4m (1·1–1·7)	0·03m (0·02–0·03)	0·5m (0·2–0·9)	0·03m (0·03–0·04)	2·6m (1·8–3·5)	0·5m (0·2–0·7)	4·5m (3·5–5·6)	0·5m (0·4–0·6)	0·7m (0·5–1·0)	4·1m (3·2–5·2)	0·2m (0·1–0·3)
	Averted deaths before 2050	1·4m (1·0–1·8)	0·6m (0·3–0·8)	0·004m (0·003–0·006)	0·1m (0·0–0·2)	0·005m (0·004–0·007)	0·6m (0·3–1·0)	0·1m (0·0–0·1)	1·2m (0·8–1·6)	0·2m (0·1–0·2)	0·2m (0·1–0·3)	1·1m (0·7–1·5)	0·05m (0·0–0·1)

Abbreviations: AFR = WHO African Region, AMR = WHO Region of the Americas, EMR = WHO Eastern Mediterranean Region, EUR = WHO European Region, HBC = high burden countries, LIC = low-income countries, LMIC = lower-middle income countries, SEAR = WHO South-East Asian Region, tx = treatments, UMIC = upper-middle income countries, WPR = WHO Western Pacific Region

10.3 Comparing to the 2035 End TB incidence target

We calculated the incidence rate in 2035 for each *No-New-Vaccine* baseline and for each vaccine scenario to compare with the 2035 End TB incidence target of a 90% reduction in the tuberculosis incidence rate compared to the 2015 incidence rate. Results for all modelled countries, and for each of the select model groupings for outcome reporting are provided in Table S10.4, as the median estimate of the incidence rate per 100,000 population and 95% uncertainty range, with all vaccine scenarios assuming medium coverage and 10-years protection.

For all modelled countries, the estimated incidence rate in 2015 was approximately 164·2 per 100,000 population. A 90% reduction is equivalent to an incidence rate of 16·4 per 100,000 population. With the *Status Quo No-New-Vaccine* baseline, the closest vaccine scenario to reaching this target reduction is the *Accelerated Scale-up* scenario of an adolescent/adult vaccine with vaccine efficacy increased to 75%, which has an estimated incidence rate of 88·1 (70·9–104·7) per 100,000 population or meeting approximately 52% of the goal. With the *2025 End TB No-New-Vaccine* baseline, progress is increased, with the standard *Basecase* scenario of the adolescent/adult vaccine achieving an incidence rate of 42·5 (37·1–50·6) per 100,000 population, and the standard *Accelerated Scale-up* scenario achieving an incidence rate of 43·4 (37·7–51·6) per 100,000 population, or 82% of the target.

Table S10.4 Estimated incidence rate (per 100,000 population) for each vaccine scenario in 2035 to compare to meeting the 2035 End TB target

	All modelled countries	WHO Region						World Bank Income Group			TB Burden Level	
**Scenario**		**AFR**	**AMR**	**EMR**	**EUR**	**SEAR**	**WPR**	**LIC**	**LMIC**	**UMIC**	**HBC**	**All other countries**
Status Quo No-New-Vaccine baseline	138·8 (114·2–163·6)	196·8 (154·9–240·4)	26·2 (22·3–30·0)	94·8 (76·2–117·4)	31·4 (26·8–36·4)	213·2 (179·1–245·2)	85·3 (70·6–101·5)	132·3 (108·2–158·7)	199·6 (163·1–236·4)	50·6 (43·6–57·5)	169·9 (139·7–200·0)	55·9 (46·0–65·9)
Adolescent/adult vaccine: *Basecase*, 50% efficacy	113·5 (93·1–133·9)	155·4 (121·6–191·1)	20·6 (17·4–23·7)	70·9 (56·8–88·3)	26·0 (22·1–30·3)	183·9 (154·4–211·4)	65·2 (53·5–78·3)	110·6 (90·2–133·0)	165·0 (134·6–195·3)	38·1 (32·6–43·6)	138·0 (113·1–162·5)	48·3 (39·6–57·4)
Adolescent/adult vaccine: *Accelerated Scale-up*, 50% efficacy	103·8 (84·1–123·0)	145·4 (110·6–179·3)	21·2 (18·0–24·4)	69·3 (54·9–86·7)	25·0 (21·3–29·3)	157·2 (130·8–180·5)	67·2 (54·8–80·4)	96·9 (78·3–116·9)	147·6 (118·4–175·3)	41·0 (35·0–46·8)	126·9 (102·8–150·2)	42·3 (34·7–50·6)
Adolescent/adult vaccine: *Routine Only*, 50% efficacy	137·3 (112·9–162·0)	193·6 (151·8–236·6)	26·1 (22·2–29·8)	93·2 (74·8–115·4)	31·3 (26·7–36·3)	212·2 (178·3–243·8)	84·1 (69·6–100·4)	130·8 (107·0–156·8)	197·5 (161·3–233·9)	50·1 (43·2–56·9)	168·1 (138·0–197·9)	55·5 (45·7–65·5)
Adolescent/adult vaccine: *Basecase*, 75% efficacy	100·9 (82·7–119·1)	135·1 (105·4–166·1)	17·8 (15·0–20·5)	59·3 (47·4–74·1)	23·2 (19·7–27·2)	169·1 (141·8–194·1)	55·3 (45·1–66·8)	99·9 (81·3–120·5)	148·0 (120·7–174·7)	31·8 (26·9–36·4)	122·2 (100·0–143·6)	44·5 (36·4–53·1)

Adolescent/adult vaccine: *Accelerated Scale-up*, 75% efficacy	88·1 (70·9–104·7)	122·0 (91·2–150·7)	18·8 (15·9–21·7)	58·4 (45·7–72·9)	22·1 (18·7–25·8)	132·4 (110·2–153·1)	58·4 (47·4–70·0)	80·9 (65·1–98·1)	124·3 (99·0–148·4)	36·2 (30·8–41·3)	107·5 (86·4–127·8)	36·1 (29·6–43·4)
Infant vaccine: *Basecase*, 80% efficacy	138·0 (113·5–162·7)	194·8 (153·1–237·9)	26·1 (22·2–29·9)	93·8 (75·3–116·2)	31·3 (26·8–36·4)	212·8 (178·8–244·6)	84·6 (70·1–100·9)	131·5 (107·5–157·7)	198·5 (162·2–235·1)	50·3 (43·4–57·2)	169·0 (138·8–198·9)	55·7 (45·8–65·7)
Infant vaccine: *Accelerated Scale-up*, 80% efficacy	133·1 (109·1–157·0)	186·6 (145·6–228·0)	25·8 (22·0–29·6)	89·0 (71·5–110·6)	30·8 (26·3–35·8)	205·8 (172·8–236·1)	82·8 (68·6–98·9)	125·3 (102·3–150·1)	191·0 (155·9–226·0)	49·7 (42·9–56·5)	163·0 (133·4–192·0)	53·8 (44·2–63·7)
2025 End TB No-New-Vaccine baseline	51·5 (45·0–61·2)	70·2 (58·8–85·5)	12·3 (11·1–13·4)	36·4 (28·3–58·2)	17·4 (14·7–21·2)	73·0 (56·8-100·1)	37·1 (30·2–49·2)	46·9 (40·3–56·1)	69·0 (57·5–87·5)	26·4 (23·5–31·5)	62·0 (53·2–75·2)	23·3 (20·7–26·2)
Adolescent/adult vaccine: *Basecase*, 50% efficacy	42·5 (37·1–50·6)	56·6 (47·5–68·0)	9·8 (8·9–10·7)	28·0 (21·9–44·0)	14·3 (12·2–17·2)	63·1 (49·3–85·9)	29·3 (24·0–38·2)	39·7 (34·0–46·7)	57·7 (48·4–73·2)	20·3 (18·1–24·2)	50·6 (43·4–61·8)	20·4 (18·1–23·0)
Adolescent/adult vaccine: *Accelerated Scale-up*, 50% efficacy	43·4 (37·7–51·6)	58·5 (48·5–70·9)	10·6 (9·6–11·6)	30·5 (23·7–45·7)	15·0 (12·6–18·3)	61·7 (47·9–83·2)	31·8 (26·0–41·4)	39·4 (33·9–46·4)	58·0 (48·2–72·8)	22·7 (20·3–27·0)	52·2 (44·7–63·3)	19·9 (17·7–22·2)

Abbreviations: AFR = WHO African Region, AMR = WHO Region of the Americas, EMR = WHO Eastern Mediterranean Region, EUR = WHO European Region, HBC = high burden countries, IRR = incidence rate reduction, LIC = low-income countries, LMIC = lower-middle income countries, MRR = mortality rate reduction, SEAR = WHO South-East Asian Region, UMIC = upper-middle income countries, WPR = WHO Western Pacific Region

10.4 Averted cases of drug-resistant tuberculosis

Table S10.5 Estimated number of drug-resistant cases averted between vaccine introduction and 2050 for the primary vaccine scenarios for the adolescent and adult vaccine

	All modelled countries	WHO Region						World Bank Income Group			TB Burden Level	
Scenario		AFR	AMR	EMR	EUR	SEAR	WPR	LIC	LMIC	UMIC	HBC	All other countries
Base-case, 50% efficacy	1.4m (1.2–1.6)	0.4m (0.3–0.5)	0.02m (0.01–0.02)	0.1m (0.1–0.2)	0.1m (0.1–0.1)	0.5m (0.4–0.6)	0.2m (0.2–0.2)	0.1m (0.1–0.1)	1.0m (0.8–1.2)	0.3m (0.2–0.3)	1.2m (1.0–1.4)	0.2m (0.1–0.2)
Accelerated Scale-up, 50% efficacy	2.0m (1.7–2.3)	0.5m (0.5–0.6)	0.02m (0.02–0.03)	0.2m (0.2–0.3)	0.1m (0.1–0.2)	0.8m (0.7–1.0)	0.3m (0.3–0.3)	0.1m (0.1–0.2)	1.5m (1.3–1.7)	0.4m (0.3–0.4)	1.7m (1.5–2.0)	0.3m (0.3–0.3)
Routine-only, 50% efficacy	0.3m (0.2–0.3)	0.1m (0.09–0.1)	0.001m (0.001–0.002)	0.03m (0.03–0.05)	0.005m (0.004–0.006)	0.09m (0.07–0.1)	0.02m (0.02–0.03)	0.02m (0.02–0.03)	0.2m (0.2–0.2)	0.03m (0.02–0.03)	0.2m (0.2–0.3)	0.02m (0.02–0.02)

Abbreviations: AFR = WHO African Region, AMR = WHO Region of the Americas, EMR = WHO Eastern Mediterranean Region, EUR = WHO European Region, HBC = high burden countries, IRR = incidence rate reduction, LIC = low-income countries, LMIC = lower-middle income countries, MRR = mortality rate reduction, SEAR = WHO South-East Asian Region, UMIC = upper-middle income countries, WPR = WHO Western Pacific Region

References

1 Frascella B, Richards AS, Sossen B, *et al.* Subclinical tuberculosis disease - a review and analysis of prevalence surveys to inform definitions, burden, associations and screening methodology. *Clin Infect Dis* 2021; **73**: e830–41.

2 Emery JC, Richards AS, Dale KD, *et al.* Self-clearance of Mycobacterium tuberculosis infection: implications for lifetime risk and population at-risk of tuberculosis disease. *Proc R Soc B Biol Sci* 2021; **288**: 20201635.

3 World Health Organization. Guidelines for treatment of drug-susceptible tuberculosis and patient care: 2017 update. 2017.

4 World Health Organization. Treatment of drug-susceptible tuberculosis: rapid communication. Geneva: World Health Organization, 2021 https://apps.who.int/iris/handle/10665/341729 (accessed Nov 2, 2022).

5 Sutherland I, Svandová E, Radhakrishna S. The development of clinical tuberculosis following infection with tubercle bacilli. 1. A theoretical model for the development of clinical tuberculosis following infection, linking from data on the risk of tuberculous infection and the incidence of clinical tuberculosis in the Netherlands. *Tubercle* 1982; **63**: 255–68.

6 Vynnycky E, Fine PE. The natural history of tuberculosis: the implications of age-dependent risks of disease and the role of reinfection. *Epidemiol Infect* 1997; **119**: 183–201.

7 World Health Organization. Global tuberculosis report 2021. Geneva: World Health Organization, 2021 https://apps.who.int/iris/handle/10665/346387 (accessed Nov 2, 2022).

8 Lee JY. Diagnosis and Treatment of Extrapulmonary Tuberculosis. *Tuberc Respir Dis* 2015; **78**: 47–55.

9 Prem K, Zandvoort K van, Klepac P, *et al.* Projecting contact matrices in 177 geographical regions: An update and comparison with empirical data for the COVID-19 era. *PLOS Comput Biol* 2021; **17**: e1009098.

10 Kwan CK, Ernst JD. HIV and Tuberculosis: a Deadly Human Syndemic. *Clin Microbiol Rev* 2011; **24**: 351–76.

11 Suthar AB, Lawn SD, del Amo J, *et al.* Antiretroviral Therapy for Prevention of Tuberculosis in Adults with HIV: A Systematic Review and Meta-Analysis. *PLoS Med* 2012; **9**: e1001270.

12 Harling G, Ehrlich R, Myer L. The social epidemiology of tuberculosis in South Africa: A multilevel analysis. *Soc Sci Med* 2008; **66**: 492–505.

13 Hoa NB, Tiemersma EW, Sy DN, *et al.* Household expenditure and tuberculosis prevalence in Viet Nam: prediction by a set of household indicators. *Int J Tuberc Lung Dis* 2011; **15**: 32–7.

14 Hossain S, Quaiyum MA, Zaman K, *et al.* Socio Economic Position in TB Prevalence and Access to Services: Results from a Population Prevalence Survey and a Facility-Based Survey in Bangladesh. *PLOS ONE* 2012; **7**: e44980.

15 Kapata N, Chanda-Kapata P, Ngosa W, *et al.* The Prevalence of Tuberculosis in Zambia: Results from the First National TB Prevalence Survey, 2013–2014. *PLOS ONE* 2016; **11**: e0146392.

16 Migambi P, Gasana M, Uwizeye CB, *et al.* Prevalence of tuberculosis in Rwanda: Results of the first nationwide survey in 2012 yielded important lessons for TB control. *PLOS ONE* 2020; **15**: e0231372.

17 Oxlade O, Murray M. Tuberculosis and Poverty: Why Are the Poor at Greater Risk in India? *PLOS ONE* 2012; **7**: e47533.

18 Singh SK, Kashyap GC, Puri P. Potential effect of household environment on prevalence of tuberculosis in India: evidence from the recent round of a cross-sectional survey. *BMC Pulm Med* 2018; **18**: 66.

19 Siroka A, Law I, Macinko J, *et al.* The effect of household poverty on tuberculosis. *Int J Tuberc Lung Dis Off J Int Union Tuberc Lung Dis* 2016; **20**: 1603–8.

20 van Leth F, Guilatco RS, Hossain S, *et al.* Measuring socio-economic data in tuberculosis prevalence surveys. *Int J Tuberc Lung Dis* 2011; **15 Suppl 2**: 58–63.

21 Yates TA, Ayles H, Leacy FP, *et al.* Socio-economic gradients in prevalent tuberculosis in Zambia and the Western Cape of South Africa. *Trop Med Int Health* 2018; **23**: 375–90.

22 Foster N, Nguyen HV, Nguyen NV, *et al.* Social determinants of the changing tuberculosis prevalence in Việt Nam: Analysis of population-level cross-sectional studies. *PLOS Med* 2022; **19**: e1003935.

23 United Nations, Department of Economic and Social Affairs, Population Division. World Population Projections [2019 Revision]. 2019. https://population.un.org/wpp/Download/Standard/Population/ (accessed Nov 2, 2022).

24 Tiemersma EW, Werf MJ van der, Borgdorff MW, Williams BG, Nagelkerke NJD. Natural History of Tuberculosis: Duration and Fatality of Untreated Pulmonary Tuberculosis in HIV Negative Patients: A Systematic Review. *PLOS ONE* 2011; **6**: e17601.

25 Quaife M, Houben RMGJ, Allwood B, *et al.* Post-tuberculosis mortality and morbidity: valuing the hidden epidemic. *Lancet Respir Med* 2020; **8**: 332–3.

26 World Health Organization. WHO TB burden estimates. CSV Files to Download. 2022. https://www.who.int/tb/country/data/download/en/ (accessed Nov 2, 2022).

27 World Health Organization. Case Notifications. CSV Files to Download. 2022. https://www.who.int/tb/country/data/download/en/ (accessed Nov 2, 2022).

28 Emery JC, Dodd PJ, Banu S, et al. Estimating the contribution of subclinical tuberculosis disease to transmission – an individual patient data analysis from prevalence surveys. medRxiv. 2022 Jan 1;2022.06.09.22276188.

29 Abu-Raddad L, Sabatelli L, Achterberg JT, *et al.* Epidemiological benefits of more-effective tuberculosis vaccines, drugs, and diagnostics. *Proc Natl Acad Sci U S A* 2009; **106**: 13980–5.

30 Dye C, Williams BG. Eliminating human tuberculosis in the twenty-first century. *J R Soc Interface* 2008; **5**: 653–62.

31 Marx FM, Dunbar R, Enarson DA, *et al.* The Temporal Dynamics of Relapse and Reinfection Tuberculosis After Successful Treatment: A Retrospective Cohort Study. *Clin Infect Dis* 2014; **58**: 1676–83.

32 Gomes MGM, Franco AO, Gomes MC, Medley GF. The reinfection threshold promotes variability in tuberculosis epidemiology and vaccine efficacy. *Proc R Soc B Biol Sci* 2004; **271**: 617–23.

33 Dangisso MH, Woldesemayat EM, Datiko DG, Lindtjørn B. Long-term outcome of smear-positive tuberculosis patients after initiation and completion of treatment: A ten-year retrospective cohort study. *PloS One* 2018; **13**: e0193396.

34 World Health Organization. Treatment Outcomes. CSV Files to Download. 2022. https://www.who.int/tb/country/data/download/en/ (accessed Nov 2, 2022).

35 Gabriela M. Gomes M, Rodrigues P, Hilker FM, *et al.* Implications of partial immunity on the prospects for tuberculosis control by post-exposure interventions. *J Theor Biol* 2007; **248**: 608–17.

36 Mberi MN, Kuonza LR, Dube NM, Nattey C, Manda S, Summers R. Determinants of loss to follow-up in patients on antiretroviral treatment, South Africa, 2004–2012: a cohort study. *BMC Health Serv Res* 2015; **15**: 259.

37 Cutsem GV, Ford N, Hildebrand K, *et al.* Correcting for Mortality Among Patients Lost to Follow Up on Antiretroviral Therapy in South Africa: A Cohort Analysis. *PLOS ONE* 2011; **6**: e14684.

38 Johansson KA, Robberstad B, Norheim OF. Further benefits by early start of HIV treatment in low income countries: Survival estimates of early versus deferred antiretroviral therapy. *AIDS Res Ther* 2010; **7**: 3.

39 Fatti G, Mothibi E, Meintjes G, Grimwood A. Antiretroviral treatment outcomes amongst older adults in a large multicentre cohort in South Africa. *PloS One* 2014; **9**: e100273.

40 Vazquez F. A systematic review and meta-analysis of the effect of HIV status on the incidence of tuberculosis disease among individuals with latent Mycobacterium tuberculosis infection. [Masters Dissertation]. 2019.

41 Ackah AN, Coulibaly D, Digbeu H, *et al.* Response to treatment, mortality, and CD4 lymphocyte counts in HIV-infected persons with tuberculosis in Abidjan, Côte d'Ivoire. *Lancet Lond Engl* 1995; **345**: 607–10.

42 Mukadi YD, Maher D, Harries A. Tuberculosis case fatality rates in high HIV prevalence populations in sub-Saharan Africa. *AIDS* 2001; **15**: 143–52.

43 Rajagopalan S. Tuberculosis and aging: a global health problem. *Clin Infect Dis Off Publ Infect Dis Soc Am* 2001; **33**: 1034–9.

44 Andrianakis I, Vernon I, McCreesh N, *et al.* History matching of a complex epidemiological model of human immunodeficiency virus transmission by using variance emulation. *J R Stat Soc Ser C Appl Stat* 2017; **66**: 717–40.

45 Andrianakis I, Vernon IR, McCreesh N, *et al.* Bayesian History Matching of Complex Infectious Disease Models Using Emulation: A Tutorial and a Case Study on HIV in Uganda. *PLOS Comput Biol* 2015; **11**: e1003968.

46 Goldstein M. Bayes Linear Analysis for Complex Physical Systems Modeled by Computer Simulators. In: Dienstfrey AM, Boisvert RF, eds. Uncertainty Quantification in Scientific Computing. Berlin, Heidelberg: Springer Berlin Heidelberg, 2012: 78–94.

47 Williamson D, Goldstein M, Allison L, *et al.* History matching for exploring and reducing climate model parameter space using observations and a large perturbed physics ensemble. *Clim Dyn* 2013; **41**: 1703–29.

48 Iskauskas A. hmer: History Matching and Emulation Package. 2022. https://CRAN.R-project.org/package=hmer (accessed Nov 2, 2022).

49 Jabot F, Faure T, Dumoulin N, Albert C. EasyABC: Efficient Approximate Bayesian Computation Sampling Schemes. 2015. https://CRAN.R-project.org/package=EasyABC (accessed Nov 2, 2022).

50 Roberts GO, Rosenthal JS. Examples of Adaptive MCMC. *J Comput Graph Stat* 2009; **18**: 349–67.

51 The World Bank. World Bank Country and Lending Groups: Historical classification by income in XLSX format. 2022. https://datahelpdesk.worldbank.org/knowledgebase/articles/906519 (accessed Nov 2, 2022).

52 UNAIDS. HIV estimates with uncertainty bounds 1990-Present. 2022. https://www.unaids.org/en/resources/documents/2022/HIV_estimates_with_uncertainty_bounds_1990-present (accessed Nov 2, 2022).

53 World Health Organization. WHO TB incidence estimates disaggregated by age group, sex and risk factor. CSV Files to Download. 2022. https://www.who.int/tb/country/data/download/en/ (accessed Nov 2, 2022).

54 World Health Organization. WHO | WHO End TB Strategy. 2015.

55 Geneva: World Health Organization. WHO Preferred Product Characteristics for New Tuberculosis Vaccines. 2018 Licence: CC BY-NC-SA 3.0 IGO.

56 Gavi The Vaccine Alliance. Country hub. 2022. https://www.gavi.org/programmes-impact/country-hub (accessed Nov 2, 2022).

57 UNICEF Data. Immunization. 2022. https://data.unicef.org/topic/child-health/immunization/ (accessed Nov 2, 2022).

58 Harris RC, Sumner T, Knight GM, Zhang H, White RG. Potential impact of tuberculosis vaccines in China, South Africa, and India. *Sci Transl Med* 2020; **12**: eaax4607.

59 Tait DR, Hatherill M, Van Der Meeren O, *et al.* Final Analysis of a Trial of M72/AS01E Vaccine to Prevent Tuberculosis. *N Engl J Med* 2019; **381**: 2429–39.

60 Abubakar I, Pimpin L, Ariti C, *et al.* Systematic review and meta-analysis of the current evidence on the duration of protection by bacillus Calmette–Guérin vaccination against tuberculosis. *Health Technol Assess* 2013; **17**. DOI:10.3310/hta17370.

61 Lewnard JA, Grad YH. Vaccine waning and mumps re-emergence in the United States. *Sci Transl Med* 2018; **10**: eaao5945.

62 Blackwood JC, Cummings DAT, Broutin H, Iamsirithaworn S, Rohani P. Deciphering the impacts of vaccination and immunity on pertussis epidemiology in Thailand. *Proc Natl Acad Sci U S A* 2013; **110**: 9595–600.

63 World Health Organization. Countries. 2022. https://www.who.int/countries (accessed Nov 2, 2022).

64 World Health Organization. WHO releases new global lists of high-burden countries for TB, HIV-associated TB and drug-resistant TB. 2021. https://www.who.int/news/item/17-06-2021-who-releases-new-global-lists-of-high-burden-countries-for-tb-hiv-associated-tb-and-drug-resistant-tb (accessed Nov 2, 2022).

65 Scarponi D, Iskauskas A, Clark RA, *et al.* Demonstrating multi-country calibration of a tuberculosis model using new history matching and emulation package - hmer. medRxiv, 2022 DOI:10.1101/2022.05.13.22275052.
